# Antioxidants as Therapeutic Tools in the Management of COPD: A Systematic Review with Meta-Analysis

**DOI:** 10.3390/antiox15040446

**Published:** 2026-04-02

**Authors:** Manuel López-Denis, Bernardo Cálamo-Guzmán, Silvia Castillo-Corullón, Joaquín Carrasco-Luna, María José Herrero, Cruz González-Villaescusa, Jaime Signes-Costa, Francisco Dasí

**Affiliations:** 1 Faculty of Nursing and Podiatry, University of Valencia, C/Menéndez y Pelayo, 19, 46010 Valencia, Spain; malode2@alumni.uv.es; 2 Department of Physiology, Faculty of Medicine, University of Valencia, Avda. Blasco Ibáñez, 13, 46010 Valencia, Spain; bercaguz@alumni.uv.es; 3 Pathology Department, Hospital Universitario de Navarra, C/Irunlarrea, 3, 31008 Pamplona, Spain; 4 Department of Pediatrics, Obstetrics, and Gynecology, Faculty of Medicine, University of Valencia, Avda. Blasco Ibáñez, 13, 46010 Valencia, Spain; castillo_sil@gva.es (S.C.-C.); joaquin.carrasco@uv.es (J.C.-L.); 5 Pediatrics Unit, IIS INCLIVA, Hospital Clínico Universitario de Valencia, Avda. Blasco Ibáñez, 17, 46010 Valencia, Spain; 6 Department of Biotechnology, Faculty of Experimental Sciences and Veterinary, Universidad Católica de Valencia, C/Guillem de Castro, 96, 46001 Valencia, Spain; maria.jose.herrero@uv.es; 7 Department of Pharmacology, Faculty of Medicine, University of Valencia, Avda. Blasco Ibáñez, 13, 46010 Valencia, Spain; 8 Pharmacogenetics, Instituto Investigación Sanitaria La Fe, Avenida Fernando Abril Martorell, 106, 46026 Valencia, Spain; 9 Pulmonology Unit, IIS INCLIVA, Hospital Clínico Universitario de Valencia, Avda. Blasco Ibáñez, 17, 46010 Valencia, Spain; cruz.gonzalez@uv.es (C.G.-V.); signes_jai@gva.es (J.S.-C.)

**Keywords:** antioxidants, acetylcysteine, COPD, oxidative stress, inflammation

## Abstract

Chronic obstructive pulmonary disease (COPD) is a leading cause of morbidity and mortality worldwide. Beyond established risk factors such as smoking and exposure to pollutants increasing evidence emphasizes the role of oxidative stress (OS) in COPD pathophysiology. OS contributes to chronic inflammation, to the progression of the disease and affects both lung function and exacerbations, which opens a rationale for the use of antioxidant and redox-modulating substances in the treatment of the disease. Although numerous substances with antioxidant capacity have been evaluated in randomized clinical trials (RCTs), their clinical relevance remains uncertain. Therefore, a systematic review was conducted to evaluate the effects of these therapies in COPD. Also, a meta-analysis to evaluate the effects on exacerbations was performed. Nineteen RCTs meet the eligibility criteria and were included in the study. Quantitative analyses were performed using random-effects models. N-acetylcysteine-based interventions were associated with a significant reduction in exacerbation risk (risk ratio 0.80; 95% confidence interval 0.66–0.98), corresponding to a 20% relative reduction. No study reported serious adverse effects. These findings suggest that antioxidant-based strategies may have clinically meaningful benefits in COPD. However, larger, more robust RCTs are required to confirm these results and establish optimal therapeutic strategies.

## 1. Introduction

Chronic Obstructive Pulmonary Disease (COPD) is a chronic disease characterized by persistent limitation to air flux and chronic respiratory inflammation leading to progressive loss of lung function. COPD is the third cause of morbidity and mortality worldwide, affecting more than 400 million people [1] and causing 3.5 million deaths in 2021 [2]. COPD pathophysiology is in many aspects still unknown. Although cigarette smoking and exposure to air contaminants are the most important risk factors, oxidative stress (OS) has also been identified as an important mechanism in COPD development, progression, and exacerbations [3,4]. OS is defined as the imbalance between reactive oxygen species (ROS) and the body’s antioxidant mechanisms. Exogenous chronic exposure to cigarette smoke, in combination with in-house and environmental pollution, certain occupational exposures, and asthma, induces ROS overproduction that overwhelms antioxidant defenses, generating a cellular oxidant milieu that may initiate inflammation. In patients with COPD, inflammatory cells, including macrophages, neutrophils, eosinophils, and lymphocytes, are recruited to the lungs. These cells are also an important source of OS in the lungs and, importantly, produce multiple mediators of inflammation that perpetuate inflammation. In these patients, lung epithelial and endothelial cells also produce increased ROS levels. Altogether, these mechanisms lead to lung tissue damage and the characteristic remodeling of the disease [3]. OS contributes not only to chronic inflammation but also to increased resistance to corticosteroid therapies, accelerated senescence, and diminished tissue repair capacity, thereby accelerating the decline in lung function typical of the disease [3,4]. In addition, OS biomarkers such as malondialdehyde, 8-isoprostanes, and reduced glutathione have been associated with poor outcomes and increased exacerbation frequency [5,6,7]. Importantly, oxidative imbalance in COPD arises not only from exogenous oxidants such as cigarette smoke, but also from endogenous sources including mitochondrial dysfunction and chronic inflammatory activation. Consequently, therapeutic strategies targeting oxidative stress go beyond conventional pharmacological agents and include a wide range of interventions with redox-modulating properties. Therefore, both pharmacological compounds (e.g., thiol-based agents), nutraceuticals and plant-derived antioxidants (e.g., polyphenols and carotenoids), and metabolic interventions (e.g., iron repletion) and non-pharmacological approaches such as structured exercise have been shown to influence oxidative balance and antioxidant capacity.

Overall, these observations support the role of OS in the pathophysiology and the prognosis of the disease. Over the years, many studies have been performed to unravel the role of OS in the disease, including the use of antioxidant substances, REDOX modulators, and life-style modifications. However, clinical results have been variable, and many questions about which interventions offer a real clinical benefit remain unanswered. Consequently, the current study represents a comprehensive approach, including antioxidant interventions evaluated in randomized controlled trials (RCTs), in order to evaluate the full spectrum of redox-targeted strategies in COPD.

Therefore, this study was conducted to answer the following research question: “What effective therapeutic strategies currently exist to address OS in COPD?”

## 2. Materials and Methods

### 2.1. Study Design

The PICO system was used to formulate the research question as follows:

- P (population or patients): adult patients with clinically diagnosed COPD.
- I (intervention): effective antioxidant or redox-modulating strategies.
- C (comparison): placebo or standard COPD treatment.
- O (outcomes): exacerbation risk, exercise capacity, and oxidative stress or inflammatory biomarkers.

Importantly, the comparator did not include healthy individuals, but rather COPD patients receiving placebo or standard therapy, in line with the design of the included RCTs.

To answer the research question, a systematic review was conducted following the recommendations of the PRISMA 2020 (Preferred Reporting Items for Systematic Reviews and Meta-Analyses) guidelines [8,9]. The literature search was conducted between January 2020 and December 2025 in the electronic databases PubMed, Scopus, EMBASE, the Cochrane Library, and ClinicalTrials.gov. This temporal restriction was intentionally applied to focus on the most recent articles, reflecting current therapeutic approaches and emerging antioxidant strategies in COPD. Earlier RCTs have been extensively evaluated in previous systematic reviews and meta-analyses. Therefore, the present study aimed to provide an updated synthesis of contemporary evidence rather than a historical overview of antioxidant therapies.

Combinations of the following keywords and search terms (see Supplementary Table S1 for a detailed description of them) and Boolean operators were used to generate the following search string:

((Antioxidants) OR (Ascorbic Acid) OR (Vitamin E) OR (acetylcysteine) OR (Glutathione) OR (Thioctic Acid) OR (Selenium)) AND ((Pulmonary Disease, Chronic Obstructive) OR (COPD) OR (Chronic Obstructive Lung Disease)) AND ((Therapeutics) OR (Clinical Trial) OR (Nursing Care)) AND ((Randomized Controlled Trial) OR (Controlled Clinical Trial) OR (Clinical Trial)).

For each database, it was necessary to vary how the different terms were written, while ensuring that the search string remained constant. An “advanced search” was performed in each database (Supplementary Table S2).

### 2.2. Eligibility Criteria

Articles that met the following criteria were included in the study:

1. Original studies or clinical trials that evaluated oxidative stress in patients with COPD.
2. Published in English or Spanish between January 2020 and December 2025.
3. Studies that analyzed molecular mechanisms, oxidative biomarkers, or antioxidant interventions.
4. Full-text availability and a clearly described methodology.

Exclusion criteria were as follows:

1. Studies conducted exclusively in animal or cell models without clinical correlation.
2. Research focused on other respiratory diseases without differentiation from COPD.
3. Duplicate articles, conference abstracts, or brief communications without quantitative data.
4. Observational studies without intervention.
5. Case–control studies.
6. Cohort studies.
7. Systematic reviews.
8. Studies lacking a comparator group.

### 2.3. Definition of Antioxidant Interventions

The intervention component of the PICO framework was defined as any strategy with demonstrated or hypothesized antioxidant or redox-modulating effects. This inclusive definition was adopted to minimize selection bias and to reflect the multifactorial nature of OS in COPD.

Eligible interventions included: (i) pharmacological agents with established antioxidant properties (e.g., N-acetylcysteine); (ii) nutraceuticals and plant-derived compounds with documented antioxidant activity (e.g., polyphenols, carotenoids, and herbal extracts); (iii) metabolic interventions influencing redox homeostasis (e.g., iron supplementation), and (iv) non-pharmacological strategies such as structured exercise programs, which have been shown to enhance endogenous antioxidant defenses.

Studies were included based on predefined methodological criteria (randomized controlled design and outcome reporting), rather than on the specific type of compound, ensuring a criteria-driven rather than compound-driven selection process.

### 2.4. Selection of Articles

Two reviewers selected articles through a double-blind review process that included evaluation of titles, abstracts, and full texts. Discrepancies were resolved by consensus or by a third reviewer.

The selection process was documented in a PRISMA flow diagram, which shows the number of studies identified, excluded, and finally those included in the qualitative synthesis.

### 2.5. Data Extraction and Synthesis

The same two reviewers read the whole selected articles and extracted the following data from each article:

1. Author and year of publication.
2. Type and design of the study.
3. Population or sample analyzed.
4. Intervention.
5. Main findings and conclusions.
6. Quality of the study.

### 2.6. Methodological Quality Assessment

The internal quality of each article was evaluated by means of the Critical Appraisal Skills Programme (CASP) for systematic reviews. (Supplementary Table S3), which consists of a series of questions that firstly analyze the internal validity of the study in terms of methodological adequacy and correctness (“Are the results of the study valid?”); secondly, it identifies the results of the research (“What are the results?”); and thirdly, it analyses whether the results obtained in the studies can be extrapolated to the patient in question (“Will the results be useful for treating my patients?”). Studies were classified as high (≥9), moderate (7–8), or low (<7) methodological quality [10]. CASP scores are reported in Table 1.

### 2.7. Statistical Analysis and Effect Size Measures

A meta-analysis was performed on Randomized Clinical Trials (RCTs) with comparable outcomes. In all cases, data were extracted or calculated from the data in the publications. Due to the high methodological heterogeneity of the studies (differences in the type of intervention, duration, and severity of COPD), the random effects model (DerSimonian-Lair method) was used to perform the meta-analyses. Heterogeneity between studies was assessed using Cochran’s Q statistic and quantified using the I^2^ statistic, with values of 25%, 50%, and 75% representing low, moderate, and high heterogeneity, respectively. Forest plots were generated to visually display individual and pooled effect estimates. For exacerbation risk calculations, pooled effects were expressed as risk ratio (RR) with 95% confidence interval (95% CI).

### 2.8. Protocol and Registration

The study protocol was prospectively registered in the Open Science Framework (OSF) (DOI: 10.17605/OSF.IO/WKZAX).

## 3. Results

### 3.1. Description of Search Results

**Figure 1:**
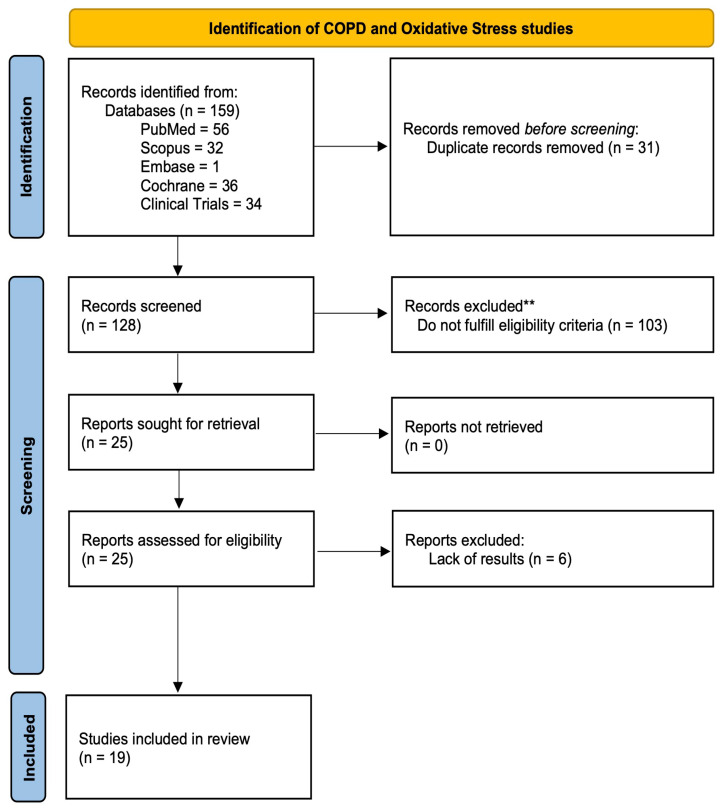
PRISMA 2020 flow diagram indicating the search and selection of articles used in the study [9]. ** No automation tools were used. Excluded records were excluded by a human based on the reading of the title and abstract of each article.

The flowchart below shows the process of searching for and selecting the scientific articles used to carry out the study (Figure 1).

Using the search string described above, a total of 159 articles were identified across the following databases: PubMed (n = 56), Scopus (n = 32), Embase (n = 1), Cochrane (n = 36), and Clinical Trials (n = 34).

In the screening phase, duplicate articles (n = 31) were eliminated, and upon reviewing the titles and abstracts of the remaining articles (n = 128), 100 were discarded as they did not meet the eligibility criteria. After this first screening phase, the number of articles decreased from 159 to 25.

These 25 articles, suitable for the study, underwent the text filter, which involved reading the full texts and verifying that they contained the sought information. After reading each of the articles, six were removed, mainly due to a lack of conclusive results. Finally, 19 articles were selected for the present study.

### 3.2. Study Selection

A total of 19 studies that met the eligibility criteria were included in the analysis (Table 1; IDs: 1–19). All studies were RCTs, and no observational, case–control, or cohort studies, or systematic reviews, were included.

Table 1 presents the information regarding the studies analyzed. It includes information about the article itself (authors, year of publication, and PubMed identifier (PMID)), the characteristics of the studies, the intervention performed, the results obtained after the intervention, and the methodological quality of each article. In addition, Supplementary Tables S4 and S5 present the analysis tables independently prepared by the two reviewers. These two tables are summarized in Table 1.

### 3.3. General Characteristics of the Articles Analyzed

The articles were published between January 2020 and December 2025 and included patients with mild to moderate COPD, with some studies including patients from moderate to severe disease (IDs: 7, 9). Sample sizes ranged from 20 participants in intervention studies to 924 patients in a large-scale multicenter RCT (ID: 7). Most studies were double-blind, placebo-controlled. Methodological quality assessment using the CASP tool showed high quality (≥10/11) in 14 studies (74%) and moderate quality (8–9/11) in the remaining 5 studies (26%).

### 3.4. Classification of Interventions and Outcomes

The included studies evaluated a wide range of interventions with potential effects on OS in COPD patients, which could be grouped into five main categories: (i) thiol antioxidants (NAC and combinations with propolis; IDs 1, 7, and 9); (ii) phytonutrients and natural bioactive compounds (*Zataria multiflora*, *Crocus sativus*, *Nigella sativa*, *Withania somnifera*; IDs 2, 5, 6, 17, 18, and 19); (iii) metabolic supplements with redox impact (beta-alanine, iron, resveratrol, and dietary nitrates; IDs 4, 8, 15, and 11); (iv) exercise- or rehabilitation-based interventions (IDs 3, 12, 13, and 14); and (v) specific respiratory strategies (inhaled nitric oxide and IMT + NPPV; IDs 10 and 16).

The outcomes were classified into four groups to determine the effect of antioxidant therapies on aspects related to clinical and biological aspects of COPD as follows: (i) OS and systemic inflammation; (ii) exacerbations; (iii) respiratory function tests, and (iv) exercise capacity.

#### 3.4.1. Oxidative Stress and Systemic Inflammation Outcomes

The most widely evaluated outcome was OS and systemic inflammation, with data available in 12 of the 19 trials (IDs 2, 3, 5, 6, 8, 11, 13, 14, 15, 16, 17, and 18). Most interventions improved REDOX balance, as reflected by reductions in markers of oxidative damage or increases in total antioxidant capacity. These effects were observed in interventions with plant antioxidants (*Zataria multiflora* [ID 2], crocin [IDs 5, 6], *Nigella sativa* [ID 17], *Withania somnifera* [ID 18]), iron (ID 11), and structured exercise programs (IDs 3, 13, and 14). The combination of inspiratory training with non-invasive ventilation (ID 16) was also associated with a reduction in OS mediated by inflammatory signaling pathways. Several studies analyzed specific systemic inflammatory markers linked to OS. Reductions in IL-6, TNF-alpha, NF-κB, and C-reactive protein were reported following antioxidant therapy (ID 5, 6, 16, 18).

In contrast, beta-alanine (ID 8) and resveratrol (ID 15) did not reduce REDOX biomarkers.

#### 3.4.2. Exacerbations Outcomes

Exacerbation events were reported in trials with NAC or NAC + propolis (IDs 1, 7, and 9). Two studies (IDs 1 and 9) reported a significant reduction in exacerbations, while the largest trial with high methodological quality using NAC monotherapy (ID 7) found no significant clinical benefit.

#### 3.4.3. Pulmonary Function Test Outcomes

Pulmonary function tests (PFTs) were evaluated in eight studies (IDs 2, 5, 6, 7, 9, 13, 17, and 19). Improvements in spirometry were observed with phytonutrient-based interventions, particularly *Zataria multiflora* (IDs 2 and 19), *Crocus sativus* (IDs 5 and 6), and *Nigella sativa* (ID 17). Structured exercise (ID 13) was also associated with favorable changes in functional parameters. In contrast, NAC (ID 7) did not demonstrate significant improvements in PFTs.

#### 3.4.4. Exercise Capacity Outcomes

Exercise capacity was assessed in nine studies (IDs 3, 4, 5, 6, 8, 10, 12, 14, and 16). The most consistent improvements were observed when the intervention included a direct physiological stimulus, such as physical training or pulmonary rehabilitation. The eccentric versus concentric training (ID 3), the pulmonary rehabilitation combined with melatonin (ID 12), and the resistance + HIIT program (ID 14) demonstrated clear improvements in functional performance. Dietary nitrate (ID 4) and inhaled nitric oxide (ID 10) have been shown to enhance ventilatory efficiency during exercise, while the combination of IMT with NPPV (ID 16) has demonstrated an increased exercise tolerance. In contrast, beta-alanine (ID 8) did not produce significant functional benefits.

### 3.5. Meta-Analysis

#### Meta-Analysis of Exacerbations

**Figure 2:**
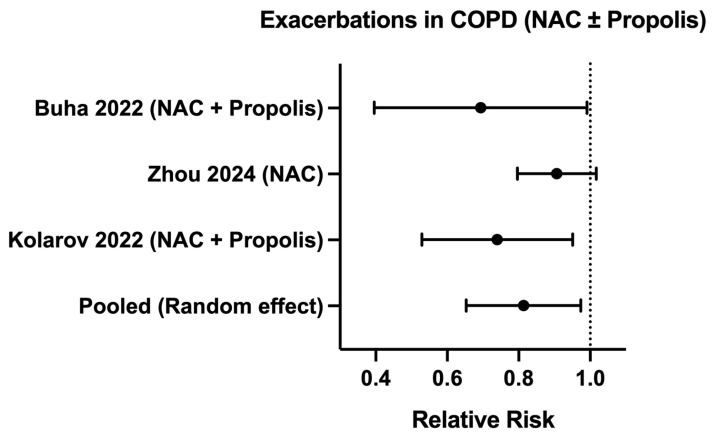
Forest plot showing the random-effects meta-analysis of RCTs evaluating NAC-based interventions versus placebo on the risk of COPD exacerbations [11,17,19]. Effect estimates are presented as relative risk ratios with 95% confidence intervals. The pooled estimate indicates a significant reduction in exacerbation risk in NAC-based interventions (RR 0.80; 95% CI 0.66–0.98). The vertical line represents no effect (RR = 1).

Three RCTs (IDs 1, 7 and 9) assessed the effect of NAC treatment, either alone or in combination with propolis, on the risk exacerbation in COPD patients. The three studies included a total of 1086 individuals. The results show a statistically significant reduction in exacerbation risk (RR: 0.80; 95% CI: 0.66–0.98) in patients receiving NAC-based therapies compared with placebo (Figure 2; Supplementary Table S6). Moderate heterogeneity was observed (I^2^: 43.8%), indicating differences in treatment (3–24 months), therapies (NAC alone vs. combined with propolis) and differences in COPD severity (moderate vs. severe).

## 4. Discussion

COPD is a progressive respiratory disease characterized by chronic airway inflammation, fibrosis of the small airways, and destruction of the lung parenchyma (emphysema), leading to airflow limitation. It is the fourth leading cause of death worldwide.

Over the years, a body of scientific evidence has accumulated showing that OS plays a role in the pathophysiology of the disease [30]. Patients with COPD are chronically exposed to exogenous oxidants (caused by cigarette and/or biomass smoke and air pollution), as well as to endogenous oxidants generated by inflammatory and structural cells in the lungs. These ROS cause oxidative damage and maintain active inflammatory pathways even after the exposure to the primary risk factor has ceased. Simultaneously, there is a reduction in endogenous antioxidant systems, including dysfunction of key enzymes (superoxide dismutase, catalase, and glutathione peroxidase), and regulatory transcription factors (nuclear factor erythroid 2-related factor 2 (Nrf2) and forkhead box O (FOXO)), which weakens the defense against OS. Redox imbalance increases chronic inflammation and contributes to the development of lung fibrosis, corticosteroid resistance, accelerated pulmonary aging, DNA damage, and autoantibody formation [3].

Based on this information, it has been proposed that treating OS, either by providing exogenous antioxidants or by enhancing endogenous antioxidant systems, could be an effective therapeutic strategy [31]. Despite the biological rationale, current antioxidant therapies have shown limited clinical efficacy, indicating the need for more effective strategies. This discrepancy between the biological evidence and the observed clinical results supports the need to systematically evaluate the existing evidence to identify which interventions could be beneficial. In this context, it is important to conduct a systematic review focused exclusively on RCTs, as these constitute the gold standard for establishing causal relationships between interventions and clinical outcomes, reducing the risk of bias inherent in other study designs, and ensuring that the conclusions drawn from the review are based on the best available evidence to inform future therapeutic strategies. An important methodological aspect of this review is the broad inclusion of redox-modulating interventions beyond conventional pharmacological agents. This approach reflects the complex and multifactorial nature of oxidative stress in COPD, where both exogenous and endogenous mechanisms contribute to disease progression. Accordingly, interventions such as nutraceutical compounds, plant-derived antioxidants, and structured exercise programs were included, as all have demonstrated the ability to modulate OS pathways and antioxidant defenses in clinical or experimental settings.

Nineteen RCTs were included in the systematic review. Outcomes were categorized into four groups: (i) OS and systemic inflammation, (ii) exacerbations, (iii) pulmonary function tests, and iv) exercise capacity. The heterogeneity of these approaches allowed for the evaluation of OS modulation from pharmacological, nutritional, and exercise perspectives.

Regarding OS and systemic inflammation, most interventions showed favorable effects on the REDOX balance, evidenced by reductions in markers of oxidative damage or increases in markers of antioxidant capacity. Significant reductions in OS markers were observed following treatments with plant antioxidants [12,15,16,27,28], iron supplementation [21], and exercise-based interventions [13,23,24]. The combination of inspiratory muscle training with non-invasive ventilation [26] reduced OS and regulated inflammatory signaling pathways. Over the years, scientific evidence indicates that a significant reduction in OS markers is achieved through complementary mechanisms. Plant antioxidants act directly and indirectly, neutralizing free radicals and stimulating protective endogenous enzymes [32,33,34]. Iron supplementation optimizes mitochondrial function by preventing electron leakage that generates ROS [35]. Finally, physical exercise induces an adaptive response called hormesis, in which the moderate stress of activity strengthens the body’s defense systems and promotes mitochondrial biogenesis [36].

In contrast, beta-alanine [18] and resveratrol [25] did not reduce OS biomarkers. Although both beta-alanine and resveratrol showed promising results in preclinical models, clinical trials revealed that neither compound produced consistent changes in OS or inflammatory biomarkers [25,37,38,39,40]. Moreover, several studies have demonstrated reductions in systemic inflammatory markers (e.g., IL-6, TNF-α, and CRP) following antioxidant interventions. These findings support the concept of a linked oxidative stress–inflammation axis in COPD, so that reducing OS levels may modulate inflammatory signaling. However, heterogeneity in inflammatory outcomes and measurement techniques limits definitive conclusions. Future research should integrate standardized inflammatory and redox biomarkers to clarify mechanistic relationships.

Exacerbation outcomes were primarily reported in trials evaluating NAC-based interventions [11,17,19]. In our review, two studies combining NAC with propolis showed a significant reduction in exacerbations [11,19], whereas a large CT using NAC monotherapy did not significantly reduce exacerbations in patients with mild-to-moderate COPD [17]. Thiol-based antioxidants, particularly N-acetylcysteine (NAC), have been widely used in the treatment of COPD [41,42]. NAC is a ROS scavenger and a precursor of Glutathione (GSH), the most abundant intracellular antioxidant [43]. Current evidence supports the use of NAC in COPD patients, as long-term use is well tolerated and a reduction in exacerbation frequency has been reported in patients with chronic bronchitis and moderate to severe COPD [44,45,46,47]. However, the BRONCUS (Bronchitis Randomized on NAC Cost-Utility Study) study showed that N-acetylcysteine (600 mg/day) is not effective in preventing lung function decline or reducing exacerbations in COPD patients during three years of follow-up [48]. These discrepancies may be explained by differences in dosage, treatment duration, baseline exacerbation risk, concomitant therapies, and patient phenotype (e.g., frequent versus infrequent exacerbators) across the studies, which may influence NAC response. In addition, the benefits observed with NAC combined with propolis suggest the possibility of synergistic antioxidant and/or anti-inflammatory effects, which deserve further investigation in larger clinical trials.

Pulmonary function tests (PFTs) were evaluated in eight studies. Improvements in PFT (mainly in spirometric parameters) were observed in studies evaluating plant intervention [12,15,16,27,29] and structured exercise training [23]. Structured exercise programs that include aerobic and inspiratory muscle training have been shown to significantly improve exercise capacity, respiratory muscle strength, and spirometric values in patients with COPD [49]. These improvements are due to changes in the skeletal and respiratory muscles, reduced dyspnea, and improved gas exchange [50,51]. In contrast, NAC in monotherapy did not show significant improvement in PFT [17,52]. However, in PFTs, the magnitude of the changes was small, suggesting that conventional spirometry may be relatively insensitive to short- or medium-term modulation of OS and therefore not the best method for measuring changes. It raises the need for alternative outcome measures, such as imaging or biochemical biomarkers, which may better identify small physiological improvements.

Exercise capacity outcomes were evaluated in nine studies. The most consistent improvements were observed when interventions incorporated a direct physiological stimulus, such as exercise training or pulmonary rehabilitation. Significant gains in functional capacity were observed with eccentric versus concentric training [13], pulmonary rehabilitation combined with melatonin [22], and resistance plus high-intensity interval training [24]. Dietary nitrate supplementation [14] and inhaled nitric oxide [20] improved ventilatory efficiency during exercise, whereas inspiratory muscle training combined with non-invasive ventilation [26] increased exercise tolerance. However, antioxidant supplementation alone is insufficient. Beta-alanine supplementation [18] did not improve performance, suggesting that supplements alone are not effective in improving functional capacity in COPD patients without associated physical training.

In the present study, we performed a meta-analysis of those outcomes that showed less heterogeneity between studies (exacerbations). Our results indicate that in COPD, interventions aimed at modulating the REDOX balance are associated with improvements in functional performance and a reduction in the risk of exacerbations. The meta-analysis of exacerbations showed a 20% reduction in the RR of exacerbations in patients who received NAC-based therapies compared with placebo, suggesting that this antioxidant strategy may be acting on the inflammatory and oxidative pathways involved in the development of exacerbations. However, while the meta-analysis provides quantitative support for a reduction in exacerbation risk, the pooled estimate was based on a limited number of RCTs, and although heterogeneity was moderate, the confidence interval approached the null effect. This suggests that the magnitude of benefit, while statistically significant, remains modest. In addition, the small number of included studies limits the robustness of between-study variance estimation and the pooled effect may be influenced by individual studies, particularly those with larger sample sizes. In any case, no study reported adverse effects from the use of antioxidants, and in all cases, benefits were shown. Therefore, these findings should be considered hypothesis-generating and supportive rather than definitive, highlighting the need for larger, well-designed RCTs to confirm the observed effects.

As mentioned above, the role of NAC on COPD exacerbations has been widely studied. NAC increases levels of intracellular glutathione, reduces ROS generation and modulates the NF-kB mediated inflammatory signaling, thereby targeting important pathways involved in exacerbation. Clinical evidence supports the NAC role in reducing exacerbation frequency. Beyond the BRONCHUS study, the PANTHEON trial included a larger number of patients showed a significant reduction in annual exacerbation rates in patients with moderate to severe COPD [53]. Similarly, a meta-analysis reported an RR reduction from 15–25% in a subgroup of patients not receiving corticosteroid treatment or with chronic bronchitis [54,55]. These findings are consistent with meta-analysis results since a significant reduction in exacerbation rate (RR: 0.8) after NAC treatment is observed, supporting the hypothesis that OS modulation may be a clinically relevant therapeutic target in COPD.

In view of all these results, the following question arises: if OS is involved in the pathophysiology of COPD, why is there currently no antioxidant therapy being used in clinical practice? This question has been and continues to be the subject of debate and has been extensively reviewed by Barnes [3]. The available data indicate that there is indeed a scientific basis for the use of antioxidant therapies for the treatment of COPD. The main problem with current treatments (bronchodilators, steroids) lies in the fact that antioxidants may not be addressing OS, so new therapies should be aimed not only at alleviating symptoms but also at specifically treating those pathways involved in REDOX signaling that are affected in each disease (in our case, COPD). The possible explanation for why current antioxidants show limited results is possibly because they are not acting on the specific therapeutic target, in addition to their possible inactivation in the pulmonary environment where there are high OS and limitations to their delivery to the lungs. Taken together, these results indicate that it is necessary to continue deepening our understanding of OS in the pathophysiology of COPD, which will lead to the discovery of new REDOX therapeutic targets and will also allow identify OS-based biomarkers and identify those patients who may benefit from therapies aimed at modifying and restoring the REDOX balance, the efficacy of which must be validated through robust RCTs.

A major strength of this systematic review is its exclusive focus on RCTs, which minimizes bias and strengthens causal inference. The broad range of interventions analyzed also provides a comprehensive overview of REDOX-targeted strategies in COPD. However, several limitations must also be acknowledged. An important consideration in interpreting the present findings is the heterogeneity of the included interventions. Although all strategies were selected based on their antioxidant or redox-modulating properties, they include a wide range of approaches, including pharmacological agents, nutraceutical and plant-derived compounds, metabolic interventions, and structured exercise programs. These interventions differ not only in their mechanisms of action but also in their primary clinical targets. For instance, thiol-based agents such as N-acetylcysteine primarily act by replenishing intracellular glutathione, whereas plant-derived compounds may exert pleiotropic effects through polyphenolic pathways. In contrast, exercise-based interventions enhance endogenous antioxidant defenses and mitochondrial function. To address this heterogeneity, results must be interpreted with caution, and the overall findings should not be considered as representing a single homogeneous antioxidant strategy, but rather as a collective signal supporting the relevance of OS modulation in COPD. We also acknowledge that other pharmacological molecules, showing antioxidant activity (e.g., erdosteine), were not included. This reflects the absence of eligible RCTs meeting our predefined inclusion criteria and outcome requirements at the time of the literature search, rather than an *a priori* exclusion. Many trials were relatively small, which limited statistical power and generalizability. There was also substantial heterogeneity in interventions, outcomes, and biomarker assessment. Follow-up durations were often short, preventing assessment of long-term clinical impact.

## 5. Conclusions

This review shows that OS is a biological target in COPD. Currently, OS-targeted therapies should be considered complementary, rather than separate treatments for COPD. NAC-based therapies may be considered for selected patients, especially those with frequent exacerbations; however, evidence remains inconclusive. Although phytochemical and antioxidant supplementation appears biologically beneficial, it requires further validation before routine clinical implementation. Most importantly, interventions combining REDOX modulation with exercise provide the most consistent clinical benefits and align with the best practices for managing COPD. While thiol-based antioxidants show potential in reducing exacerbations in certain groups, combined and multimodal strategies offer the most significant improvements in functional outcomes.

In summary, the results of this study indicate that antioxidant therapies may represent complementary therapeutic strategies to standard therapies for the treatment of COPD; however, the low number of patients included in some of the RCTs analyzed, the short duration of the studies, and the heterogeneity between studies indicate that the results should be interpreted with caution and point to the need for more robust RCTs to obtain conclusive results.

## Figures and Tables

**Table 1:** Summary of articles included in the systematic review.

Study ID	1st Author (Year) PMID	Study Design	Intervention	Comparator	Sample Size N; I/C	Duration	Primary Outcomes	Study Conclusions	CASP Score
1	Buha I (2022) [11] PMID:35856373	RCT, DB, PC	NAC + Propolis	Placebo	N = 46; 27/19	3 months	Exacerbations	NAC significantly reduced exacerbation frequency.	11/11
2	Ghorani V (2020) [12] PMID: 32202330	RCT, DB, PC	*Zataria multifora* extract	Placebo	N = 42; 29/13	2 months	Symptoms, PFT; OS parameters; CRP	Z. multiflora improved the clinical symptoms and PFT, while reducing OS and CRP of COPD patients.	9/11
3	Valero M (2023) [13] PMID: 36839267	RCP, PC	Eccentric (ECC) and Concentric (CONC) cyclic training	ECC vs. CONC	N = 20; 10/10	12 weeks	VO2 peak and PO max, TTE. Plasma antioxidant and oxidative markers, insulin resistance, lipid profile, systemic inflammation markers	CONC training improved insulin sensitivity, increased antioxidant capacity at rest, and reduced exercise-induced OS in moderate COPD patients.	9/11
4	Pavitt MJ (2022) [14] PMID:34853156	RCT, DB, PC, CO	Dietary nitrate	Placebo	N = 20; 10/10	Single dose	Exercise capacity	Dietary nitrate improved exercise efficiency in hypoxic COPD patients	10/11
5	Ghodabi H (2022) [15] PMID: 35517806	RCT, DB, PC	*Crocus sativus* extract (Crocin)	Placebo	N = 46; 23/23	12 weeks	PFT, 6MWD, TAOC, NF-KB	Crocin reduces OS and improves exercise capacity.	10/11
6	Aslani M (2023) [16] PMID: 36628554	RCT, DB, PC	*Crocus sativus* extract (Crocin)	Placebo	N = 57; 28/29	12 weeks	IL-6, TNF-α, exercise capacity, PFT	Crocin significantly reduced inflammatory markers and increased exercise capacity.	11/11
7	Zhou Y (2024) [17] PMID: 39349461	RCT, DB, PC	NAC	Placebo	N = 924; 464/460	24 months	PFT, exacerbations	Long-term treatment with high-dose NAC did not significantly reduce exacerbations or improve PFT in patients with mild-to-moderate COPD.	10/11
8	Brandt J (2022) [18] PMID: 35977911	RCT, DB, PC	Beta-alanine	Placebo	N = 40 21/19	12 weeks	Physical capacity, muscle strength, OS	No significant functional benefit or OS improvement observed.	10/11
9	Kolarov V (2022) [19] PMID: 35587070	RCT, DB, PC	NAC + Propolis (NACp)	Placebo	N = 116 74/42	3 and 6 months	QoL, exacerbations, PFT, exacerbations	NACp reduces exacerbations and improves QoL	10/11
10	Phillips D (2021) [20] PMID: 33428233	RCT, PC	iNO	Placebo	N = 30 15/15	—	Cardiopulmonary exercise tests. Ventilatory efficiency.	iNO enhances maximum oxygen uptake. Acute improvement in ventilatory efficiency.	11/11
11	Pérez M (2021) [21] PMID: 34572377	RCT, PC	Iron therapy	Placebo	N = 66 44/22	15 min	Redox balance	Improved systemic redox balance.	11/11
12	Viana S (2023) [22] PMID: 37944829	RCT, PC	Melatonin + PR	PR alone	N = 39 18/21	12 weeks	6MWD, health status, QoL	Melatonin enhances exercise capacity, health status, and QoL.	10/11
13	Domaszewska K (2022) [23] PMID: 35565914	RCT	Exercise training	Control	N = 32 20/12	2 h	PFT, OS	Exercise did not increase plasma OS or total phenolics, but it did improve PFTs.	8/11
14	Baltasar I (2023) [24] PMID: 37322570	RCT	Resistance + HIIT	Control	N = 21 8/13	12 weeks	QoL, Muscle dysfunction, Oxidative damage	Training improved physical function and QoL and reduced systemic oxidative damage.	10/11
15	Beijers R (2020) [25] PMID: 31996311	RCT, PC	Resveratrol	Placebo	N = 21 11/10	4 weeks	Metabolic and OS markers	No significant effects on oxidative or functional outcomes.	9/11
16	Lei Y (2023) [26] PMID: 37828534	RCT	IMT + NPPV	IMT	N = 100 50/50	8 weeks	Exercise capacity, QoL, OS	Combined therapy reduced OS and increased exercise capacity and QoL.	10/11
17	Al-Azzawi M (2020) [27] PMID: 32904114	RCT, DB, PC	*Nigella sativa* extract (Black seed oil)	Placebo	N = 91 47/44	3 months	PFT, OS, inflammatory markers	Improved pulmonary function and oxidative and inflammatory status.	11/11
18	Singh P (2022) [28] PMID: 35784687	RCT, DB, PC	*Withania somnifera* extract	Placebo	N = 150 50/50/50	12 weeks	QoL, inflammation, OS	Improved quality of life and reduced inflammation and OS.	10/11
19	Ghorani V (2024) [29] PMID: 38373666	RCT, DB, PC	*Zataria multiflora* extract	Placebo	N = 45 14/31	2 months	Symptoms, PFT	Improved respiratory symptoms and spirometry.	8/11

## Data Availability

The original contributions presented in this study are included in the article/Supplementary Materials. Further inquiries can be directed to the corresponding author.

## Supplementary Materials

The following supporting information can be downloaded at: https://www.mdpi.com/article/10.3390/antiox15040446/s1, Table S1: MeSH and non-MeSH terms used to carry out the bibliographic search; Table S2: Search terms adjusted to each database; Table S3: CASP Table; Table S4: Articles reviewed by reviewer #1; Table S5: Articles reviewed by reviewer #2. Table S6: Meta-analysis of exacerbations in COPD. References [56,57] are cited in the Supplementary Materials.

## Abbreviations

The following abbreviations are used in this manuscript:

COPD	Chronic Obstructive Pulmonary Disease
OS	Oxidative Stress
ROS	Reactive Oxygen Species
PRISMA	Preferred Reporting Items for Systematic Reviews and Meta-analysis
CASP	Critical Appraisal Skills Programme
RCT	Randomized Clinical Trial
PMID	PubMed Identifier
IL-6	Interleukin 6
ID	Identification number
