# Supplementary material for: Antioxidants as Therapeutic Tools in the Management of COPD: A Systematic Review with Meta-Analysis
Source: Antioxidants (Basel). 2026 Apr 2;15(4):446. doi: 10.3390/antiox15040446 (PMC13113252; doi:10.3390/antiox15040446)
Supplement: Supplementary file 1 [file antioxidants-15-00446-s001.zip › Supplementary Table S1.pdf]

Supplementary Table S1. MeSH and non-MeSH terms used to carry out the bibliographic search.

Term	Definition and MeSH ID
Antioxidants	Natural or synthetic substances that inhibit or delay oxidation reactions. They counteract the harmful effects of oxidation on animal tissues.<br>MeSH Unique ID: D000975
Ascorbic Acid	A six-carbon compound related to glucose. It is found naturally in citrus fruits and many vegetables. Ascorbic acid is an essential nutrient in the human diet and is necessary for the maintenance of connective tissue and bones. Its biologically active form, vitamin C, functions as a reducing agent and coenzyme in various metabolic pathways. Vitamin C is considered an antioxidant.<br>MeSH Unique ID: D001205
Vitamin E	A generic descriptor for all tocopherols and tocotrienols that exhibit alpha-tocopherol activity. Thanks to the phenolic hydrogen in the 2H-1-benzopyran-6-ol nucleus, these compounds exhibit varying degrees of antioxidant activity, depending on the location and number of methyl groups, as well as the type of isoprenoids.<br>MeSH Unique ID: D014810
Acetylcysteine	The N-acetylated derivative of cysteine. It is used as a mucolytic to reduce the viscosity of mucous secretions. It has also been shown to have antiviral effects in HIV patients due to the inhibition of viral stimulation by reactive oxygen species.<br>MeSH Unique ID: D000111
Glutathione	A tripeptide with multiple functions in cells. It conjugates with drugs to increase their solubility for excretion, serves as a cofactor for some enzymes, participates in the reorganization of protein disulfide bonds, and reduces peroxides.<br>MeSH Unique ID: D005978
Thiolic Acid<br>Thioctic Acid	An octanoic acid with two sulfur bridges, which is why it is sometimes also referred to as pentanoic acid in some nomenclature schemes. It is biosynthesized by cleavage of linoleic acid and is a coenzyme of oxoglutarate dehydrogenase (ketoglutarate dehydrogenase complex). It is used in dietary supplements.<br>MeSH Unique ID: D008063
Selenium	Element with the atomic symbol Se, atomic number 34, and atomic weight 78.97. It is an essential micronutrient for mammals and other animals, but is toxic in large amounts. Selenium protects intracellular structures from oxidative damage. It is an integral component of glutathione peroxidase.<br>MeSH Unique ID: D012643
Pulmonary Disease, Chronic Obstructive	Chronic, diffuse, and irreversible airflow obstruction disease. COPD is subclassified as chronic bronchitis and pulmonary emphysema.<br>MeSH Unique ID: D029424
COPD	Synonym for "Pulmonary Disease, Chronic Obstructive". Non-MeSH term.
Chronic Obstructive Lung Disease	Synonym for "Pulmonary Disease, Chronic Obstructive". Non-MeSH term.
Therapeutics	Procedures related to the curative treatment or prevention of diseases.<br>MeSH Unique ID: D013812
Clinical Trial	A study that reports the results of a clinical trial in which participants are assigned to one or more interventions so that researchers can evaluate them in terms of biomedical or health outcomes. Assignments are determined according to the study protocol. Participants may receive diagnostic, therapeutic, or other interventions. For clinical trials involving veterinary animals, see VETERINARY CLINICAL TRIAL. The term Clinical Trials was used for both humans and nonhumans before 2019.<br>MeSH Unique ID: D016430
Nursing Care	Care provided to patients by nursing staff.<br>MeSH Unique ID: D009732
Randomized Controlled Trial	A paper reporting a clinical trial involving at least one test treatment and one control treatment, simultaneous enrollment and follow-up of the groups treated with the test and control treatments, and in which a random process, such as a random number table, selects the treatments to be administered.<br>MeSH Unique ID: D016449
Controlled Clinical Trial	Work reporting on a clinical trial that includes one or more test treatments, at least one control treatment, specific outcome measures to evaluate the intervention under study, and a bias-free method for assigning patients to the test treatment. The treatment may consist of drugs, devices, or procedures studied for their diagnostic, therapeutic, or prophylactic efficacy. Control measures include placebos, active medications, no treatment, pharmaceutical forms and regimens, historical comparisons, etc. When randomization using mathematical techniques, such as the use of a random number table, is used to assign patients to test or control treatments, the trial is characterized as a randomized controlled trial.<br>MeSH Unique ID: D018848
