# Supplementary material for: Antioxidants as Therapeutic Tools in the Management of COPD: A Systematic Review with Meta-Analysis
Source: Antioxidants (Basel). 2026 Apr 2;15(4):446. doi: 10.3390/antiox15040446 (PMC13113252; doi:10.3390/antiox15040446)
Supplement: Supplementary file 1 [file antioxidants-15-00446-s001.zip › Supplementary Table S2.pdf]

PubMed literature search

**DATABASE:** PubMed	
**ADVANCED SEARCH STRATEGY**: ((Antioxidants) OR (Ascorbic Acid) OR (Vitamin E) OR (acetylcysteine) OR (Glutathione) OR (Thioctic Acid) OR (Selenium)) AND ((Pulmonary Disease, Chronic Obstructive) OR (COPD) OR (Chronic Obstructive Lung Disease)) AND ((Therapeutics) OR (Clinical Trial) OR (Nursing Care)) AND ((Randomized Controlled Trial) OR (Controlled Clinical Trial) OR (Clinical Trial))	
**FILTERS USED**: Year: 2020-2025. Whole text.	**NUMBER OF DOCUMENTS FOUND**: 53

Scopus literature search

**DATABASE:** Scopus	
**ADVANCED SEARCH STRATEGY**: TITLE-ABS-KEY (("Antioxidants") OR ("Ascorbic Acid") OR ("Vitamin E") OR ("acetylcysteine") OR ("Glutathione") OR ("Thioctic Acid") OR ("Selenium")) AND (("Pulmonary Disease, Chronic Obstructive") OR ("COPD") OR ("Chronic Obstructive Lung Disease")) AND (("Therapeutics") OR ("Clinical Trial") OR ("Nursing Care")) AND (("Randomized Controlled Trial") OR ("Clinical Trial") OR ("Controlled Clinical Trial")) AND PUBYEAR > 2020 AND PUBYEAR < 2026 AND (LIMIT-TO (SUBJAREA , "NURS")) AND (LIMIT-TO (EXACTKEYWORD , "Controlled Study"))	
**FILTERS USED**: Year: 2020-2025. Subject area: Nursing**.** Keyword: Controlled study	**NUMBER OF DOCUMENTS FOUND**: 32

EMBASE literature search

**DATABASE:** EMBASE	
**ADVANCED SEARCH STRATEGY**: ('antioxidant'/exp OR 'antioxidant' OR 'ascorbic acid'/exp OR 'ascorbic acid' OR 'vitamin e therapeutic use'/exp OR 'vitamin e therapeutic use' OR 'acetylcysteine'/exp OR 'acetylcysteine' OR 'glutathione'/exp OR 'glutathione' OR 'thioctic acid'/exp OR 'thioctic acid' OR 'selenium'/exp OR 'selenium') AND 'chronic obstructive lung disease':ti,ab,kw AND ('therapy':ti,ab,kw OR 'clinical trial':ti,ab,kw OR 'nursing care':ti,ab,kw) AND ('randomized controlled trial':ti,ab,kw OR 'controlled study':ti,ab,kw OR 'clinical trial':ti,ab,kw)	
**FILTERS USED**: None	**NUMBER OF DOCUMENTS FOUND**: 1

Cochrane literature search

**DATABASE:** Cochrane	
**ADVANCED SEARCH STRATEGY**: ("Antioxidants" OR "Ascorbic Acid" OR "Vitamin E" OR "acetylcysteine" OR "Glutathione" OR "Thioctic Acid" OR "Selenium") AND ("COPD" OR "Chronic Obstructive Lung Disease" OR "Pulmonary Disease, Chronic Obstructive") AND ("Therapeutics" OR "Clinical Trial" OR "Nursing Care") AND ("Randomized Controlled Trial" OR "Clinical Trial" OR "Controlled Clinical Trial")	
**FILTERS USED**: Publication Year: 2020-2025	**NUMBER OF DOCUMENTS FOUND**: 36

Clinical Trials literature serach

**DATABASE:** Clinical Trials	
**ADVANCED SEARCH STRATEGY**:<br>("Antioxidants" OR "Ascorbic Acid" OR "Vitamin E" OR "acetylcysteine" OR "Glutathione" OR "Thioctic Acid" OR "Selenium") AND ("COPD" OR "Chronic Obstructive Lung Disease" OR "Pulmonary Disease, Chronic Obstructive") AND ("Therapeutics" OR "Clinical Trial" OR "Nursing Care") AND ("Randomized Controlled Trial" OR "Clinical Trial" OR "Controlled Clinical Trial")	
**FILTERS USED**:<br>None	**NUMBER OF DOCUMENTS FOUND**: 34
