# Supplementary material for: Antioxidants as Therapeutic Tools in the Management of COPD: A Systematic Review with Meta-Analysis
Source: Antioxidants (Basel). 2026 Apr 2;15(4):446. doi: 10.3390/antiox15040446 (PMC13113252; doi:10.3390/antiox15040446)
Supplement: Supplementary file 1 [file antioxidants-15-00446-s001.zip › Supplementary Table S3.pdf]

**CASPe**: **Critical Appraisal Skills Programme**	
**A) ARE THE TEST RESULTS VALID?**<br>**(elimination questions; only if the answers to the first two questions are "yes" is it worth continuing to answer)**	
Is the essay focused on a clearly defined question?	Yes:<br>I do not know:<br>NO:
Was the assignment of patients to treatments random?	Yes:<br>I do not know:<br>NO:
Were all patients who entered the study adequately considered until the end of the study?	Yes:<br>I do not know:<br>NO:
"DETAIL" QUESTIONS	
Was blinding maintained for:<br>· Patients<br>· Clinicians<br>· Study staff?	Yes:<br>I do not know:<br>NO:
Were the groups similar at the beginning of the trial?	Yes:<br>I do not know:<br>NO:
Apart from the intervention under study, were the groups treated equally?	Yes:<br>I do not know:<br>NO:
**B) WHAT ARE THE RESULTS?**	
Is the effect of the treatment very significant?	Yes:<br>I do not know:<br>NO:
How accurate is this effect?	Yes:<br>I do not know:<br>NO:
**C) ARE THE RESULTS APPLICABLE IN YOUR ENVIRONMENT?**	
Can these results be applied to your local environment or population?	Yes:<br>I do not know:<br>NO:
Were all clinically relevant results considered?	Yes:<br>I do not know:<br>NO:
Do the benefits outweigh the risks and costs?	SÍ:<br><br>NO:
