# Supplementary material for: Antioxidants as Therapeutic Tools in the Management of COPD: A Systematic Review with Meta-Analysis
Source: Antioxidants (Basel). 2026 Apr 2;15(4):446. doi: 10.3390/antiox15040446 (PMC13113252; doi:10.3390/antiox15040446)
Supplement: Supplementary file 1 [file antioxidants-15-00446-s001.zip › Supplementary Table S4.pdf]

<table>
<tr><td colspan="3">STUDY NUMBER: 1</td></tr>
<tr><td colspan="3">TITLE: A randomized, double-blind, placebo-controlled study evaluating the efficacy of propolis and N-acetylcysteine in exacerbations of chronic obstructive pulmonary disease</td></tr>
<tr><td>AUTHORS; YEAR OF PUBLICATION</td><td colspan="2">I. BUHA, M. MIRIĆ, A. AGIĆ, et al.
2022</td></tr>
<tr><td>ARTICLE IDENTIFIERS</td><td colspan="2">Eur Rev Med Pharmacol Sci. 2022 Jul;26(13):4809-4815. doi: 10.26355/eurrev_202207_29206. PMID: 35856373.</td></tr>
<tr><td>TYPE OF STUDY</td><td colspan="2">Double-blind randomized clinical trial</td></tr>
<tr><td>PARTICIPANTS (P)</td><td colspan="2">COPD patients (n=46)
63% were men, aged between 52 and 83, and 42.2% were smokers.</td></tr>
<tr><td>INTERVENTION (I)</td><td colspan="2">One group will receive treatment with 600 mg of NAC and 80 mg of propolis, while the control group will receive a placebo.</td></tr>
<tr><td>COMPARISON (C)</td><td colspan="2">Placebo group</td></tr>
<tr><td>RESULTS (O)</td><td colspan="2">There was a statistically significant difference in the Acute exacerbations COPD (AECOPD) incidence rate. The results of the study indicate that 52.6% of patients who received a placebo, 15.4% of those who received AS-600, and only 7.1% of those who received AS-1,200 showed positive results (Fisher's exact test, p = 0.013). When compared to a placebo, AECOPD frequency was significantly lower in AS-1,200 (p=0.009). When compared to a placebo, the relative risk for exacerbation was 0.29 in AS-600 and 0.13 in AS-1,200. No adverse events related to the treatment were reported.
Conclusions: The oral combination of natural propolis with NAC has been shown to be both effective and safe. Further validation is required through larger clinical trials to substantiate these findings.</td></tr>
<tr><td rowspan="7">QUALITY OF THE ARTICLE</td><td colspan="2">A) ARE THE TRIAL RESULTS VALID? (elimination questions; only if the first two questions are answered “yes” is it worth continuing to answer)</td></tr>
<tr><td>1. Is the trial focused on a clearly defined question?</td><td>YES: Yes
I DON'T KNOW:
NO:</td></tr>
<tr><td>2. Was the allocation of patients to treatments random?</td><td>YES: Yes
I DON'T KNOW:
NO:</td></tr>
<tr><td>3. Were all patients who entered the study adequately considered until the end of the study?</td><td>YES: Yes
I DON'T KNOW:
NO:</td></tr>
<tr><td colspan="2">“DETAIL” QUESTIONS</td></tr>
<tr><td>4. Was blinding maintained for:
• Patients
• Clinicians
• Study staff?</td><td>YES: Ues
I DON'T KNOW:
NO:</td></tr>
<tr><td>5. Were the groups similar at the start of the trial?</td><td>YES: Yes
I DON'T KNOW:
NO:</td></tr>
</table>

	6. Apart from the intervention under study, were the groups treated equally?	YES: Yes<br>I DON'T KNOW:<br>NO:
	B) WHAT ARE THE RESULTS?	
	7. Is the effect of the treatment significant?	YES: Yes<br>I DON'T KNOW:<br>NO:
	8. Was this effect accurate?	YES: Yes<br>I DON'T KNOW:<br>NO:
	C) ARE THE RESULTS APPLICABLE IN YOUR ENVIRONMENT?	
	9. Can these results be applied to your local environment or population?	YES: Yes<br>I DON'T KNOW:<br>NO:
	10. Were all clinically relevant results taken into account?	YES: Yes<br>I DON'T KNOW:<br>NO:
	11. Do the benefits outweigh the risks and costs?	YES: Yes<br>I DON'T KNOW:<br>NO:
CASP score	11/11	

STUDY NUMBER: 2		
TITLE: **A Randomized, Doubled-Blind Clinical Trial on the Effect of Zataria multiflora on Clinical Symptoms, Oxidative Stress, and C-Reactive Protein in COPD Patients**		
AUTHORS; YEAR OF PUBLICATION	Vahideh Ghorani, PhD, Omid Rajabi, PhD, et al.<br>2020-07	
ARTICLE IDENTIFIERS	J Clin Pharmacol. 2020 Jul;60(7):867-878. doi: 10.1002/jcph.1586. Epub 2020 Mar 23. PMID: 32202330.	
TYPE OF STUDY	Randomized clinical trial	
PARTICIPANTS (P)	COPD patients (n=45)	
INTERVENTION (I)	A total of 45 patients were divided into three groups: a placebo group and two groups that received 3 mg/kg/day or 6 mg/kg/day of Z. multiflora extract (Z3 and Z6) for a period of two months.<br>The clinical symptoms, pulmonary function tests, oxidative stress, and serum C-reactive protein levels of the patients were evaluated at three distinct time points: pretreatment (step 0), and 1 (step I) and 2 (step II) months after treatment.	
COMPARISON (C)	Placebo group	
RESULTS (O)	The clinical symptoms, like breathlessness and chest wheeze, in the Z3- and Z6-treated groups got a lot better 1 and 2 months after treatment compared to the starting point (P < .01 to P < .001). The Z6-treated group just had sputum production. After two months of treatment with Z3 and Z6, the FEV1 had increased a lot (P < .05 to P < .01). After two months of treatment with Z6, there was a big drop in the levels of malondialdehyde and nitrite compared to the starting point (P < .05 to P < .01). The thiol levels in the Z6 group, as well as the superoxide dismutase and catalase activities in both groups treated with the extract, went up significantly in step II compared with step 0 (P < .05 to P < .01). At the end of the study, the C-reactive protein levels were a lot lower than they were at step 0 in both the groups that were treated (P < .05 for both cases). Treatment with Z. multiflora for two months helped COPD patients with their symptoms, lung function, oxidative stress, and C-reactive protein.	
QUALITY OF THE ARTICLE	A) ARE THE TRIAL RESULTS VALID? (elimination questions; only if the first two questions are answered "yes" is it worth continuing to answer)	
	1. Is the trial focused on a clearly defined question?	YES: Yes<br>I DON'T KNOW:<br>NO:
	2. Was the allocation of patients to treatments random?	YES: Yes<br>I DON'T KNOW:<br>NO:
	3. Were all patients who entered the study adequately considered until the end of the study?	YES:<br>I DON'T KNOW: Not explained<br>NO:
	"DETAIL" QUESTIONS	
	4. Was blinding maintained for:<br>• Patients<br>• Clinicians<br>• Study staff?	YES: Yes<br>I DON'T KNOW:<br>NO:

	5. Were the groups similar at the start of the trial?	YES: Yes<br>I DON'T KNOW:<br>NO:
	6. Apart from the intervention under study, were the groups treated equally?	YES: Yes<br>I DON'T KNOW:<br>NO:
	B) WHAT ARE THE RESULTS?	
	7. Is the effect of the treatment significant?	YES: Yes<br>I DON'T KNOW:<br>NO:
	8. Was this effect accurate?	YES:<br>I DON'T KNOW: Not explained<br>NO:
	C) ARE THE RESULTS APPLICABLE IN YOUR ENVIRONMENT?	
	9. Can these results be applied to your local environment or population?	YES: Yes<br>I DON'T KNOW:<br>NO:
	10. Were all clinically relevant results taken into account?	YES: Yes<br>I DON'T KNOW:<br>NO:
	11. Do the benefits outweigh the risks and costs?	YES: Yes<br>I DON'T KNOW:<br>NO:
CASP score	9/11	

<table>
<tr><td colspan="3">STUDY NUMBER: 3</td></tr>
<tr><td colspan="3">TITLE: Changes in Blood Markers of Oxidative Stress, Inflammation and Cardiometabolic Patients with COPD after Eccentric and Concentric Cycling Training</td></tr>
<tr><td>AUTHORS; YEAR OF PUBLICATION</td><td colspan="2">Mayalen Valero-Breton, Denisse Valladares-Ide, et al. 2023-02-11</td></tr>
<tr><td>ARTICLE IDENTIFIERS</td><td colspan="2">Nutrients. 2023 Feb 11;15(4):908. doi: 10.3390/nu15040908. PMID: 36839267; PMCID: PMC9966444.</td></tr>
<tr><td>TYPE OF STUDY</td><td colspan="2">Randomized clinical trial</td></tr>
<tr><td>PARTICIPANTS (P)</td><td colspan="2">COPD patients</td></tr>
<tr><td>INTERVENTION (I)</td><td colspan="2">This study looked at the effects of 12 weeks of eccentric cycling training (ECC) and conventional concentric training (CONC) on markers of oxidative stress, inflammation, and cardiometabolic health in patients with COPD, measured at rest. What's more, because oxidative stress can be caused by a bit of intense exercise, authors looked at how changes in oxidative stress markers after a standard submaximal workout affected ECC and CONC, before and after training in patients with COPD.</td></tr>
<tr><td>COMPARISON (C)</td><td colspan="2">COPD ECC and CONC groups compared to each other</td></tr>
<tr><td>RESULTS (O)</td><td colspan="2">Prior to and following the training period, a series of physiological assessments were conducted, including peak oxygen consumption, maximal power output (VO2peak and POmax), and time-to-exhaustion (TTE) tests. The plasma antioxidant and oxidative markers, insulin resistance, lipid profile, and systemic inflammation markers were measured before and after training at rest. VO2peak, POmax and TTE remained unchanged after ECC and CONC. CONC induced an increase in antioxidants ($p = 0.01$), while ECC decreased antioxidant ($p = 0.02$) markers measured at rest. CONC induced a comparatively minor increase in oxidative stress following TTE ($p = 0.04$), and a decrease in insulin resistance ($p = 0.0006$) in comparison with the baseline.
Conclusion: The results of this study indicate that CONC training resulted in an enhancement of insulin sensitivity, an increase in antioxidant capacity at rest, and a reduction in exercise-induced oxidative stress in patients diagnosed with moderate COPD.</td></tr>
<tr><td rowspan="6">QUALITY OF THE ARTICLE</td><td colspan="2">A) ARE THE TRIAL RESULTS VALID? (elimination questions; only if the first two questions are answered “yes” is it worth continuing to answer)</td></tr>
<tr><td>1. Is the trial focused on a clearly defined question?</td><td>YES: Yes
I DON'T KNOW:
NO:</td></tr>
<tr><td>2. Was the allocation of patients to treatments random?</td><td>YES: Yes
I DON'T KNOW:
NO:</td></tr>
<tr><td>3. Were all patients who entered the study adequately considered until the end of the study?</td><td>YES: Yes
I DON'T KNOW:
NO:</td></tr>
<tr><td colspan="2">“DETAIL” QUESTIONS</td></tr>
<tr><td>4. Was blinding maintained for:
• Patients
• Clinicians</td><td>YES:
I DON'T KNOW:
NO: No</td></tr>
</table>

	• Study staff?	
	5. Were the groups similar at the start of the trial?	YES: Yes<br>I DON'T KNOW:<br>NO:
	6. Apart from the intervention under study, were the groups treated equally?	YES:<br>I DON'T KNOW: I Don´t know<br>NO:
	B) WHAT ARE THE RESULTS?	
	7. Is the effect of the treatment significant?	YES: Yes<br>I DON'T KNOW:<br>NO:
	8. Was this effect accurate?	YES: Yes<br>I DON'T KNOW:<br>NO:
	C) ARE THE RESULTS APPLICABLE IN YOUR ENVIRONMENT?	
	9. Can these results be applied to your local environment or population?	YES: Yes<br>I DON'T KNOW:<br>NO:
	10. Were all clinically relevant results taken into account?	YES: Yes<br>I DON'T KNOW:<br>NO:
	11. Do the benefits outweigh the risks and costs?	YES: Yes<br>I DON'T KNOW:<br>NO:
CASP score	9/11	

<table>
<tr><td colspan="3">STUDY NUMBER: 4</td></tr>
<tr><td colspan="3">TITLE: Dietary nitrate supplementation to enhance exercise capacity in hypoxic COPD: EDEN-OX, a double-blind, placebo-controlled, randomised cross-over study</td></tr>
<tr><td>AUTHORS; YEAR OF PUBLICATION</td><td colspan="2">Matthew J. Pavitt, Adam Lewis, Sara C. Buttery, Bernadette O. Fernandez, et al. 2022</td></tr>
<tr><td>ARTICLE IDENTIFIERS</td><td colspan="2">Thorax. 2022 Oct;77(10):968-975. doi: 10.1136/thoraxjnl-2021-217147. Epub 2021 Dec 1. PMID: 34853156.</td></tr>
<tr><td>TYPE OF STUDY</td><td colspan="2">Randomized, placebo-controlled, computer-generated block-design crossover clinical trial</td></tr>
<tr><td>PARTICIPANTS (P)</td><td colspan="2">COPD who were established users of long-term oxygen therapy</td></tr>
<tr><td>INTERVENTION (I)</td><td colspan="2">Participants were required to perform an endurance shuttle walk test, utilising their prescribed oxygen, three hours after consuming either 140 mL of nitrate-rich beetroot juice (BRJ) (12.9 mmol nitrate) or a placebo (nitrate-depleted BRJ).</td></tr>
<tr><td>COMPARISON (C)</td><td colspan="2">COPD patients treated with placebo</td></tr>
<tr><td>RESULTS (O)</td><td colspan="2">A total of 20 participants were recruited for the study and all of them completed it. The administration of nitrate-rich BRJ supplementation resulted in a significant increase in the duration of exercise endurance time among all participants, as compared with the placebo group. The median (interquartile range) value for the former was 194.6 (147.5-411.7) seconds, while for the latter it was 159.1 (121.9-298.5) seconds. The estimated treatment effect was 62 (33-106) seconds (p<0.0001). Supplementation also improved endothelial function: The NR-BRJ group demonstrated a 4.1% increase (95% confidence interval: -1.1% to 14.8%), while the placebo group exhibited a -5.0% decrease (95% confidence interval: -10.6% to -0.6%) (p=0.0003).
Conclusion: Acute dietary nitrate supplementation has been demonstrated to enhance exercise endurance in patients with COPD who require supplemental oxygen.</td></tr>
<tr><td rowspan="7">QUALITY OF THE ARTICLE</td><td colspan="2">A) ARE THE TRIAL RESULTS VALID? (elimination questions; only if the first two questions are answered “yes” is it worth continuing to answer)</td></tr>
<tr><td>1. Is the trial focused on a clearly defined question?</td><td>YES: Yes
I DON'T KNOW:
NO:</td></tr>
<tr><td>2. Was the allocation of patients to treatments random?</td><td>YES: Yes
I DON'T KNOW:
NO:</td></tr>
<tr><td>3. Were all patients who entered the study adequately considered until the end of the study?</td><td>YES: Yes
I DON'T KNOW:
NO:</td></tr>
<tr><td colspan="2">“DETAIL” QUESTIONS</td></tr>
<tr><td>4. Was blinding maintained for:
• Patients
• Clinicians
• Study staff?</td><td>YES: Yes
I DON'T KNOW:
NO:</td></tr>
<tr><td>5. Were the groups similar at the start of the trial?</td><td>YES: Yes
I DON'T KNOW:</td></tr>
</table>

		NO:
	6. Apart from the intervention under study, were the groups treated equally?	YES: Yes<br>I DON'T KNOW:<br>NO:
	B) WHAT ARE THE RESULTS?	
	7. Is the effect of the treatment significant?	YES: Yes<br>I DON'T KNOW:<br>NO:
	8. Was this effect accurate?	YES:<br>I DON'T KNOW: Not explained<br>NO:
	C) ARE THE RESULTS APPLICABLE IN YOUR ENVIRONMENT?	
	9. Can these results be applied to your local environment or population?	YES: Yes<br>I DON'T KNOW:<br>NO:
	10. Were all clinically relevant results taken into account?	YES: Yes<br>I DON'T KNOW:<br>NO:
	11. Do the benefits outweigh the risks and costs?	YES: Yes<br>I DON'T KNOW:<br>NO:
CASP score	10/11	

STUDY NUMBER: 5		
TITLE: **Effect of Crocin From Saffron (Crocus sativus L.) Supplementation on Oxidant/Antioxidant Markers, Exercise Capacity, and Pulmonary Function Tests in COPD Patients: A Randomized, Double-Blind, Placebo-Controlled Trial**		
AUTHORS; YEAR OF PUBLICATION	Hassan Ghobadi, Nasim Abdollahi, et al. 2022	
ARTICLE IDENTIFIERS	Front Pharmacol. 2022 Apr 20;13:884710. doi: 10.3389/fphar.2022.884710. PMID: 35517806; PMCID: PMC9065288.	
TYPE OF STUDY	Randomized, Double-Blind, Placebo-Controlled Trial	
PARTICIPANTS (P)	COPD patients who were divided into placebo and intervention groups (n=23 per group).	
INTERVENTION (I)	The intervention group was administered crocin supplementation (30 mg/day for 12 weeks), while the control group received a placebo. Pulmonary function tests (PFTs), as well as the 6-minute walking distance test (6MWD) to assess exercise capacity, and serum levels of total oxidant status (TOS), total antioxidant capacity (TAOC), and NF-kB, were measured pre- and post-intervention using ELISA tests.	
COMPARISON (C)	COPD patients treated with placebo	
RESULTS (O)	The administration of crocin to COPD patients over a period of 12 weeks resulted in a decline in serum levels of TOS and NF-κB, accompanied by an increase in TAOC. Furthermore, the results of the 6MWD test demonstrate an enhancement in patients' exercise capacity.<br>Conclusion: Crocin supplementation has been demonstrated to effectively establish oxidant/antioxidant balance and improve inflammatory conditions in patients with COPD.	
QUALITY OF THE ARTICLE	A) ARE THE TRIAL RESULTS VALID? (elimination questions; only if the first two questions are answered “yes” is it worth continuing to answer)	
	1. Is the trial focused on a clearly defined question?	YES: Yes<br>I DON'T KNOW:<br>NO:
	2. Was the allocation of patients to treatments random?	YES: Yes<br>I DON'T KNOW:<br>NO:
	3. Were all patients who entered the study adequately considered until the end of the study?	YES: Yes<br>I DON'T KNOW:<br>NO:
	“DETAIL” QUESTIONS	
	4. Was blinding maintained for:<br>• Patients<br>• Clinicians<br>• Study staff?	YES: Yes<br>I DON'T KNOW:<br>NO:
	5. Were the groups similar at the start of the trial?	YES: Yes<br>I DON'T KNOW:<br>NO:

	6. Apart from the intervention under study, were the groups treated equally?	YES: Yes<br>I DON'T KNOW:<br>NO:
	B) WHAT ARE THE RESULTS?	
	7. Is the effect of the treatment significant?	YES: Yes<br>I DON'T KNOW:<br>NO:
	8. Was this effect accurate?	YES:<br>I DON'T KNOW: Not explained<br>NO:
	C) ARE THE RESULTS APPLICABLE IN YOUR ENVIRONMENT?	
	9. Can these results be applied to your local environment or population?	YES: Yes<br>I DON'T KNOW:<br>NO:
	10. Were all clinically relevant results taken into account?	YES: Yes<br>I DON'T KNOW:<br>NO:
	11. Do the benefits outweigh the risks and costs?	YES: Yes<br>I DON'T KNOW:<br>NO:
CASP score	10/11	

<table>
<tr><td colspan="3">STUDY NUMBER: 6</td></tr>
<tr><td colspan="3">TITLE: Effect of crocin of Crocus sativus L. on serum inflammatory markers (IL-6 and TNF-α) in chronic obstructive pulmonary disease patients: a randomised, double-blind, placebo-controlled trial</td></tr>
<tr><td>AUTHORS; YEAR OF PUBLICATION</td><td colspan="2">Mohammad Reza Aslani, Nasim Abdollahi, et al. 2023</td></tr>
<tr><td>ARTICLE IDENTIFIERS</td><td colspan="2">Br J Nutr. 2023 Aug 14;130(3):446-453. doi: 10.1017/S0007114522003397. Epub 2023 Jan 11. PMID: 36628554.</td></tr>
<tr><td>TYPE OF STUDY</td><td colspan="2">Randomised, double-blind, placebo-controlled trial</td></tr>
<tr><td>PARTICIPANTS (P)</td><td colspan="2">COPD patients (n=57). Two groups intervention and placebo groups.</td></tr>
<tr><td>INTERVENTION (I)</td><td colspan="2">To investigate the preventive effects of crocin supplementation on serum concentrations of IL-6 and TNF-α, as well as on exercise capacity and pulmonary function test (PFT) results. The intervention group received crocin (15 mg twice daily for 12 weeks) as a supplement. Measurement of serum levels of IL-6 and TNF-α. PFT and exercise capacity were assessed using the 6-minute walk test (6MWT), which was performed at the beginning and end of the study.</td></tr>
<tr><td>COMPARISON (C)</td><td colspan="2">COPD patients with no intervention (Placebo group)</td></tr>
<tr><td>RESULTS (O)</td><td colspan="2">Compared with those in the placebo group, crocin improved the results of PFT (P < 0.05) and 6-MWD (P < 0.001) and exerted preventive effects by increasing serum IL-6 levels in patients with COPD (P < 0.05). Crocin intervention significantly lowered serum TNF-α levels at the end of the study (P < 0.01).
Conclusion: The present findings suggest that crocin supplementation improves exercise capacity and PFT in patients with COPD by reducing serum levels of inflammatory factors.</td></tr>
<tr><td rowspan="8">QUALITY OF THE ARTICLE</td><td colspan="2">A) ARE THE TRIAL RESULTS VALID? (elimination questions; only if the first two questions are answered “yes” is it worth continuing to answer)</td></tr>
<tr><td>1. Is the trial focused on a clearly defined question?</td><td>YES: Yes
I DON'T KNOW:
NO:</td></tr>
<tr><td>2. Was the allocation of patients to treatments random?</td><td>YES: Yes
I DON'T KNOW:
NO:</td></tr>
<tr><td>3. Were all patients who entered the study adequately considered until the end of the study?</td><td>YES: Yes
I DON'T KNOW:
NO:</td></tr>
<tr><td colspan="2">“DETAIL” QUESTIONS</td></tr>
<tr><td>4. Was blinding maintained for:
• Patients
• Clinicians
• Study staff?</td><td>YES: Yes
I DON'T KNOW:
NO:</td></tr>
<tr><td>5. Were the groups similar at the start of the trial?</td><td>YES: Yes
I DON'T KNOW:
NO:</td></tr>
</table>

	6. Apart from the intervention under study, were the groups treated equally?	YES: Yes<br>I DON'T KNOW:<br>NO:
	B) WHAT ARE THE RESULTS?	
	7. Is the effect of the treatment significant?	YES: Yes<br>I DON'T KNOW:<br>NO:
	8. Was this effect accurate?	YES: Yes<br>I DON'T KNOW:<br>NO:
	C) ARE THE RESULTS APPLICABLE IN YOUR ENVIRONMENT?	
	9. Can these results be applied to your local environment or population?	YES: Yes<br>I DON'T KNOW:<br>NO:
	10. Were all clinically relevant results taken into account?	YES: Yes<br>I DON'T KNOW:<br>NO:
	11. Do the benefits outweigh the risks and costs?	YES: Yes<br>I DON'T KNOW:<br>NO:
CASP score	11/11	

<table>
<tr><td colspan="3">STUDY NUMBER: 7</td></tr>
<tr><td colspan="3">TITLE: Effect of high-dose N-acetylcysteine on exacerbations and lung function in patients with mild-to-moderate COPD: a double blind, parallel group, multicentre randomised clinical trial.</td></tr>
<tr><td>AUTHORS; YEAR OF PUBLICATION</td><td colspan="2">Yumin Zhou, Fan Wu, Zhe Shi, et al.
2024.</td></tr>
<tr><td>ARTICLE IDENTIFIERS</td><td colspan="2">Nat Commun. 2024 Sep 30;15(1):8468. doi: 10.1038/s41467-024-51079-1. PMID: 39349461; PMCID: PMC11442465.</td></tr>
<tr><td>TYPE OF STUDY</td><td colspan="2">Multicenter, randomized, double-blind, placebo-controlled trial.</td></tr>
<tr><td>PARTICIPANTS (P)</td><td colspan="2">A total of 924 patients with mild to moderate COPD completed the two-year follow-up: 460 in the placebo group and 464 in the N-acetylcysteine group.</td></tr>
<tr><td>INTERVENTION (I)</td><td colspan="2">Patients diagnosed with mild to moderate COPD were methodically randomised to receive high doses of N-acetylcysteine (600 mg, twice daily) or placebo (twice daily) for a period of two years.</td></tr>
<tr><td>COMPARISON (C)</td><td colspan="2">COPD patients treated with placebo.</td></tr>
<tr><td>RESULTS (O)</td><td colspan="2">The annual rate of total exacerbations was not significantly different between the N-acetylcysteine group and the placebo group (0.65 vs. 0.72 per patient-year; relative risk (RR), 0.90; 95% confidence interval (CI), 0.80-1.02; p=0.10). There were no significant differences in FEV1 before bronchodilator administration at 24 months. The present study definitively examined the effects of long-term treatment with high doses of N-acetylcysteine on the annual rate of total exacerbations and lung function in patients diagnosed with mild to moderate COPD.
Conclusions: The treatment did not significantly reduce the annual rate of total exacerbations or improve lung function.</td></tr>
<tr><td rowspan="8">QUALITY OF THE ARTICLE</td><td colspan="2">A) ARE THE TRIAL RESULTS VALID? (elimination questions; only if the first two questions are answered “yes” is it worth continuing to answer)</td></tr>
<tr><td>1. Is the trial focused on a clearly defined question?</td><td>YES: Yes
I DON'T KNOW:
NO:</td></tr>
<tr><td>2. Was the allocation of patients to treatments random?</td><td>YES: Yes
I DON'T KNOW:
NO:</td></tr>
<tr><td>3. Were all patients who entered the study adequately considered until the end of the study?</td><td>YES: Yes
I DON'T KNOW:
NO:</td></tr>
<tr><td colspan="2">“DETAIL” QUESTIONS</td></tr>
<tr><td>4. Was blinding maintained for:
• Patients
• Clinicians
• Study staff?</td><td>YES: Yes, double-blind
I DON'T KNOW:
NO:</td></tr>
<tr><td>5. Were the groups similar at the start of the trial?</td><td>YES: Yes
I DON'T KNOW:
NO:</td></tr>
<tr><td>6. Apart from the intervention under study, were the groups treated</td><td>YES: Yes
I DON'T KNOW:</td></tr>
</table>

	equally?	NO:
	B) WHAT ARE THE RESULTS?	
	7. Is the effect of the treatment significant?	YES:<br>I DON'T KNOW:<br>NO: No
	8. Was this effect accurate?	YES:<br>I DON'T KNOW: Not explained<br>NO:
	C) ARE THE RESULTS APPLICABLE IN YOUR ENVIRONMENT?	
	9. Can these results be applied to your local environment or population?	YES: Yes<br>I DON'T KNOW:<br>NO:
	10. Were all clinically relevant results taken into account?	YES: Yes<br>I DON'T KNOW:<br>NO:
	11. Do the benefits outweigh the risks and costs?	YES: Yes<br>I DON'T KNOW:<br>NO:
CASP score	10/11	

STUDY NUMBER: 8		
TITLE: **Effects of antioxidant nutrients on muscle mass, strength and function in COPD patients: A meta-analysis of randomized controlled trials**		
AUTHORS; YEAR OF PUBLICATION	Qinman He, Pan Yang, et al. 2025	
ARTICLE IDENTIFIERS	PLoS One. 2025 Jan 17;20(1):e0316842. doi: 10.1371/journal.pone.0316842. PMID: 39823472; PMCID: PMC11741611.	
TYPE OF STUDY	Meta-analysis of randomized controlled trials	
PARTICIPANTS (P)	Among all the studies selected, a total of 595 patients diagnosed with COPD were studied.	
INTERVENTION (I)	A bibliographic search was conducted to assess the effects of antioxidant nutrients on muscle mass, strength and function in COPD patients	
COMPARISON (C)	COPD patients non receiving antioxidant nutrients	
RESULTS (O)	In terms of muscle mass, patients receiving antioxidant nutrients exhibited a significantly elevated lean body mass index in comparison with those not receiving antioxidant nutrients (pooled WMD: 0.903, 95% CI: 0.264, 1.541, P = 0.006). For patients who did not participate in lung rehabilitation plans while receiving nutritional interventions, antioxidant nutrients resulted in a significantly higher lean body mass index (pooled WMD: 1.360, 95% CI: 0.560, 2.160, P = 0.001). With regard to muscle strength, patients in the antioxidant nutrient intervention group exhibited significantly higher hand grip strength (HGS) in comparison to those in the non-antioxidant nutrient intervention group (pooled WMD: 1.976, 95% CI: 1.337, 2.615, P < 0.001). Patients receiving antioxidant nutrients exhibited significantly greater inspiratory muscle strength (MIP) in comparison to those not receiving antioxidant nutrients (pooled WMD: 8.127, 95% CI: 2.677, 13.577, P = 0.003).<br>Conclusion: Antioxidant nutrient intervention significantly improved handgrip strength (HGS), maximum inspiratory pressure (MIP) and lean body mass index (BMI) in patients with COPD. Clinicians should consider increasing food intake or supplementation rich in antioxidants in the treatment plan of patients with COPD.	
QUALITY OF THE ARTICLE	A) ARE THE TRIAL RESULTS VALID? (elimination questions; only if the first two questions are answered “yes” is it worth continuing to answer)	
	1. Is the trial focused on a clearly defined question?	YES: Yes<br>I DON'T KNOW:<br>NO:
	2. Was the allocation of patients to treatments random?	YES: Yes<br>I DON'T KNOW:<br>NO:
	3. Were all patients who entered the study adequately considered until the end of the study?	YES: Yes<br>I DON'T KNOW:<br>NO:
	“DETAIL” QUESTIONS	
	4. Was blinding maintained for:<br>• Patients<br>• Clinicians<br>• Study staff?	YES: Yes<br>I DON'T KNOW:<br>NO:

	5. Were the groups similar at the start of the trial?	YES: Yes<br>I DON'T KNOW:<br>NO:
	6. Apart from the intervention under study, were the groups treated equally?	YES: Yes<br>I DON'T KNOW:<br>NO:
	B) WHAT ARE THE RESULTS?	
	7. Is the effect of the treatment significant?	YES: Yes<br>I DON'T KNOW:<br>NO:
	8. Was this effect accurate?	YES: Yes<br>I DON'T KNOW:<br>NO:
	C) ARE THE RESULTS APPLICABLE IN YOUR ENVIRONMENT?	
	9. Can these results be applied to your local environment or population?	YES: Yes<br>I DON'T KNOW:<br>NO:
	10. Were all clinically relevant results taken into account?	YES: Yes<br>I DON'T KNOW:<br>NO:
	11. Do the benefits outweigh the risks and costs?	YES: Yes<br>I DON'T KNOW:<br>NO:
CASP score	11/11	

<table>
<tr><td colspan="3">STUDY NUMBER: 9</td></tr>
<tr><td colspan="3">TITLE: Efficacy of 12 weeks oral beta-alanine supplementation in patients with chronic obstructive pulmonary disease: a double-blind, randomized, placebo-controlled trial</td></tr>
<tr><td>AUTHORS; YEAR OF PUBLICATION</td><td colspan="2">Jana De Brandt, Wim Derave, et al. 2022-08-17</td></tr>
<tr><td>ARTICLE IDENTIFIERS</td><td colspan="2">J Cachexia Sarcopenia Muscle. 2022 Oct;13(5):2361-2372. doi: 10.1002/jcsm.13048. Epub 2022 Aug 17. PMID: 35977911; PMCID: PMC9530565.</td></tr>
<tr><td>TYPE OF STUDY</td><td colspan="2">Double-blind, randomized, placebo (PL)-controlled trial</td></tr>
<tr><td>PARTICIPANTS (P)</td><td colspan="2">COPD patients (n=40; 75% men)</td></tr>
<tr><td>INTERVENTION (I)</td><td colspan="2">12 weeks oral beta-alanine (3.2 g/day) or placebo supplementation.</td></tr>
<tr><td>COMPARISON (C)</td><td colspan="2">COPD patients treated with placebo</td></tr>
<tr><td>RESULTS (O)</td><td colspan="2">Beta-alanine supplementation has been demonstrated to increase muscle carnosine in comparison with PL in patients diagnosed with COPD whereas maximal incremental cycling capacity and the mean time to exhaustion on the constant work rate cycle test changes were not statistically significant.
Conclusion: Beta-alanine supplementation has been shown to be efficacious in augmenting muscle carnosine levels by 54% from the mean baseline value in patients with COPD, in comparison with the placebo. However, favourable changes in exercise capacity, quadriceps function, and muscle oxidative/carbonyl stress were not observed.</td></tr>
<tr><td rowspan="8">QUALITY OF THE ARTICLE</td><td colspan="2">A) ARE THE TRIAL RESULTS VALID? (elimination questions; only if the first two questions are answered “yes” is it worth continuing to answer)</td></tr>
<tr><td>1. Is the trial focused on a clearly defined question?</td><td>YES: Yes
I DON'T KNOW:
NO:</td></tr>
<tr><td>2. Was the allocation of patients to treatments random?</td><td>YES: Yes
I DON'T KNOW:
NO:</td></tr>
<tr><td>3. Were all patients who entered the study adequately considered until the end of the study?</td><td>YES: Yes
I DON'T KNOW:
NO:</td></tr>
<tr><td colspan="2">“DETAIL” QUESTIONS</td></tr>
<tr><td>4. Was blinding maintained for:
• Patients
• Clinicians
• Study staff?</td><td>YES: Yes
I DON'T KNOW:
NO:</td></tr>
<tr><td>5. Were the groups similar at the start of the trial?</td><td>YES: Yes
I DON'T KNOW:
NO:</td></tr>
<tr><td>6. Apart from the intervention under study, were the groups treated equally?</td><td>YES: Yes
I DON'T KNOW:
NO:</td></tr>
</table>

	B) WHAT ARE THE RESULTS?	
	7. Is the effect of the treatment significant?	YES: Yes<br>I DON'T KNOW:<br>NO:
	8. Was this effect accurate?	YES:<br>I DON'T KNOW: Not explained<br>NO:
	C) ARE THE RESULTS APPLICABLE IN YOUR ENVIRONMENT?	
	9. Can these results be applied to your local environment or population?	YES: Yes<br>I DON'T KNOW:<br>NO:
	10. Were all clinically relevant results taken into account?	YES: Yes<br>I DON'T KNOW:<br>NO:
	11. Do the benefits outweigh the risks and costs?	YES: Yes<br>I DON'T KNOW:<br>NO:
CASP score	10/11	

STUDY NUMBER: 10		
TITLE: **Factorial analysis of N-acetylcysteine and propolis treatment effects on symptoms, life quality and exacerbations in patients with Chronic Obstructive Pulmonary Disease (COPD): a randomized, double-blind, placebo-controlled trial**		
AUTHORS; YEAR OF PUBLICATION	V. KOLAROV, J. KOTUR STEVULJEVIĆ, et al. 2022-05	
ARTICLE IDENTIFIERS	Eur Rev Med Pharmacol Sci. 2022 May;26(9):3192-3199. doi: 10.26355/eurrev_202205_28737. PMID: 35587070.	
TYPE OF STUDY	A randomized, double-blind, placebo-controlled clinical trial	
PARTICIPANTS (P)	The study looked at 120 outpatients of all ages, between 40 and 70 years old, who had a confirmed diagnosis of COPD and had been living with the disease for at least two years. None of them had allergies to N-acetylcysteine and/or propolis. We assessed the stage of the disease using a lung function test, following the recommendations set out by the Global Initiative for Chronic Obstructive Lung Disease (GOLD). This test corresponds to stages I-IV.<br>116 patients finished the whole programme. Active smokers accounted for 34.48% (40), non-smokers for 12.93% (15), and those who had quit smoking for 52.59% (61). Comorbidities were present in 29.7% (11) of patients in Group I, 43.2% (16) in Group II, and 28.6% (12) in Group III.	
INTERVENTION (I)	Patients were divided into three groups:<br>- Group I (37 patients) received a combination of N-acetylcysteine and propolis (NACp) in powder form, at a dose of 600 mg once daily.<br>- Group II (37 patients) received 1200 mg of NACp (2x600 mg).<br>- Group III (42 patients) received a placebo.	
COMPARISON (C)	COPD patients treated with placebo	
RESULTS (O)	A total of three measurements were taken for the study. The comparison of lung function parameters showed no statistically significant differences between the groups or within them ($p > 0.05$).<br>Respiratory symptoms (sputum production and VAS for cough) during treatment with NACp showed statistically significant differences between the groups ($p < 0.05$).<br>When comparing expectoration, significant changes were observed at the start of the study and in the second and third measurements in groups I and II (Cochran's Q = 19.600; $p < 0.001$), but not in the placebo group.<br>The VAS results showed differences between the first and second, as well as between the first and third measurements, in both group I and group II (Friedman's χ^2 = 15.525; $p < 0.001$). In group III (placebo), there were no significant differences between the three measurements.<br>Conclusion: Treatment involving the administration of high doses of NAC (N-acetylcysteine) over a period of six months has been demonstrated to be both safe and beneficial in the management of symptoms associated with cough and expectoration. Furthermore, this treatment has been shown to enhance the quality of life for patients suffering from these ailments. It is evident that NACp has a significant impact on the frequency of acute exacerbations in COPD patients, with a demonstrable effect on the management of symptoms associated with the condition.	
QUALITY OF THE ARTICLE	A) ARE THE TRIAL RESULTS VALID? (elimination questions; only if the first two questions are answered “yes” is it worth continuing to answer)	
	1. Is the trial focused on a clearly defined question?	YES: Yes<br>I DON'T KNOW:<br>NO:

	2. Was the allocation of patients to treatments random?	YES: Yes<br>I DON'T KNOW:<br>NO:
	3. Were all patients who entered the study adequately considered until the end of the study?	YES: Yes<br>I DON'T KNOW:<br>NO:
	"DETAIL" QUESTIONS	
	4. Was blinding maintained for:<br>• Patients<br>• Clinicians<br>• Study staff?	YES: Yes<br>I DON'T KNOW:<br>NO:
	5. Were the groups similar at the start of the trial?	YES: Yes<br>I DON'T KNOW:<br>NO:
	6. Apart from the intervention under study, were the groups treated equally?	YES: Yes<br>I DON'T KNOW:<br>NO:
	B) WHAT ARE THE RESULTS?	
	7. Is the effect of the treatment significant?	YES: Yes<br>I DON'T KNOW:<br>NO:
	8. Was this effect accurate?	YES:<br>I DON'T KNOW: Not explained<br>NO:
	C) ARE THE RESULTS APPLICABLE IN YOUR ENVIRONMENT?	
	9. Can these results be applied to your local environment or population?	YES: Yes<br>I DON'T KNOW:<br>NO:
	10. Were all clinically relevant results taken into account?	YES: Yes<br>I DON'T KNOW:<br>NO:
	11. Do the benefits outweigh the risks and costs?	YES: Yes<br>I DON'T KNOW:<br>NO:
CASP score	10/11	

STUDY NUMBER: 11		
TITLE: **Inhaled nitric oxide improves ventilatory efficiency and exercise capacity in patients with mild COPD: A randomized-control cross-over trial**		
AUTHORS; YEAR OF PUBLICATION	Devin B. Phillips, Andrew R. Brotto, et al. 2021-03	
ARTICLE IDENTIFIERS	J Physiol. 2021 Mar;599(5):1665-1683. doi: 10.1113/JP280913. Epub 2021 Jan 25. PMID: 33428233.	
TYPE OF STUDY	Randomized placebo-controlled cross-over trial	
PARTICIPANTS (P)	Fifteen participants were diagnosed with GOLD I COPD, and a further fifteen served as the control group.<br>The total number of participants is 30.	
INTERVENTION (I)	Fifteen patients with mild COPD and 15 healthy controls completed symptom-limited cardiopulmonary exercise tests while breathing normoxic gas or 40 ppm iNO. Regarding COPD patients, a dose of 40 ppm iNO diluted with oxygen was administered to maintain inspired O_2 at ~21% without rebreathing. The placebo condition consisted of participants breathing normoxic gas (~21% O_2) administered by the same system without rebreathing.	
COMPARISON (C)	Healthy controls	
RESULTS (O)	All patients with mild COPD had values below the lower limit of normal for FEV1/FVC, while all controls remained within the normal range. Patients with mild COPD had a higher residual volume than the control group, but there were no significant differences in total lung capacity, vital capacity, functional residual capacity, or IC between the two groups. Patients with mild COPD exhibited significantly reduced DLCO (diffusing capacity for carbon monoxide), despite no intergroup differences in alveolar volume.<br>The results of the present study provide a solid foundation for the suggestion that targeting the pulmonary vasculature with more practical vasodilator therapies (e.g., sildenafil, pulsed ONi) may improve dyspnea and exercise tolerance in patients with mild COPD.<br>Conclusion: It has been demonstrated that experimental manipulation of the pulmonary circulation has the potential to positively impact dyspnoea and exercise capacity in mild COPD cases.	
QUALITY OF THE ARTICLE	A) ARE THE TRIAL RESULTS VALID? (elimination questions; only if the first two questions are answered "yes" is it worth continuing to answer)	
	1. Is the trial focused on a clearly defined question?	YES: Yes<br>I DON'T KNOW:<br>NO:
	2. Was the allocation of patients to treatments random?	YES: Yes<br>I DON'T KNOW:<br>NO:
	3. Were all patients who entered the study adequately considered until the end of the study?	YES: Yes<br>I DON'T KNOW:<br>NO:
	"DETAIL" QUESTIONS	

	4. Was blinding maintained for:<br>• Patients<br>• Clinicians<br>• Study staff?	YES: Yes<br>I DON'T KNOW:<br>NO:
	5. Were the groups similar at the start of the trial?	YES: Yes<br>I DON'T KNOW:<br>NO:
	6. Apart from the intervention under study, were the groups treated equally?	YES: Yes<br>I DON'T KNOW:<br>NO:
	B) WHAT ARE THE RESULTS?	
	7. Is the effect of the treatment significant?	YES: Yes<br>I DON'T KNOW:<br>NO:
	8. Was this effect accurate?	YES: Yes<br>I DON'T KNOW:<br>NO:
	C) ARE THE RESULTS APPLICABLE IN YOUR ENVIRONMENT?	
	9. Can these results be applied to your local environment or population?	YES: Yes<br>I DON'T KNOW:<br>NO:
	10. Were all clinically relevant results taken into account?	YES: Yes<br>I DON'T KNOW:<br>NO:
	11. Do the benefits outweigh the risks and costs?	YES: Yes<br>I DON'T KNOW:<br>NO:
CASP score	11/11	

<table>
<tr><td colspan="3">STUDY NUMBER: 12</td></tr>
<tr><td colspan="3">TITLE: Iron Replacement and Redox Balance in Non-Anemic and Mildly Anemic Iron Deficiency COPD Patients: Insights from a Clinical Trial</td></tr>
<tr><td>AUTHORS; YEAR OF PUBLICATION</td><td colspan="2">Maria Pérez-Peiró, Clara Martín-Ontiyuelo, et al. 2021-09-10</td></tr>
<tr><td>ARTICLE IDENTIFIERS</td><td colspan="2">Biomedicines. 2021 Sep 10;9(9):1191. doi: 10.3390/biomedicines9091191. PMID: 34572377; PMCID: PMC8470868.</td></tr>
<tr><td>TYPE OF STUDY</td><td colspan="2">Single-blind, unicentric, parallel-group, placebo-controlled clinical trial</td></tr>
<tr><td>PARTICIPANTS (P)</td><td colspan="2">A total of 66 COPD patients were enrolled in the study, with 44 patients assigned to the iron arm and 22 to the placebo arm.</td></tr>
<tr><td>INTERVENTION (I)</td><td colspan="2">Iron treatment. Serum levels of 3-nitrotyrosine, MDA-protein adducts, and reactive carbonyls, catalase, superoxide dismutase (SOD), glutathione, Trolox equivalent antioxidant capacity (TEAC), and iron metabolism biomarkers were quantified in both the iron-treated patients and the placebo group.</td></tr>
<tr><td>COMPARISON (C)</td><td colspan="2">COPD patients treated with placebo.</td></tr>
<tr><td>RESULTS (O)</td><td colspan="2">In the patients treated with iron compared to those given a placebo, levels of MDA-protein adducts and 3-nitrotyrosine in the serum fell significantly, while GSH levels increased and iron metabolism parameters showed marked improvement. Hepcidin has been linked to iron status parameters.
Conclusion: The findings of this randomised clinical trial demonstrated that iron replacement therapy resulted in a decline in serum oxidative stress markers, accompanied by an enhancement in GSH levels, in patients diagnosed with stable severe COPD. Hepcidin has been identified as a potential surrogate biomarker for evaluating iron status and metabolism in patients with chronic respiratory diseases.</td></tr>
<tr><td rowspan="9">QUALITY OF THE ARTICLE</td><td colspan="2">A) ARE THE TRIAL RESULTS VALID? (elimination questions; only if the first two questions are answered “yes” is it worth continuing to answer)</td></tr>
<tr><td>1. Is the trial focused on a clearly defined question?</td><td>YES: Yes
I DON'T KNOW:
NO:</td></tr>
<tr><td>2. Was the allocation of patients to treatments random?</td><td>YES: Yes
I DON'T KNOW:
NO:</td></tr>
<tr><td>3. Were all patients who entered the study adequately considered until the end of the study?</td><td>YES: Yes
I DON'T KNOW:
NO:</td></tr>
<tr><td colspan="2">“DETAIL” QUESTIONS</td></tr>
<tr><td>4. Was blinding maintained for:
• Patients
• Clinicians
• Study staff?</td><td>YES: Yes
I DON'T KNOW:
NO:</td></tr>
<tr><td>5. Were the groups similar at the start of the trial?</td><td>YES: Yes
I DON'T KNOW:
NO:</td></tr>
<tr><td>6. Apart from the intervention under study, were the groups treated</td><td>YES: Yes
I DON'T KNOW:</td></tr>
</table>

	equally?	NO:
	B) WHAT ARE THE RESULTS?	
	7. Is the effect of the treatment significant?	YES: Yes<br>I DON'T KNOW:<br>NO:
	8. Was this effect accurate?	YES: Yes<br>I DON'T KNOW:<br>NO:
	C) ARE THE RESULTS APPLICABLE IN YOUR ENVIRONMENT?	
	9. Can these results be applied to your local environment or population?	YES: Yes<br>I DON'T KNOW:<br>NO:
	10. Were all clinically relevant results taken into account?	YES: Yes<br>I DON'T KNOW:<br>NO:
	11. Do the benefits outweigh the risks and costs?	YES: Yes<br>I DON'T KNOW:<br>NO:
CASP score	11/11	

<table>
<tr><td colspan="3">STUDY NUMBER: 13</td></tr>
<tr><td colspan="3">TITLE: Melatonin supplementation enhances pulmonary rehabilitation outcomes in COPD: a randomized, double-blind, placebo-controlled study</td></tr>
<tr><td>AUTHORS; YEAR OF PUBLICATION</td><td colspan="2">Soraya Maria do Nascimento Rebouças Viana, Veralice Meireles Sales de Bruin, et al. 2023-12</td></tr>
<tr><td>ARTICLE IDENTIFIERS</td><td colspan="2">Respir Med. 2023 Dec;220:107441. doi: 10.1016/j.rmed.2023.107441. Epub 2023 Nov 7. PMID: 37944829.</td></tr>
<tr><td>TYPE OF STUDY</td><td colspan="2">Randomized, parallel-group, placebo-controlled trial</td></tr>
<tr><td>PARTICIPANTS (P)</td><td colspan="2">Thirty-nine individuals with COPD referred to a supervised pulmonary rehabilitation program were randomized to receive melatonin (3 mg/day; n = 18) or placebo (n = 21).</td></tr>
<tr><td>INTERVENTION (I)</td><td colspan="2">Treatment with melatonin (3 mg/day; n = 18) or placebo (n = 21). The investigation focused on three primary outcomes: exercise capacity, as measured by the 6-minute walk test (6MWT); health status, evaluated using the COPD assessment test; and quality of life, assessed via the Airways Questionnaire 20.</td></tr>
<tr><td>COMPARISON (C)</td><td colspan="2">Placebo group</td></tr>
<tr><td>RESULTS (O)</td><td colspan="2">At the commencement of the study, no discrepancies were identified in the demographic, anthropometric and clinical characteristics of the MLT and placebo groups. In the final analysis, the melatonin group demonstrated superiority in terms of the following outcomes: an increase in distance covered in the 6MWT (71 ± 26 vs. 25 ± 36 m; $p < 0.01$), an improvement in health status (-11 ± 6 vs. -3 ± 5; $p < 0.01$), and an enhancement in quality of life (-6.9 ± 3.0 vs. -1.9 ± 2.4; $p < 0.01$). These outcomes were compared to those observed in the placebo group.
Conclusion: The administration of melatonin over a period of 12 weeks in conjunction with pulmonary rehabilitation has been demonstrated to engender enhancements in functional capacity, health status and quality of life in patients diagnosed with COPD. These findings may have significant implications for the management of this condition.</td></tr>
<tr><td rowspan="6">QUALITY OF THE ARTICLE</td><td colspan="2">A) ARE THE TRIAL RESULTS VALID? (elimination questions; only if the first two questions are answered “yes” is it worth continuing to answer)</td></tr>
<tr><td>1. Is the trial focused on a clearly defined question?</td><td>YES: Yes
I DON'T KNOW:
NO:</td></tr>
<tr><td>2. Was the allocation of patients to treatments random?</td><td>YES: Yes
I DON'T KNOW:
NO:</td></tr>
<tr><td>3. Were all patients who entered the study adequately considered until the end of the study?</td><td>YES: Yes
I DON'T KNOW:
NO:</td></tr>
<tr><td colspan="2">“DETAIL” QUESTIONS</td></tr>
<tr><td>4. Was blinding maintained for:
• Patients
• Clinicians
• Study staff?</td><td>YES: Yes
I DON'T KNOW:
NO:</td></tr>
</table>

	5. Were the groups similar at the start of the trial?	YES: Yes<br>I DON'T KNOW:<br>NO:
	6. Apart from the intervention under study, were the groups treated equally?	YES: Yes<br>I DON'T KNOW:<br>NO:
	B) WHAT ARE THE RESULTS?	
	7. Is the effect of the treatment significant?	YES: Yes<br>I DON'T KNOW:<br>NO:
	8. Was this effect accurate?	YES:<br>I DON'T KNOW: Not explained<br>NO:
	C) ARE THE RESULTS APPLICABLE IN YOUR ENVIRONMENT?	
	9. Can these results be applied to your local environment or population?	YES: Yes<br>I DON'T KNOW:<br>NO:
	10. Were all clinically relevant results taken into account?	YES: Yes<br>I DON'T KNOW:<br>NO:
	11. Do the benefits outweigh the risks and costs?	YES: Yes<br>I DON'T KNOW:<br>NO:
CASP score	10/11	

STUDY NUMBER: 14		
TITLE: **N-acetylcysteine Treatment in Chronic Obstructive Pulmonary Disease (COPD) and Chronic Bronchitis/Pre-COPD: Distinct Meta-analyses**		
AUTHORS; YEAR OF PUBLICATION	Alberto Papi, Franco Alfano, et al. 2024-05	
ARTICLE IDENTIFIERS	Arch Bronconeumol. 2024 May;60(5):269-278. English, Spanish. doi: 10.1016/j.arbres.2024.03.010. Epub 2024 Mar 18. PMID: 38555190.	
TYPE OF STUDY	Systematic literature search in the Cochrane Central Register of Controlled Trials (CENTRAL), PubMed, and ClinicalTrials.gov from inception to February 5, 2024.	
PARTICIPANTS (P)	All randomized clinical trials (RCTs) that evaluated adult patients with COPD or CB who received oral N-acetylcysteine versus placebo for at least two consecutive months were included.	
INTERVENTION (I)	NAC administration	
COMPARISON (C)	Placebo control group	
RESULTS (O)	A total of twenty studies were included in the analysis, of which seven evaluated the use of NAC in patients exhibiting symptoms of CB/pre-COPD as a criteria for entry. The present study demonstrated that NAC-treated patients exhibited a substantial decrease in the prevalence of exacerbations in comparison with the placebo group. This observation was observed in both the COPD (incidence rate ratio (IRR)=0.76; 95% confidence interval (CI)=0.59-0.99) and CB/pre-COPD (IRR=0.81; 95% CI=0.69-0.95) populations. Sensitivity analyses in studies with duration higher than five months confirmed the overall results. CB/pre-COPD patients treated with NAC demonstrated a significantly higher probability of experiencing an improvement in symptoms and/or QoL in comparison to those administered a placebo (odds ratio (OR)=3.47; 95% CI 1.92-6.26). A similar trend was observed in the few COPD studies that were found to be evaluable. Sensitivity analyses demonstrated a significant association of NAC with improvement in symptoms and/or quality of life in both CB/pre-COPD and COPD patients.<br>Conclusions: The findings of this study provide novel data on the use of NAC in the treatment of symptoms and quality of life in addition to the prevention of exacerbations in COPD and CB/pre-COPD.	
QUALITY OF THE ARTICLE	A) ARE THE TRIAL RESULTS VALID? (elimination questions; only if the first two questions are answered “yes” is it worth continuing to answer)	
	1. Is the trial focused on a clearly defined question?	YES: Yes<br>I DON'T KNOW:<br>NO:
	2. Was the allocation of patients to treatments random?	YES: Yes<br>I DON'T KNOW:<br>NO:
	3. Were all patients who entered the study adequately considered until the end of the study?	YES: Yes<br>I DON'T KNOW:<br>NO:
	“DETAIL” QUESTIONS	
	4. Was blinding maintained for:<br>• Patients<br>• Clinicians	YES: Yes<br>I DON'T KNOW:<br>NO:

	• Study staff?	
	5. Were the groups similar at the start of the trial?	YES: Yes<br>I DON'T KNOW:<br>NO:
	6. Apart from the intervention under study, were the groups treated equally?	YES: Yes<br>I DON'T KNOW:<br>NO:
	B) WHAT ARE THE RESULTS?	
	7. Is the effect of the treatment significant?	YES: Yes<br>I DON'T KNOW:<br>NO:
	8. Was this effect accurate?	YES: Yes<br>I DON'T KNOW:<br>NO:
	C) ARE THE RESULTS APPLICABLE IN YOUR ENVIRONMENT?	
	9. Can these results be applied to your local environment or population?	YES: Yes<br>I DON'T KNOW:<br>NO:
	10. Were all clinically relevant results taken into account?	YES: Yes<br>I DON'T KNOW:<br>NO:
	11. Do the benefits outweigh the risks and costs?	YES: Yes<br>I DON'T KNOW:<br>NO:
CASP score	11/11	

STUDY NUMBER: 15		
TITLE: **Oxidative stress and total phenolics concentration in COPD patients – The eefct of execises: a ramdomized controlled trial**		
AUTHORS; YEAR OF PUBLICATION	Katarzyna Domaszewska, Sara Górna, Malwina Pietrzak and Tomasz Podgórski.<br>6 Mayo 2022	
ARTICLE IDENTIFIERS	Nutrients. 2022 May 6;14(9):1947. doi: 10.3390/nu14091947. PMID: 35565914; PMCID: PMC9105366.	
TYPE OF STUDY	Randomized Controlled Clinical Trial	
PARTICIPANTS (P)	COPD patients	
INTERVENTION (I)	The study involved the administration of a series of assessments to a cohort of patients diagnosed with COPD (n = 32). A random selection of 20 patients participated in a modified rehabilitation programme during their rehabilitation stay. The results obtained from this group were then compared with those of a control group of 12 patients who did not undergo endurance training as part of their treatment.	
COMPARISON (C)	Control group of 12 patients who did not undergo endurance training as part of their treatment.	
RESULTS (O)	In the intervention group, greater post-training increases of VO2max ($p = 0.0702$) and FEV1/FVC ($p < 0.05$; ES: 0.436) were reported. The applied CPET at each time point resulted in an increase in the All concentration ($p < 0.05$) in both the study and control groups.<br>Conclusions: The application of endurance training as a component of the rehabilitation process did not result in the exacerbation of oxidative stress or an increase in the total phenolics concentration of the blood.	
QUALITY OF THE ARTICLE	A) ARE THE TRIAL RESULTS VALID? (elimination questions; only if the first two questions are answered "yes" is it worth continuing to answer)	
	1. Is the trial focused on a clearly defined question?	YES: Yes<br>I DON'T KNOW:<br>NO:
	2. Was the allocation of patients to treatments random?	YES: Yes<br>I DON'T KNOW:<br>NO:
	3. Were all patients who entered the study adequately considered until the end of the study?	YES: Yes<br>I DON'T KNOW:<br>NO:
	"DETAIL" QUESTIONS	
	4. Was blinding maintained for:<br>• Patients<br>• Clinicians<br>• Study staff?	YES: Yes<br>I DON'T KNOW:<br>NO:
	5. Were the groups similar at the start of the trial?	YES: Yes<br>I DON'T KNOW:<br>NO:

	6. Apart from the intervention under study, were the groups treated equally?	YES: Yes<br>I DON'T KNOW:<br>NO:
	B) WHAT ARE THE RESULTS?	
	7. Is the effect of the treatment significant?	YES: Yes<br>I DON'T KNOW:<br>NO:
	8. Was this effect accurate?	YES: Yes<br>I DON'T KNOW:<br>NO:
	C) ARE THE RESULTS APPLICABLE IN YOUR ENVIRONMENT?	
	9. Can these results be applied to your local environment or population?	YES: Yes<br>I DON'T KNOW:<br>NO:
	10. Were all clinically relevant results taken into account?	YES: Yes<br>I DON'T KNOW:<br>NO:
	11. Do the benefits outweigh the risks and costs?	YES: Yes<br>I DON'T KNOW:<br>NO:
CASP score	11/11	

STUDY NUMBER: 16		
TITLE: **Residual effects of 12 weeks of power-oriented resistance training plus high-intensity interval training on muscle dysfunction, systemic oxidative damage and antioxidant capacity after 10 months of training cessation in older people with COPD.**		
AUTHORS; YEAR OF PUBLICATION	Ivan Baltasar-Fernandez, Jose Losa-Reyna, et al. 2023-09	
ARTICLE IDENTIFIERS	Scand J Med Sci Sports. 2023 Sep;33(9):1661-1676. doi: 10.1111/sms.14428. Epub 2023 Jun 15. PMID: 37322570.	
TYPE OF STUDY	Randomized controlled trial with two parallel groups.	
PARTICIPANTS (P)	A total of 21 older adults with COPD were included in the study: eight were part of the intervention group (INT) and 13 were in the control group (CON).	
INTERVENTION (I)	The subjects were assessed at baseline and 10 months after the completion of the intervention using the Short Physical Performance Battery (SPPB), the EQ-5D-5L, the vastus lateralis muscle thickness (MT), the peak pulmonary oxygen uptake (peak VO2) and the peak work rate (Wpeak), the early and late isometric rate of force development (RFD), the leg and chest press maximum muscle power (LPmax and CPmax), and the systemic oxidative damage and antioxidant capacity.	
COMPARISON (C)	INT vs CON groups	
RESULTS (O)	Following a 10-month period of reduced training, the INT group demonstrated enhanced SPPB scores (an increase of 1.0 point), health-related quality of life (an increase of 0.07 points), early RFD (an increase of 834 N · s-1), LPmax (an increase of 62.2 W), and CPmax (an increase of 16.0 W). All of these results were statistically significant ($p < 0.05$). Furthermore, a favourable outcome was observed in INT in comparison to CON with regard to MT and Wpeak (both $p < 0.05$). There were no significant between-group differences reported in peak VO2, late RFD, systemic oxidative damage, and antioxidant capacity from baseline to 10 months after the intervention was completed (all $p > 0.05$).<br>Conclusion: Twelve weeks of concurrent training was sufficient to ensure improved physical function, health-related quality of life, early RFD and maximum muscle power. Furthermore, the programme was able to preserve MT and Wpeak, but not peak VO2, late RFD, systemic oxidative damage and antioxidant capacity in the subsequent 10 months of detraining in older adults with COPD.	
QUALITY OF THE ARTICLE	A) ARE THE TRIAL RESULTS VALID? (elimination questions; only if the first two questions are answered "yes" is it worth continuing to answer)	
	1. Is the trial focused on a clearly defined question?	YES: Yes<br>I DON'T KNOW:<br>NO:
	2. Was the allocation of patients to treatments random?	YES: Yes<br>I DON'T KNOW:<br>NO:
	3. Were all patients who entered the study adequately considered until the end of the study?	YES: Yes<br>I DON'T KNOW:<br>NO:
	"DETAIL" QUESTIONS	
	4. Was blinding maintained for:<br>• Patients	YES: Yes<br>I DON'T KNOW:

	• Clinicians<br>• Study staff?	NO:
	5. Were the groups similar at the start of the trial?	YES: Yes<br>I DON'T KNOW:<br>NO:
	6. Apart from the intervention under study, were the groups treated equally?	YES: Yes<br>I DON'T KNOW:<br>NO:
	B) WHAT ARE THE RESULTS?	
	7. Is the effect of the treatment significant?	YES: Yes<br>I DON'T KNOW:<br>NO:
	8. Was this effect accurate?	YES:<br>I DON'T KNOW: Not explained<br>NO:
	C) ARE THE RESULTS APPLICABLE IN YOUR ENVIRONMENT?	
	9. Can these results be applied to your local environment or population?	YES: Yes<br>I DON'T KNOW:<br>NO:
	10. Were all clinically relevant results taken into account?	YES: Yes<br>I DON'T KNOW:<br>NO:
	11. Do the benefits outweigh the risks and costs?	YES: Yes<br>I DON'T KNOW:<br>NO:
CASP score	10/11	

STUDY NUMBER: 17		
TITLE: **Resveratrol and metabolic health in COPD: A proof-of-concept randomized controlled trial**		
AUTHORS; YEAR OF PUBLICATION	Rosanne JHCG. Beijers, Harry R. Gosker, et al. 2020-10	
ARTICLE IDENTIFIERS	Clin Nutr. 2020 Oct;39(10):2989-2997. doi: 10.1016/j.clnu.2020.01.002. Epub 2020 Jan 13. PMID: 31996311.	
TYPE OF STUDY	Randomized clinical trial	
PARTICIPANTS (P)	21 COPD patients received resveratrol (150 mg/day) (n=10) or placebo (n=11) for four weeks.	
INTERVENTION (I)	Resveratrol treatment for four weeks. Prior to and following the intervention, blood samples, quadriceps muscle and subcutaneous abdominal fat biopsies were obtained for metabolic and inflammatory profiling. The assessment of body composition was conducted using dual energy X-ray absorptiometry (DEXA) scanning.	
COMPARISON (C)	Placebo group	
RESULTS (O)	Research indicates that the mitochondrial biogenesis regulators AMPK, SIRT1 and PGC-1α, as well as mitochondrial respiration, Oxphos complexes, oxidative enzyme activities and kynurenine aminotransferases, were not improved by resveratrol. Plasma high-sensitive C-reactive protein and kynurenine levels remained unchanged following resveratrol supplementation. Resveratrol had no effect on inflammatory markers in adipose tissue, but there was a significant increase in markers of glycolysis and lipolysis compared to the placebo. Following resveratrol supplementation, there was a decrease in body weight.<br>Conclusion: The previously reported positive effects of resveratrol on skeletal muscle mitochondrial function in patients with COPD are not confirmed, but an unexpected decline in lean mass is shown.	
QUALITY OF THE ARTICLE	A) ARE THE TRIAL RESULTS VALID? (elimination questions; only if the first two questions are answered "yes" is it worth continuing to answer)	
	1. Is the trial focused on a clearly defined question?	YES: Yes<br>I DON'T KNOW:<br>NO:
	2. Was the allocation of patients to treatments random?	YES: Yes<br>I DON'T KNOW:<br>NO:
	3. Were all patients who entered the study adequately considered until the end of the study?	YES: Yes<br>I DON'T KNOW:<br>NO:
	"DETAIL" QUESTIONS	
	4. Was blinding maintained for:<br>• Patients<br>• Clinicians<br>• Study staff?	YES: Yes<br>I DON'T KNOW:<br>NO:
	5. Were the groups similar at the start of the trial?	YES: Yes<br>I DON'T KNOW:

		NO:
	6. Apart from the intervention under study, were the groups treated equally?	YES: Yes<br>I DON'T KNOW:<br>NO:
	B) WHAT ARE THE RESULTS?	
	7. Is the effect of the treatment significant?	YES:<br>I DON'T KNOW:<br>NO: No
	8. Was this effect accurate?	YES:<br>I DON'T KNOW: No explained<br>NO:
	C) ARE THE RESULTS APPLICABLE IN YOUR ENVIRONMENT?	
	9. Can these results be applied to your local environment or population?	YES: Yes<br>I DON'T KNOW:<br>NO:
	10. Were all clinically relevant results taken into account?	YES: Yes<br>I DON'T KNOW:<br>NO:
	11. Do the benefits outweigh the risks and costs?	YES: Yes<br>I DON'T KNOW:<br>NO:
CASP score	9/11	

<table>
<tr><td colspan="3">STUDY NUMBER: 18</td></tr>
<tr><td colspan="3">TITLE: Sequential inspiratory muscle exercise-noninvasive positive pressure ventilation alleviates oxidative stress in COPD by mediating SOCS5/JAK2/STAT3 pathway</td></tr>
<tr><td>AUTHORS; YEAR OF PUBLICATION</td><td colspan="2">Tang, Man Luo; 2023-10-12</td></tr>
<tr><td>ARTICLE IDENTIFIERS</td><td colspan="2">BMC Pulm Med. 2023 Oct 12;23(1):385. doi: 10.1186/s12890-023-02656-5. PMID: 37828534; PMCID: PMC10568888.</td></tr>
<tr><td>TYPE OF STUDY</td><td colspan="2">Randomized Clinical Trial</td></tr>
<tr><td>PARTICIPANTS (P)</td><td colspan="2">n=100
All subjects were randomly divided into four groups in equal proportions using a random number generator: Oxygen therapy group (21 men, 4 women), sequential noninvasive positive pressure ventilation (NIPPV) group (23 men, 2 women), inspiratory muscle training (IMT) group (20 men, 5 women), and NIPPV + IMT group (21 men, 4 women).</td></tr>
<tr><td>INTERVENTION (I)</td><td colspan="2">The objective of this study was to examine the therapeutic impact and mechanism of a novel sequential noninvasive positive pressure ventilation (NIPPV) + inspiratory muscle training (IMT) therapy.
The study examined and recorded lung function, exercise endurance, quality of life, and dyspnea symptoms. Subsequently, levels of reactive oxygen species (ROS), malondialdehyde (MDA), superoxide dismutase (SOD) and glutathione (GSH) were detected by means of an enzyme-linked immunoassay. Furthermore, changes in the expression of the suppressor of cytokine signaling 5 (SOCS5)/janus kinase 2 (JAK2)/signal transducer and activator of transcription 3 (STAT3) pathway were detected by quantitative real-time polymerase chain reaction (qRT-PCR) and western blot.</td></tr>
<tr><td>COMPARISON (C)</td><td colspan="2">Oxygen therapy group</td></tr>
<tr><td>RESULTS (O)</td><td colspan="2">Sequential treatment with NIPPV + IMT alleviated dyspnea and improved exercise tolerance and quality of life in patients with COPD, which could be related to reduced levels of oxidative stress mediated by the SOCS5/JAK2/STAT3 signaling pathway.</td></tr>
<tr><td rowspan="6">QUALITY OF THE ARTICLE</td><td colspan="2">A) ARE THE TRIAL RESULTS VALID? (elimination questions; only if the first two questions are answered “yes” is it worth continuing to answer)</td></tr>
<tr><td>1. Is the trial focused on a clearly defined question?</td><td>YES: Yes
I DON'T KNOW:
NO:</td></tr>
<tr><td>2. Was the allocation of patients to treatments random?</td><td>YES: Yes
I DON'T KNOW:
NO:</td></tr>
<tr><td>3. Were all patients who entered the study adequately considered until the end of the study?</td><td>YES: Yes
I DON'T KNOW:
NO:</td></tr>
<tr><td colspan="2">“DETAIL” QUESTIONS</td></tr>
<tr><td>4. Was blinding maintained for:
• Patients
• Clinicians</td><td>YES: Yes
I DON'T KNOW:
NO:</td></tr>
</table>

	• Study staff?	
	5. Were the groups similar at the start of the trial?	YES: Yes<br>I DON'T KNOW:<br>NO:
	6. Apart from the intervention under study, were the groups treated equally?	YES: Yes<br>I DON'T KNOW:<br>NO:
	B) WHAT ARE THE RESULTS?	
	7. Is the effect of the treatment significant?	YES: Yes<br>I DON'T KNOW:<br>NO:
	8. Was this effect accurate?	YES:<br>I DON'T KNOW: Not explained<br>NO:
	C) ARE THE RESULTS APPLICABLE IN YOUR ENVIRONMENT?	
	9. Can these results be applied to your local environment or population?	YES: Yes<br>I DON'T KNOW:<br>NO:
	10. Were all clinically relevant results taken into account?	YES: Yes<br>I DON'T KNOW:<br>NO:
	11. Do the benefits outweigh the risks and costs?	YES: Yes<br>I DON'T KNOW:<br>NO:
CASP score	10/11	

<table>
<tr><td colspan="3">STUDY NUMBER: 19</td></tr>
<tr><td colspan="3">TITLE: Therapeutic effects of black seed oil supplementation on chronic obstructive pulmonary disease patients: A randomized controlled double blind clinical trial</td></tr>
<tr><td>AUTHORS; YEAR OF PUBLICATION</td><td colspan="2">Al-Azzawi MA, AboZaid MMN, Ibrahem RAL, Sakr MA.
2020-08</td></tr>
<tr><td>ARTICLE IDENTIFIERS</td><td colspan="2">Heliyon. 2020 Aug 13;6(8):e04711. doi: 10.1016/j.heliyon.2020.e04711. PMID: 32904114; PMCID: PMC7452452.</td></tr>
<tr><td>TYPE OF STUDY</td><td colspan="2">Prospective, randomized, controlled, double-blind clinical trial</td></tr>
<tr><td>PARTICIPANTS (P)</td><td colspan="2">The study involved 91 patients with mild to moderate COPD, who were randomly divided into two groups. The first group received standard medication only (the control group; n=44), while the second group received standard medication in addition to Black Seed Oil (the BSO group; n=47).</td></tr>
<tr><td>INTERVENTION (I)</td><td colspan="2">The patients were initially assessed, and then reassessed after three months for pulmonary function tests and and inflammatory and OS parameters.</td></tr>
<tr><td>COMPARISON (C)</td><td colspan="2">Control group receiving only specific COPD treatment</td></tr>
<tr><td>RESULTS (O)</td><td colspan="2">The BSO group demonstrated a substantial decrease in oxidant and inflammatory markers, including thiobarbituric acid reactive substances (TBARS), protein carbonyl (PC) content, interleukin-6 (IL-6), and tumour necrosis factor-α (TNF-α). Concurrently, there was a notable increase in antioxidants, including superoxide dismutase (SOD), catalase (CAT), reduced glutathione (GSH), glutathione peroxidase (GPx), vitamin C, and E. Additionally, there was a significant enhancement in PFTs when compared to the control group and baseline levels.
Conclusion: Supplementation with Black Seed Oil has the potential to serve as an effective adjunct therapy, improving pulmonary functions, reducing inflammation, and addressing oxidant-antioxidant imbalance in COPD patients.</td></tr>
<tr><td rowspan="7">QUALITY OF THE ARTICLE</td><td colspan="2">A) ARE THE TRIAL RESULTS VALID? (elimination questions; only if the first two questions are answered “yes” is it worth continuing to answer)</td></tr>
<tr><td>1. Is the trial focused on a clearly defined question?</td><td>YES: Yes
I DON'T KNOW:
NO:</td></tr>
<tr><td>2. Was the allocation of patients to treatments random?</td><td>YES: Yes
I DON'T KNOW:
NO:</td></tr>
<tr><td>3. Were all patients who entered the study adequately considered until the end of the study?</td><td>YES: Yes
I DON'T KNOW:
NO:</td></tr>
<tr><td colspan="2">“DETAIL” QUESTIONS</td></tr>
<tr><td>4. Was blinding maintained for:
• Patients
• Clinicians
• Study staff?</td><td>YES: Yes
I DON'T KNOW:
NO:</td></tr>
<tr><td>5. Were the groups similar at the start of the trial?</td><td>YES: Yes
I DON'T KNOW:
NO:</td></tr>
</table>

	6. Apart from the intervention under study, were the groups treated equally?	YES: Yes<br>I DON'T KNOW:<br>NO:
	B) WHAT ARE THE RESULTS?	
	7. Is the effect of the treatment significant?	YES: Yes<br>I DON'T KNOW:<br>NO:
	8. Was this effect accurate?	YES: Yes<br>I DON'T KNOW:<br>NO:
	C) ARE THE RESULTS APPLICABLE IN YOUR ENVIRONMENT?	
	9. Can these results be applied to your local environment or population?	YES: Yes<br>I DON'T KNOW:<br>NO:
	10. Were all clinically relevant results taken into account?	YES: Yes<br>I DON'T KNOW:<br>NO:
	11. Do the benefits outweigh the risks and costs?	YES: Yes<br>I DON'T KNOW:<br>NO:
CASP score	11/11	

STUDY NUMBER: 20		
TITLE: **Withania somnifera (L.) Dunal as Add-On Therapy for COPD Patients: A Randomized, Placebo-Controlled, Double-Blind Study**		
AUTHORS; YEAR OF PUBLICATION	Singh P, Salman KA, Shameem M, Warsi MS.	
ARTICLE IDENTIFIERS	Front Pharmacol. 2022 Jun 16;13:901710. doi: 10.3389/fphar.2022.901710. PMID: 35784687; PMCID: PMC9243480.	
TYPE OF STUDY	Randomized, Placebo-Controlled, Double-Blind Study	
PARTICIPANTS (P)	N=150<br>Control group= 50<br>Placebo group= 50<br>WS group (withania somnifera)= 50	
INTERVENTION (I)	The control group received only conventional medications, i.e., fluticasone propionate or oral prednisolone and a long-acting bronchodilator (Tiate or doxiflo) as needed. The placebo group received starch capsules along with conventional medications. The WS group received WS root capsules along with conventional medications for 12 weeks.	
COMPARISON (C)	Between groups. The study's primary objective was to assess the impact of a 12-week intervention on the lung functioning, quality of life, exercise tolerance, systemic oxidative stress (OS), and systemic inflammation of the participants. The identification of WS root phytochemicals was accomplished by LC-ESI-MS. The inhibitory activity of these phytochemicals against angiotensin-converting enzyme 2 (ACE-2); the SARS-CoV-2 receptor; myeloperoxidase (MPO); and interleukin-6 (IL-6) was evaluated by in silico docking to investigate the mechanism of action of WS.	
RESULTS (O)	The study demonstrated significant improvements in pulmonary function, quality of life, and exercise tolerance in the WS group, with a notable reduction in inflammation. The manifestation of systemic oxidative stress exhibited a marked attenuation, exclusively in the WS group. Although a minor placebo effect was observed in the SGRQ test, it was not present in other tests. Withanolides, a group of natural compounds found in the roots of WS, exhibited significant inhibitory activity against the proteins ACE-2, MPO, and IL-6. This inhibitory effect was found to be more pronounced compared to that of a standard pharmaceutical drug or a known inhibitor. Furthermore, the FEV1% predicted showed a significant correlation with systemic antioxidative status (positive correlation) and malondialdehyde (MDA, negative correlation). This suggests that the antioxidative potential of WS has a significant contribution to improving lung functioning.<br>Conclusion: The present study has demonstrated, through clinical experimentation, that the administration of WS root in conjunction with conventional pharmaceuticals results in a significant amelioration of COPD symptoms, particularly in patients classified within GOLD categories 2 and 3. In silico analysis has revealed the compound to be a potent inhibitor of the SARS-CoV-2 receptor, ACE-2, as well as MPO and IL-6.	
QUALITY OF THE ARTICLE	A) ARE THE TRIAL RESULTS VALID? (elimination questions; only if the first two questions are answered “yes” is it worth continuing to answer)	
	1. Is the trial focused on a clearly defined question?	YES: Yes<br>I DON'T KNOW:<br>NO:
	2. Was the allocation of patients to treatments random?	YES: Yes<br>I DON'T KNOW:<br>NO:

	3. Were all patients who entered the study adequately considered until the end of the study?	YES: Yes<br>I DON'T KNOW:<br>NO:
	"DETAIL" QUESTIONS	
	4. Was blinding maintained for:<br>• Patients<br>• Clinicians<br>• Study staff?	YES: Yes<br>I DON'T KNOW:<br>NO:
	5. Were the groups similar at the start of the trial?	YES: Yes<br>I DON'T KNOW:<br>NO:
	6. Apart from the intervention under study, were the groups treated equally?	YES: Yes<br>I DON'T KNOW:<br>NO:
	B) WHAT ARE THE RESULTS?	
	7. Is the effect of the treatment significant?	YES: Yes<br>I DON'T KNOW:<br>NO:
	8. Was this effect accurate?	YES:<br>I DON'T KNOW: Not explained<br>NO:
	C) ARE THE RESULTS APPLICABLE IN YOUR ENVIRONMENT?	
	9. Can these results be applied to your local environment or population?	YES: Yes<br>I DON'T KNOW:<br>NO:
	10. Were all clinically relevant results taken into account?	YES: Yes<br>I DON'T KNOW:<br>NO:
	11. Do the benefits outweigh the risks and costs?	YES: Yes<br>I DON'T KNOW:<br>NO:
CASP score	10/11	

STUDY NUMBER: 21		
TITLE: **Zataria multiflora affects pulmonary function tests, respiratory symptoms, bronchodilator drugs use and hematological parameters in chronic obstructive pulmonary disease patients: A randomized doubled-blind clinical trial.**		
AUTHORS; YEAR OF PUBLICATION	Ghorani V, Rajabi O, Mirsadraee M, et al.<br>2024	
ARTICLE IDENTIFIERS	J Ethnopharmacol. 2024 May 23;326:117928. doi: 10.1016/j.jep.2024.117928. Epub 2024 Feb 17. PMID: 38373666.	
TYPE OF STUDY	A randomized doubled-blind clinical trial.	
PARTICIPANTS (P)	Group 1: patients (n = 14) received placebo (P). Group 2: patients (n = 14) received Z. multiflora extract 3 mg/kg/day (Z3).<br>Group 3: patients (n = 17) received Z. multiflora extract 6 mg/kg/day (Z6).	
INTERVENTION (I)	Administration of adjunctive treatment to the usual treatment for COPD with placebo, 3mg/kg, or 6mg/kg of Z. multiflora.	
COMPARISON (C)	Among groups.	
RESULTS (O)	COPD patients showed a significant decrease in respiratory symptoms, use of inhaled bronchodilators, total leukocyte and neutrophil counts, as well as an improvement in FEV1 values. These results, as well as those from previous studies, suggest that the improvement in COPD patients treated with Z. multiflora could be due to the anti-inflammatory effects of this plant. Therefore, Z. multiflora could be a potentially valuable herb for the treatment of chronic inflammatory disorders such as COPD.	
QUALITY OF THE ARTICLE	A) ARE THE TRIAL RESULTS VALID? (elimination questions; only if the first two questions are answered “yes” is it worth continuing to answer)	
	1. Is the trial focused on a clearly defined question?	YES: Yes<br>I DON'T KNOW:<br>NO:
	2. Was the allocation of patients to treatments random?	YES: Yes<br>I DON'T KNOW:<br>NO:
	3. Were all patients who entered the study adequately considered until the end of the study?	YES: Yes<br>I DON'T KNOW:<br>NO:
	“DETAIL” QUESTIONS	
	4. Was blinding maintained for:<br>• Patients<br>• Clinicians<br>• Study staff?	YES: Yes<br>I DON'T KNOW:<br>NO:
	5. Were the groups similar at the start of the trial?	YES: Yes<br>I DON'T KNOW:<br>NO:
	6. Apart from the intervention under study, were the groups treated equally?	YES: Yes<br>I DON'T KNOW:<br>NO:

	B) WHAT ARE THE RESULTS?	
	7. Is the effect of the treatment significant?	YES: Yes<br>I DON'T KNOW:<br>NO:
	8. Was this effect accurate?	YES: Yes<br>I DON'T KNOW:<br>NO:
	C) ARE THE RESULTS APPLICABLE IN YOUR ENVIRONMENT?	
	9. Can these results be applied to your local environment or population?	YES: Yes<br>I DON'T KNOW:<br>NO:
	10. Were all clinically relevant results taken into account?	YES: Yes<br>I DON'T KNOW:<br>NO:
	11. Do the benefits outweigh the risks and costs?	YES: Yes<br>I DON'T KNOW:<br>NO:
CASP score	11/11	

STUDY NUMBER: 22		
TITLE: **Efficacy and safety of dietary polyphenol supplements for COPD: a systematic review and meta-analysis.**		
AUTHORS; YEAR OF PUBLICATION	Wu D, Dong Y, Zhang D,<br>2022	
ARTICLE IDENTIFIERS	Front Immunol. 2025 Jul 23;16:1617694. doi: 10.3389/fimmu.2025.1617694. PMID: 40771814; PMCID: PMC12325041.	
TYPE OF STUDY	Systematic review and meta-analysis	
PARTICIPANTS (P)	COPD patients	
INTERVENTION (I)	Dietary polyphenols: (1) single active ingredients (e.g., anthocyanins, curcumin, and salvia polyphenols); (2) nutritional supplements fortified with polyphenols; (3) plant-derived polyphenol extracts (e.g., adjunctive treatment with oral AKL1(Activin receptor-like kinase 1).<br>The RCTs focused on this study involved dietary supplementation with 8 polyphenols: curcumin, resveratrol, anthocyanins, quercetin, salidroside, dietary beetroot juice, pomegranate juice and adjunctive treatment with oral AKL1.	
COMPARISON (C)	Placebo group	
RESULTS (O)	The results show that most of the dietary polyphenol components included can be used safely and have significant efficacy in relieving clinical symptoms, restoring respiratory function, and inhibiting inflammatory responses.	
QUALITY OF THE ARTICLE	A) ARE THE TRIAL RESULTS VALID? (elimination questions; only if the first two questions are answered "yes" is it worth continuing to answer)	
	1. Is the trial focused on a clearly defined question?	YES: Yes<br>I DON'T KNOW:<br>NO:
	2. Was the allocation of patients to treatments random?	YES: Yes<br>I DON'T KNOW:<br>NO:
	3. Were all patients who entered the study adequately considered until the end of the study?	YES: Yes<br>I DON'T KNOW:<br>NO:
	"DETAIL" QUESTIONS	
	4. Was blinding maintained for:<br>• Patients<br>• Clinicians<br>• Study staff?	YES: Yes<br>I DON'T KNOW:<br>NO:
	5. Were the groups similar at the start of the trial?	YES: Yes<br>I DON'T KNOW:<br>NO:
	6. Apart from the intervention under study, were the groups treated equally?	YES: Yes<br>I DON'T KNOW:<br>NO:

	B) WHAT ARE THE RESULTS?	
	7. Is the effect of the treatment significant?	YES: Yes<br>I DON'T KNOW:<br>NO:
	8. Was this effect accurate?	YES: Yes<br>I DON'T KNOW:<br>NO:
	C) ARE THE RESULTS APPLICABLE IN YOUR ENVIRONMENT?	
	9. Can these results be applied to your local environment or population?	YES: Yes<br>I DON'T KNOW:<br>NO:
	10. Were all clinically relevant results taken into account?	YES: Yes<br>I DON'T KNOW:<br>NO:
	11. Do the benefits outweigh the risks and costs?	YES: Yes<br>I DON'T KNOW:<br>NO:
CASP score	11/11	
