# Supplementary material for: Antioxidants as Therapeutic Tools in the Management of COPD: A Systematic Review with Meta-Analysis
Source: Antioxidants (Basel). 2026 Apr 2;15(4):446. doi: 10.3390/antiox15040446 (PMC13113252; doi:10.3390/antiox15040446)
Supplement: Supplementary file 1 [file antioxidants-15-00446-s001.zip › Supplementary Table S5.pdf]

<table>
<tr><td colspan="3">STUDY NUMBER: 1</td></tr>
<tr><td colspan="3">TITLE: A randomized, double-blind, placebo-controlled study evaluating the efficacy of propolis and N-acetylcysteine in exacerbations of chronic obstructive pulmonary disease.</td></tr>
<tr><td>AUTHORS; YEAR OF PUBLICATION</td><td colspan="2">I. Buha, M. Mirić, A. Agić, M. Simić, M. Stjepanović, B. Milenković, L. Nagorni-Obradović, V. Škodrić-Trifunović, B. Ilić, S. Popević, S. Dimic-Janjic, A. Ilić.
2022.</td></tr>
<tr><td>ARTICLE IDENTIFIERS</td><td colspan="2">Eur Rev Med Pharmacol Sci. 2022 Jul;26(13):4809-4815. doi: 10.26355/eurrev_202207_29206. PMID: 35856373.</td></tr>
<tr><td>TYPE OF STUDY</td><td colspan="2">Randomized clinical trial: Single-center, randomized, double-blind, phase IV trial with three treatment arms.</td></tr>
<tr><td>PARTICIPANTS (P)</td><td colspan="2">46 patients diagnosed with exacerbation of COPD.</td></tr>
<tr><td>INTERVENTION (I)</td><td colspan="2">The study involved the administration of a formulation containing 600 mg of N-acetylcysteine (NAC) and 80 mg of propolis to two treatment groups. In addition, a second group was administered 1200 mg of NAC and 160 mg of propolis. The treatment frequency is as follows: the study drug is to be administered once-daily for a period of three months, with a subsequent one-year follow-up period.
The primary endpoint of the study is as follows: The following investigation will ascertain the incidence rate of COPD exacerbation during the follow-up period in each group of treatment.</td></tr>
<tr><td>COMPARISON (C)</td><td colspan="2">COPD patients that received placebo.</td></tr>
<tr><td>RESULTS (O)</td><td colspan="2">A statistically significant difference in the incidence rate of acute exacerbation of COPD was observed: The placebo arm recorded a 52.6% response rate, while the AS-600 arm achieved 15.4%. Notably, the AS-1,200 arm exhibited a response rate of 7.1%, a finding that survived the Fisher's exact test (p = 0.013). A comparison of the frequency of acute exacerbations of COPD between the AS-1,200 group and the placebo group revealed a statistically significant difference in favour of the former (p = 0.009). When compared with a placebo, the relative risk of exacerbation was 0.29 in AS-600 and 0.13 in AS-1,200. No treatment-related adverse events were reported.
Conclusions: The oral combination of natural propolis with NAC has been demonstrated to be both efficacious and safe. The findings of this study require validation through the execution of additional clinical trials of a larger scale.</td></tr>
<tr><td rowspan="4">QUALITY OF THE ARTICLE</td><td colspan="2">A) ARE THE TRIAL RESULTS VALID? (elimination questions; only if the first two questions are answered “yes” is it worth continuing to answer)</td></tr>
<tr><td>1. Is the trial focused on a clearly defined question?</td><td>YES: Verify that the intervention is correct.
I DON'T KNOW:
NO:</td></tr>
<tr><td>1. Was the allocation of patients to treatments random?</td><td>YES: Due to the double-blind design.
I DON'T KNOW:
NO:</td></tr>
<tr><td>2. Were all patients who entered the study adequately considered until the end of the study?</td><td>YES: Due to the double-blind design.
I DON'T KNOW:
NO:</td></tr>
</table>

	"DETAIL" QUESTIONS	
	**3.** Was blinding maintained for:<br>• Patients<br>• Clinicians<br>• Study staff?	YES: Yes<br>I DON'T KNOW:<br>NO:
	**4.** Were the groups similar at the start of the trial?	YES: Yes<br>I DON'T KNOW:<br>NO:
	**5.** Apart from the intervention under study, were the groups treated equally?	YES: Yes<br>I DON'T KNOW:<br>NO:
	B) WHAT ARE THE RESULTS?	
	**6.** Is the effect of the treatment significant?	YES: Compared with placebo, the frequency of acute exacerbations of COPD was significantly lower only in AS-1.200 ($p = 0.009$).<br>I DON'T KNOW:<br>NO:
	**7.** Was this effect accurate?	YES: It is a statistically significant effect.<br>I DON'T KNOW:<br>NO:
	C) ARE THE RESULTS APPLICABLE IN YOUR ENVIRONMENT?	
	**8.** Can these results be applied to your local environment or population?	YES: Yes.<br>I DON'T KNOW:<br>NO:
	**9.** Were all clinically relevant results taken into account?	YES: Yes<br>I DON'T KNOW:<br>NO:
	**10.** Do the benefits outweigh the risks and costs?	YES: There was no risk involved in the procedure.<br>I DON'T KNOW:<br>NO:
CASP score	11/11	

STUDY NUMBER: 2		
TITLE: A Randomized, Doubled-Blind Clinical Trial on the Effect of *Zataria multiflora* on Clinical Symptoms, Oxidative Stress, and C-Reactive Protein in COPD Patients		
AUTHORS; YEAR OF PUBLICATION	Vahideh Ghorani, Omid Rajabi, Majid Mirsadraee, Fariba Rezaeitalab, Saeideh Saadat and Mohammad Hossein Boskabady.<br>2020.	
ARTICLE IDENTIFIERS	J Clin Pharmacol. 2020 Jul;60(7):867-878. doi: 10.1002/jcph.1586. Epub 2020 Mar 23. PMID: 32202330.	
TYPE OF STUDY	Randomized clinical trial, double-blind.	
PARTICIPANTS (P)	45 patients with COPD (GOLD II) aged 16-60 years	
INTERVENTION (I)	Administration of *Z. multiflora* extract 3 mg/kg/day, compared with 6 mg/kg/day of *Z. multiflora* extract and placebo as adjuvant therapy to salbutamol and corticosteroids.<br>Evaluation of clinical and OS and C-reactive protein parameters were evaluated as follows: pretreatment (Step 0) and 1 (Step I) and 2 (Step II) months after treatment.	
COMPARISON (C)	COPD patients fulfilling the eligibility criteria that received placebo.	
RESULTS (O)	The clinical symptoms, including dyspnoea and wheezing in the Z3 and Z6 treatment groups, and sputum production in the Z6 treatment group alone, exhibited a significant improvement one to two months after treatment in comparison with the initial values (P < 0.01 to P < 0.001). Following a two-month period of treatment with Z3 and Z6, a significant increase in FEV1 was observed (P < 0.05 to P < 0.01). Levels of malondialdehyde and nitrite decreased significantly after two months of treatment with Z6 in comparison with the initial levels (P < 0.05 to P < 0.01). As can be seen from the data presented in Figure 1, there was a significant increase in thiol content in the Z6 group, as well as in the superoxide dismutase and catalase activities of both groups treated with the extract, at step II compared to step 0 (P < 0.05 to P < 0.01). The level of C-reactive protein at the conclusion of the study was found to be significantly reduced in both treated groups in comparison to step 0 (P < 0.05 in both cases). Treatment with Z. multiflora for a period of two months resulted in an improvement in clinical symptoms, pulmonary function tests, oxidative stress, and C-reactive protein levels in patients diagnosed with COPD.<br>Conclusions: The results of this study indicate the potential therapeutic value of this herbal medicine in the prevention and treatment of COPD, warranting further research to explore its therapeutic effects in more detail.	
QUALITY OF THE ARTICLE	A) ARE THE TRIAL RESULTS VALID? (elimination questions; only if the first two questions are answered “yes” is it worth continuing to answer)	
	1. Is the trial focused on a clearly defined question?	YES: Yes<br>I DON'T KNOW:<br>NO:
	2. Was the allocation of patients to treatments random?	YES: Yes, a randomized double blind clinical trial.<br>I DON'T KNOW:<br>NO:
	3. Were all patients who entered the study adequately considered until the end of the study?	YES: Yes. Only patients that fulfilled eligibility criteria and completed the entire procedure were included in the

		study.<br>I DON'T KNOW:<br>NO:
	"DETAIL" QUESTIONS	
	4. Was blinding maintained for:<br>• Patients<br>• Clinicians<br>• Study staff?	YES: Yes<br>I DON'T KNOW:<br>NO:
	5. Were the groups similar at the start of the trial?	YES: Yes<br>I DON'T KNOW:<br>NO:
	6. Apart from the intervention under study, were the groups treated equally?	YES: Yes<br>I DON'T KNOW:<br>NO:
	B) WHAT ARE THE RESULTS?	
	7. Is the effect of the treatment significant?	YES: Yes. Two-month treatment with Z. multiflora significantly improved clinical symptoms, pulmonary function tests, oxidative stress, and C-reactive protein in COPD patients.<br>I DON'T KNOW:<br>NO:
	8. Was this effect accurate?	YES:<br>I DON'T KNOW: Not explained, although results are statistically significant.<br>NO:
	C) ARE THE RESULTS APPLICABLE IN YOUR ENVIRONMENT?	
	9. Can these results be applied to your local environment or population?	YES: Yes.<br>I DON'T KNOW:<br>NO:
	10. Were all clinically relevant results taken into account?	YES: Yes<br>I DON'T KNOW:<br>NO:
	11. Do the benefits outweigh the risks and costs?	YES: Yes. According to the authors and the results "The results suggest that this herbal medicine could be of therapeutic value as a preventive drug for the treatment of COPD."<br>I DON'T KNOW:<br>NO:
CASP score	9/11	

STUDY NUMBER: 3	
TITLE: Changes in Blood Markers of Oxidative Stress, Inflammation and Cardiometabolic Patients with COPD after Eccentric and Concentric Cycling Training.	
AUTHORS; YEAR OF PUBLICATION	Mayalen Valero-Breton, Denisse Valladares-Ide, Cristian Álvarez, Reyna S Peñailillo, Luis Peñailillo.<br>2023.
ARTICLE IDENTIFIERS	Nutrients. 2023 Feb 11;15(4):908. doi: 10.3390/nu15040908. PMID: 36839267; PMCID: PMC9966444.
TYPE OF STUDY	Prospective randomized training study.
PARTICIPANTS (P)	20 participants with moderate COPD (GOLD II).
INTERVENTION (I)	The present study examined the effects of eccentric (ECC) and concentric (CONC) bicycle training over a 12-week period on plasma markers of cardiometabolic health, oxidative stress, and inflammation in patients with COPD. A randomized controlled trial was conducted in which patients with moderate COPD were assigned to the ECC (n = 10; mean age ± standard deviation (SD): 68.2 ± 10.0 years) or CONC (n = 10; mean age ± SD: 71.1 ± 10.3 years) training groups. The participants underwent 12 weeks of either ECC or CONC training, with 2-3 sessions per week and 10-30 minutes per session. Prior to and following the training period, a series of assessments were conducted to ascertain the maximum oxygen consumption, maximum power output (VO2peak and POmáx), and time to exhaustion (TTE). The plasma antioxidant and oxidative markers, insulin resistance, lipid profile, and systemic inflammation markers were measured before and after training at rest.
COMPARISON (C)	COPD patients using eccentric (ECC) and concentric (CONC) bicycle training. Comparison of both groups with respect to respective baseline values.
RESULTS (O)	Peak VO2, POmax, and TTE remained unchanged after ECC and CONC. CONC induced an increase in antioxidants ($p = 0.01$), while ECC decreased antioxidant markers ($p = 0.02$) measured at rest. CONC induced a lower increase in oxidative stress after ECC ($p = 0.04$) and a decrease in insulin resistance ($p = 0.0006$) compared to baseline.<br>Conclusion: The results of this study indicate that CONC training resulted in an enhancement of insulin sensitivity, an increase in antioxidant capacity during rest, and a reduction in exercise-induced oxidative stress in patients diagnosed with moderate COPD.
QUALITY OF THE ARTICLE	A) ARE THE TRIAL RESULTS VALID? (elimination questions; only if the first two questions are answered "yes" is it worth continuing to answer)

Question	Answer
1. Is the trial focused on a clearly defined question?	YES: Yes<br>I DON'T KNOW:<br>NO:
2. Was the allocation of patients to treatments random?	YES: Yes, it is a randomized study.<br>I DON'T KNOW:<br>NO:
3. Were all patients who entered the study adequately considered until the end of the study?	YES: Yes<br>I DON'T KNOW:<br>NO:

"DETAIL" QUESTIONS

	4. Was blinding maintained for:<br>• Patients<br>• Clinicians<br>• Study staff?	YES:<br>I DON'T KNOW:<br>NO: No
	5. Were the groups similar at the start of the trial?	YES: Yes. The study was randomized and there was stratification of patients.<br>I DON'T KNOW:<br>NO:
	6. Apart from the intervention under study, were the groups treated equally?	YES: Yes<br>I DON'T KNOW:<br>NO:
	B) WHAT ARE THE RESULTS?	
	7. Is the effect of the treatment significant?	YES: Yes, results in the CONC group were statistically significant.<br>I DON'T KNOW:<br>NO:
	8. Was this effect accurate?	YES:<br>I DON'T KNOW: Precision estimators of the effect have not been measured, but there are statistically significant results.<br>NO:
	C) ARE THE RESULTS APPLICABLE IN YOUR ENVIRONMENT?	
	9. Can these results be applied to your local environment or population?	YES: Yes<br>I DON'T KNOW:<br>NO:
	10. Were all clinically relevant results taken into account?	YES: Yes<br>I DON'T KNOW:<br>NO:
	11. Do the benefits outweigh the risks and costs?	YES: Yes.<br>I DON'T KNOW:<br>NO:
CASP score	9/11	

STUDY NUMBER: 4		
TITLE: Dietary nitrate supplementation to enhance exercise capacity in hypoxic COPD: EDEN-OX, a double-blind, placebo-controlled, randomised cross-over study.		
AUTHORS; YEAR OF PUBLICATION	Matthew J Pavitt, Adam Lewis, Sara C Buttery, Bernadette O Fernandez, Monika Mikus-Lelinska, Winston A S Banya, Martin Feelisch, Michael I Polkey, Nicholas S Hopkinson.<br>2021.	
ARTICLE IDENTIFIERS	Thorax. 2022 Oct;77(10):968-975. doi: 10.1136/thoraxjnl-2021-217147. Epub 2021 Dec 1. PMID: 34853156.	
TYPE OF STUDY	Single-centre, double-blind, placebo-controlled, cross-over study.	
PARTICIPANTS (P)	20 COPD patients who were established users of long-term oxygen therapy.	
INTERVENTION (I)	Compare the effects of dietary supplementation with NO3- (nitrate-rich beet juice, NR-BRJ) with a matched placebo in individuals with COPD who require long-term oxygen therapy (LTOT) and use ambulatory oxygen therapy (AOT) during exercise.	
COMPARISON (C)	COPD patients fulfilling the eligibility criteria that received placebo.	
RESULTS (O)	The supplementation of beet juice (NR-BRJ) rich in nitrate resulted in a significant increase in the duration of exercise endurance for all participants when compared to the placebo. The median (interquartile range) duration of exercise for the former was 194.6 (147.5-411.7) seconds, while for the latter it was 159.1 (121.9-298.5) seconds. The estimated treatment effect was 62 (33-106) seconds (p<0.0001). Supplementation also improved endothelial function: the nitrate-rich beet juice (NR-BRJ) group showed an increase of 4.1% (from -1.1% to 14.8%), while the placebo beet juice (BRJ) group showed a decrease of 5.0% (from -10.6% to -0.6%) (p=0.0003).<br>Conclusion: It has been demonstrated that acute dietary nitrate supplementation increases exercise endurance in patients with COPD who require supplemental oxygen.	
QUALITY OF THE ARTICLE	A) ARE THE TRIAL RESULTS VALID? (elimination questions; only if the first two questions are answered “yes” is it worth continuing to answer)	
	1. Is the trial focused on a clearly defined question?	YES: Yes<br>I DON'T KNOW:<br>NO:
	2. Was the allocation of patients to treatments random?	YES: Yes. Treatment order was allocated (1:1) by computer-generated block randomisation.<br>I DON'T KNOW:<br>NO:
	3. Were all patients who entered the study adequately considered until the end of the study?	YES: Yes<br>I DON'T KNOW:<br>NO:
	“DETAIL” QUESTIONS	
	4. Was blinding maintained for:<br>• Patients<br>• Clinicians	YES: Yes<br>I DON'T KNOW:<br>NO:

	• Study staff?	
	5. Were the groups similar at the start of the trial?	YES: Yes<br>I DON'T KNOW:<br>NO:
	6. Apart from the intervention under study, were the groups treated equally?	YES: Yes<br>I DON'T KNOW:<br>NO:
	B) WHAT ARE THE RESULTS?	
	7. Is the effect of the treatment significant?	YES: Yes<br>I DON'T KNOW:<br>NO:
	8. Was this effect accurate?	YES:<br>I DON'T KNOW: Not explained.<br>NO:
	C) ARE THE RESULTS APPLICABLE IN YOUR ENVIRONMENT?	
	9. Can these results be applied to your local environment or population?	YES: Yes<br>I DON'T KNOW:<br>NO:
	10. Were all clinically relevant results taken into account?	YES: Yes<br>I DON'T KNOW:<br>NO:
	11. Do the benefits outweigh the risks and costs?	YES: Yes<br>I DON'T KNOW:<br>NO:
CASP score	10/11	

STUDY NUMBER: 5		
TITLE: Effect of Crocin From Saffron (Crocus sativus L.) Supplementation on Oxidant/Antioxidant Markers, Exercise Capacity, and Pulmonary Function Tests in COPD Patients: A Randomized, Double-Blind, Placebo-Controlled Trial.		
AUTHORS; YEAR OF PUBLICATION	Hassan Ghobadi, Nasim Abdollahi, Hanieh Madani y Mohammad Reza Aslani. 2022.	
ARTICLE IDENTIFIERS	Front Pharmacol. 2022 Apr 20;13:884710. doi: 10.3389/fphar.2022.884710. PMID: 35517806; PMCID: PMC9065288.	
TYPE OF STUDY	Randomized, double-blind, placebo-controlled clinical trial.	
PARTICIPANTS (P)	46 patients, 23 in the placebo group and 23 in the intervention group.	
INTERVENTION (I)	The intervention group received crocin at a concentration of 30 mg/day for 12 weeks, while the control group received a placebo.	
COMPARISON (C)	COPD patients treated with placebo.	
RESULTS (O)	Twelve weeks of crocin treatment in patients diagnosed with Chronic Obstructive Pulmonary Disease (COPD) resulted in a decrease in serum levels of total oxidant status (TOS) and NF-κB, alongside an increase in total antioxidant capacity (TAOC). Furthermore, the 6-minute walk test (6MWT) has been demonstrated to reveal an enhancement in patients' exercise capacity.<br>Crocin supplementation has been demonstrated to effectively establish oxidant/antioxidant balance. Furthermore, it has been demonstrated to ameliorate inflammatory conditions in patients diagnosed with COPD.<br>Conclusion: Crocin supplementation has been demonstrated to effectively establish oxidant/antioxidant balance and improve inflammatory conditions in patients with COPD.	
QUALITY OF THE ARTICLE	A) ARE THE TRIAL RESULTS VALID? (elimination questions; only if the first two questions are answered "yes" is it worth continuing to answer)	
	1. Is the trial focused on a clearly defined question?	YES: Yes<br>I DON'T KNOW:<br>NO:
	2. Was the allocation of patients to treatments random?	YES: Yes<br>I DON'T KNOW:<br>NO:
	3. Were all patients who entered the study adequately considered until the end of the study?	YES: Yes<br>I DON'T KNOW:<br>NO:
	"DETAIL" QUESTIONS	
	4. Was blinding maintained for:<br>• Patients<br>• Clinicians<br>• Study staff?	YES: Yes. Double-blind.<br>I DON'T KNOW:<br>NO:
	5. Were the groups similar at the start of the trial?	YES: Yes<br>I DON'T KNOW:

		NO:
	6. Apart from the intervention under study, were the groups treated equally?	YES: Yes<br>I DON'T KNOW:<br>NO:
	B) WHAT ARE THE RESULTS?	
	7. Is the effect of the treatment significant?	YES: Yes, although some results didn´t show statistical significance.<br>I DON'T KNOW:<br>NO:
	8. Was this effect accurate?	YES:<br>I DON'T KNOW: Not explained<br>NO:
	C) ARE THE RESULTS APPLICABLE IN YOUR ENVIRONMENT?	
	9. Can these results be applied to your local environment or population?	YES: Yes<br>I DON'T KNOW:<br>NO:
	10. Were all clinically relevant results taken into account?	YES: Yes<br>I DON'T KNOW:<br>NO:
	11. Do the benefits outweigh the risks and costs?	YES: Yes<br>I DON'T KNOW:<br>NO:
CASP score	10/11	

STUDY NUMBER: 6		
TITLE: Effect of crocin of *Crocus sativus* L. on serum inflammatory markers (IL-6 and TNF-α) in chronic obstructive pulmonary disease patients: a randomised, double-blind, placebo-controlled trial		
AUTHORS; YEAR OF PUBLICATION	Mohammad Reza Aslani, Nasim Abdollahi, Somaieh Matin, Anahita Zakeri and Hassan Ghobadi.<br>2023.	
ARTICLE IDENTIFIERS	Br J Nutr. 2023 Aug 14;130(3):446-453. doi: 10.1017/S0007114522003397.	
TYPE OF STUDY	Randomized, double-blind, placebo-controlled trial	
PARTICIPANTS (P)	57 male patients divided equally between two groups	
INTERVENTION (I)	The intervention and control groups received 15 mg of Krocina™ tablets twice daily (a product of the Buali Research Institute of Pharmaceutical Sciences, Pharmaceutical Product Development Center, Sina Pooyesh Drug Company, registration number 48674, and IRC number: 0648126930388841) and placebo for a period of 12 weeks. The placebo compounds utilised in this study were Avicel, polyvinylpyrrolidone, and magnesium stearate. The administration of the crocin/placebo tablets occurred in two doses, at the commencement and the end of each day, in conjunction with the main meals (breakfast and dinner) for a period of 12 weeks.	
COMPARISON (C)	COPD patients treated with placebo.	
RESULTS (O)	Crocine has been demonstrated to enhance pulmonary function test (PFT) results ($p < 0.05$) and 6-minute walk distance (6-MWD) test results ($p < 0.001$). Furthermore, it has been shown to exert a preventive effect by increasing serum IL-6 levels in COPD patients in comparison to those in the placebo group ($p < 0.05$). The intervention of crocine resulted in a significant reduction in serum TNF-α levels at the conclusion of the study ($p<0.01$).<br>The results of this study indicate that crocine supplementation enhances exercise capacity and improves peripheral lung function in COPD patients by reducing serum levels of inflammatory factors.<br>Conclusion: The present findings suggest that crocin supplementation improves exercise capacity and PFT in patients with COPD by reducing serum levels of inflammatory factors.	
QUALITY OF THE ARTICLE	A) ARE THE TRIAL RESULTS VALID? (elimination questions; only if the first two questions are answered “yes” is it worth continuing to answer)	
	2. Is the trial focused on a clearly defined question?	YES: Yes<br>I DON'T KNOW:<br>NO:
	2. Was the allocation of patients to treatments random?	YES: Yes.<br>I DON'T KNOW:<br>NO:
	3. Were all patients who entered the study adequately considered until the end of the study?	YES: Yes<br>I DON'T KNOW:<br>NO:
	“DETAIL” QUESTIONS	

	4. Was blinding maintained for:<br>• Patients<br>• Clinicians<br>• Study staff?	YES: Yes<br>I DON'T KNOW:<br>NO:
	5. Were the groups similar at the start of the trial?	YES: Yes<br>I DON'T KNOW:<br>NO:
	6. Apart from the intervention under study, were the groups treated equally?	YES: Yes<br>I DON'T KNOW:<br>NO:
	B) WHAT ARE THE RESULTS?	
	7. Is the effect of the treatment significant?	YES: Yes<br>I DON'T KNOW:<br>NO:
	8. Was this effect accurate?	YES: Yes<br>I DON'T KNOW:<br>NO:
	C) ARE THE RESULTS APPLICABLE IN YOUR ENVIRONMENT?	
	9. Can these results be applied to your local environment or population?	YES: Yes<br>I DON'T KNOW:<br>NO:
	10. Were all clinically relevant results taken into account?	YES: Yes<br>I DON'T KNOW:<br>NO:
	11. Do the benefits outweigh the risks and costs?	YES: Yes<br>I DON'T KNOW:<br>NO:
CASP score	11/11	

<table>
<tr><td colspan="3">STUDY NUMBER: 7</td></tr>
<tr><td colspan="3">TITLE: Effect of high-dose N-acetylcysteine on exacerbations and lung function in patients with mild-to-moderate COPD: a double blind, parallel group, multicentre randomised clinical trial.</td></tr>
<tr><td>AUTHORS; YEAR OF PUBLICATION</td><td colspan="2">Yumin Zhou, Fan Wu, Zhe Shi, Jie Cao, Jia Tian, Weimin Yao, Liping Wei, Fenglei Li, Shan Cai, Yao Shen, Zanfeng Wang, Huilan Zhang, Yanfan Chen, Yingyun Fu, Zhiyi He, Chun Chang, Yongliang Jiang, Shujing Chen, Changli Yang, Shuqing Yu, Heshen Tian, Qijian Cheng, Ziwen Zhao, Yinghua Ying, Yong Zhou, Shengming Liu, Zhishan Deng, Peiyu Huang, Yunzhen Zhang, Xiangwen Luo, Haiyan Zhao, Jianping Gui, Weiguang Lai, Guoping Hu, Cong Liu, Ling Su, Zhiguang Liu, Jianhui Huang, Dongxing Zhao, Nanshan Zhong & Pixin Ran.
2024.</td></tr>
<tr><td>ARTICLE IDENTIFIERS</td><td colspan="2">Nat Commun. 2024 Sep 30;15(1):8468. doi: 10.1038/s41467-024-51079-1. PMID: 39349461; PMCID: PMC11442465.</td></tr>
<tr><td>TYPE OF STUDY</td><td colspan="2">Multicenter, randomized, double-blind, placebo-controlled trial.</td></tr>
<tr><td>PARTICIPANTS (P)</td><td colspan="2">924 patients with mild to moderate COPD completed the two-year follow-up (placebo group: n = 460; N-acetylcysteine group: n = 464).</td></tr>
<tr><td>INTERVENTION (I)</td><td colspan="2">Patients with mild to moderate COPD were randomized 1:1 to receive high doses of N-acetylcysteine (600 mg, twice daily) or placebo (twice daily) for two years.</td></tr>
<tr><td>COMPARISON (C)</td><td colspan="2">COPD patients treated with placebo.</td></tr>
<tr><td>RESULTS (O)</td><td colspan="2">The difference between the N-acetylcysteine group and the placebo group in the annual rate of total exacerbations was not significant (0.65 vs. 0.72 per patient-year; relative risk (RR), 0.90; 95% confidence interval (CI), 0.80-1.02; p = 0.10). No significant differences in FEV1 were observed prior to bronchodilator administration at 24 months. The present study examined the effects of long-term treatment with high doses of N-acetylcysteine on the annual rate of total exacerbations and lung function in patients diagnosed with mild to moderate COPD. The results indicated that the treatment did not result in a significant reduction in the annual rate of total exacerbations or improvement in lung function.</td></tr>
<tr><td rowspan="6">QUALITY OF THE ARTICLE</td><td colspan="2">A) ARE THE TRIAL RESULTS VALID? (elimination questions; only if the first two questions are answered “yes” is it worth continuing to answer)</td></tr>
<tr><td>3. Is the trial focused on a clearly defined question?</td><td>YES: Yes
I DON'T KNOW:
NO:</td></tr>
<tr><td>2. Was the allocation of patients to treatments random?</td><td>YES: Yes
I DON'T KNOW:
NO:</td></tr>
<tr><td>3. Were all patients who entered the study adequately considered until the end of the study?</td><td>YES: Yes
I DON'T KNOW:
NO:</td></tr>
<tr><td colspan="2">“DETAIL” QUESTIONS</td></tr>
<tr><td>4. Was blinding maintained for:
• Patients
• Clinicians
• Study staff?</td><td>YES: Yes, double-blind
I DON'T KNOW:
NO:</td></tr>
</table>

	5. Were the groups similar at the start of the trial?	YES: Yes<br>I DON'T KNOW:<br>NO:
	6. Apart from the intervention under study, were the groups treated equally?	YES: Yes<br>I DON'T KNOW:<br>NO:
	B) WHAT ARE THE RESULTS?	
	7. Is the effect of the treatment significant?	YES:<br>I DON'T KNOW:<br>NO: No
	8. Was this effect accurate?	YES:<br>I DON'T KNOW: Not explained<br>NO:
	C) ARE THE RESULTS APPLICABLE IN YOUR ENVIRONMENT?	
	9. Can these results be applied to your local environment or population?	YES: Yes<br>I DON'T KNOW:<br>NO:
	10. Were all clinically relevant results taken into account?	YES: Yes<br>I DON'T KNOW:<br>NO:
	11. Do the benefits outweigh the risks and costs?	YES: Yes<br>I DON'T KNOW:<br>NO:
CASP score	10/11	

<table>
<tr><td colspan="3">STUDY NUMBER: 8</td></tr>
<tr><td colspan="3">TITLE: Effects of antioxidant nutrients on muscle mass, strength and function in COPD patients: A meta-analysis of randomized controlled trials</td></tr>
<tr><td>AUTHORS; YEAR OF PUBLICATION</td><td colspan="2">Qinman He, Pan Yang, Ye Wang, Wanmei Xu, Yi Feng, Fei Xie, Guixiang Xu.
2025.</td></tr>
<tr><td>ARTICLE IDENTIFIERS</td><td colspan="2">PLoS One. 2025 Jan 17;20(1):e0316842. doi: 10.1371/journal.pone.0316842. PMID: 39823472; PMCID: PMC11741611.</td></tr>
<tr><td>TYPE OF STUDY</td><td colspan="2">Systematic review and meta-analysis.</td></tr>
<tr><td>PARTICIPANTS (P)</td><td colspan="2">A total of 12 studies involving 595 COPD patients were included, with 296 patients in the antioxidant nutrient intervention group and 299 in the non-antioxidant nutrient intervention group.</td></tr>
<tr><td>INTERVENTION (I)</td><td colspan="2">Thoroughly investigate the effects of antioxidant nutrients on muscle mass, strength, and function in patients with chronic obstructive pulmonary disease (COPD).</td></tr>
<tr><td>COMPARISON (C)</td><td colspan="2">COPD patients do not taking antioxidant treatment.</td></tr>
<tr><td>RESULTS (O)</td><td colspan="2">With regard to muscle mass, patients who received antioxidant nutrients exhibited a significantly higher lean body mass index in comparison to those who did not receive antioxidant nutrients (pooled weighted mean differences [WMD]: 0.903; 95% CI: 0.264–1.541; p = 0.006). For patients who did not participate in the pulmonary rehabilitation programme while receiving nutritional interventions, antioxidant nutrients resulted in a significantly higher lean body mass index (pooled WMD: 1.360; 95% CI: 0.560–2.160; p = 0.001). With regard to muscle strength, patients in the antioxidant nutrient intervention group exhibited significantly greater handgrip strength (HGS) in comparison to those in the non-antioxidant nutrient intervention group (pooled SMD: 1.976; 95% CI: 1.337–2.615; P < 0.001). Patients who received antioxidant nutrients exhibited significantly greater inspiratory muscle strength (IMS) in comparison to those who did not receive antioxidant nutrients (pooled SMD: 8.127; 95% CI: 2.677–13.577; P = 0.003).
The administration of antioxidant nutrients resulted in a significant enhancement of hand grip strength (HGS), maximum inspiratory pressure (MIP), and lean body mass index in patients diagnosed with COPD. It is recommended that clinicians consider the incorporation of antioxidant-rich foods or supplements within the treatment plan for COPD patients.
Conclusion: The intervention, which entailed the administration of antioxidant nutrients, resulted in a significant improvement in health-related quality of life, as measured by HGS, MIP and lean body mass index, in patients diagnosed with COPD. It is recommended that clinicians consider increasing food intake or supplementation rich in antioxidants as part of the treatment plan for patients with COPD.</td></tr>
<tr><td rowspan="4">QUALITY OF THE ARTICLE</td><td colspan="2">A) ARE THE TRIAL RESULTS VALID? (elimination questions; only if the first two questions are answered “yes” is it worth continuing to answer)</td></tr>
<tr><td>4. Is the trial focused on a clearly defined question?</td><td>YES: Yes
I DON'T KNOW:
NO:</td></tr>
<tr><td>2. Was the allocation of patients to treatments random?</td><td>YES: Yes
I DON'T KNOW:
NO:</td></tr>
<tr><td>3. Were all patients who entered the study adequately considered until</td><td>YES: Yes
I DON'T KNOW:</td></tr>
</table>

	the end of the study?	NO:
	"DETAIL" QUESTIONS	
	4. Was blinding maintained for:<br>• Patients<br>• Clinicians<br>• Study staff?	YES: Yes<br>I DON'T KNOW:<br>NO:
	5. Were the groups similar at the start of the trial?	YES: Yes<br>I DON'T KNOW:<br>NO:
	6. Apart from the intervention under study, were the groups treated equally?	YES: Yes<br>I DON'T KNOW:<br>NO:
	B) WHAT ARE THE RESULTS?	
	7. Is the effect of the treatment significant?	YES: Yes<br>I DON'T KNOW:<br>NO:
	8. Was this effect accurate?	YES: Yes<br>I DON'T KNOW:<br>NO:
	C) ARE THE RESULTS APPLICABLE IN YOUR ENVIRONMENT?	
	9. Can these results be applied to your local environment or population?	YES: Yes<br>I DON'T KNOW:<br>NO:
	10. Were all clinically relevant results taken into account?	YES: Yes<br>I DON'T KNOW:<br>NO:
	11. Do the benefits outweigh the risks and costs?	YES: Yes<br>I DON'T KNOW:<br>NO:
CASP score	11/11	

<table>
<tr><td colspan="3">STUDY NUMBER: 9</td></tr>
<tr><td colspan="3">TITLE: Efficacy of 12 weeks oral beta-alanine supplementation in patients with chronic obstructive pulmonary disease: a double-blind, randomized, placebo-controlled trial.</td></tr>
<tr><td>AUTHORS; YEAR OF PUBLICATION</td><td colspan="2">Jana De Brandt, Wim Derave, Frank Vandenabeele, Pascal Pomiès, Laura Blancquaert, Charly Keytsman, Marina S. Barusso-Grüninger, Fabiano F. de Lima, Maurice Hayot, Martijn A. Spruit y Chris Burtin.
2022.</td></tr>
<tr><td>ARTICLE IDENTIFIERS</td><td colspan="2">J Cachexia Sarcopenia Muscle. 2022 Oct;13(5):2361-2372. doi: 10.1002/jcsm.13048. Epub 2022 Aug 17. PMID: 35977911; PMCID: PMC9530565.</td></tr>
<tr><td>TYPE OF STUDY</td><td colspan="2">Double-blind, randomized, placebo-controlled trial</td></tr>
<tr><td>PARTICIPANTS (P)</td><td colspan="2">40 COPD patients</td></tr>
<tr><td>INTERVENTION (I)</td><td colspan="2">After a 2-week baseline assessment, the intervention group received 12 weeks of sustained-release Beta-alanine (BA) supplementation (SR CarnoSyn®) consisting of an oral intake of 3.2 g/day of BA (four 800 mg/day tablets). The control group received maltodextrin as placebo supplementation.</td></tr>
<tr><td>COMPARISON (C)</td><td colspan="2">COPD patients receiving placebo.</td></tr>
<tr><td>RESULTS (O)</td><td colspan="2">Beta-alanine supplementation increased muscle carnosine levels in patients with COPD compared with placebo (mean difference [95% confidence interval]; +2.82 [1.49–4.14] mmol/kg wet weight; P < 0.001). The maximum incremental capacity for cycling (VO2peak: The range was found to be between 0.7 and 1.7 mL/kg/min, with a P-value of 0.384 and a Wpeak: The +5 [1 to 11] W; P = 0.103 result and the time to exhaustion in the constant work rate cycling test (+28 [179 to 236] s; P = 0.782) did not change significantly. It is evident that there was no significant difference in the adherence to supplement intake between the BA and PL groups (P = 0.294). Furthermore, no adverse effects were reported by patients that could be possibly linked to supplement intake.
Beta-alanine supplementation has been shown to effectively increase muscle carnosine levels by 54% from the mean baseline value in patients with COPD, without causing any side effects, when compared to placebo. However, no significant improvements in exercise capacity, quadriceps function or muscle oxidative/carbonyl stress were observed.
Conclusions: Beta-alanine supplementation has been shown to be effective in increasing muscle carnosine levels by 54% on average compared to the mean baseline value. These results have been achieved without any reported side effects in patients with COPD when compared to a placebo. However, there were no accompanying beneficial changes in exercise capacity, quadriceps function, or muscle oxidative/carbonyl stress.</td></tr>
<tr><td rowspan="3">QUALITY OF THE ARTICLE</td><td colspan="2">A) ARE THE TRIAL RESULTS VALID? (elimination questions; only if the first two questions are answered “yes” is it worth continuing to answer)</td></tr>
<tr><td>5. Is the trial focused on a clearly defined question?</td><td>YES: Yes
I DON'T KNOW:
NO:</td></tr>
<tr><td>2. Was the allocation of patients to treatments random?</td><td>YES: Yes
I DON'T KNOW:
NO:</td></tr>
</table>

	3. Were all patients who entered the study adequately considered until the end of the study?	YES: Yes<br>I DON'T KNOW:<br>NO:
	"DETAIL" QUESTIONS	
	4. Was blinding maintained for:<br>• Patients<br>• Clinicians<br>• Study staff?	YES: Yes<br>I DON'T KNOW:<br>NO:
	5. Were the groups similar at the start of the trial?	YES: Yes<br>I DON'T KNOW:<br>NO:
	6. Apart from the intervention under study, were the groups treated equally?	YES: Yes<br>I DON'T KNOW:<br>NO:
	B) WHAT ARE THE RESULTS?	
	7. Is the effect of the treatment significant?	YES: While some parameters showed significant differences, others did not.<br>I DON'T KNOW:<br>NO:
	8. Was this effect accurate?	YES:<br>I DON'T KNOW: Not explained<br>NO:
	C) ARE THE RESULTS APPLICABLE IN YOUR ENVIRONMENT?	
	9. Can these results be applied to your local environment or population?	YES: Yes<br>I DON'T KNOW:<br>NO:
	10. Were all clinically relevant results taken into account?	YES: Yes<br>I DON'T KNOW:<br>NO:
	11. Do the benefits outweigh the risks and costs?	YES: Yes<br>I DON'T KNOW:<br>NO:
CASP score	10/11	

STUDY NUMBER: 10		
TITLE: Factorial analysis of N-acetylcysteine and propolis treatment effects on symptoms, life quality and exacerbations in patients with Chronic Obstructive Pulmonary Disease (COPD): a randomized, double-blind, placebo-controlled trial.		
AUTHORS; YEAR OF PUBLICATION	V. Kolarov, J. Kotur Stevuljević, M. Ilić, M. Bogdan, B. Tušek, A. Agic, M. Dugajlić, K. Tot Vereš, S. Kutlešić Stević, B. Zvezdin.<br>2022.	
ARTICLE IDENTIFIERS	Eur Rev Med Pharmacol Sci. 2022 May;26(9):3192-3199. doi: 10.26355/eurrev_202205_28737. PMID: 35587070.	
TYPE OF STUDY	A randomized, double-blind, prospective, interventional study lasting 6 months.	
PARTICIPANTS (P)	The study included a total of 120 outpatients of both sexes, aged between 40 and 70 years, with a confirmed diagnosis of COPD and a history of the disease of at least two years, who were not allergic to N-acetylcysteine and/or propolis. The stage of the disease was assessed spirometrically, in accordance with GOLD recommendations, corresponding to stages I-IV.<br>116 patients completed the entire protocol.	
INTERVENTION (I)	Patients were divided into three groups: Group I (37 patients) received a combination of N-acetylcysteine and propolis (NACp) in powder form, at a dose of 600 mg once daily; Group II (37 patients) received 1200 mg of NACp (2x600 mg); Group III (42 patients) received a placebo.	
COMPARISON (C)	COPD patients treated with placebo.	
RESULTS (O)	Repeated measures ANOVA showed that lung function parameters, the 6-minute walk test, and the Medical Research Council dyspnea scale score did not change significantly during the study. The visual analogue scale cough scores and COPD assessment test scores varied significantly between the groups and within the experimental groups. Scores on the Leicester Cough Questionnaire and St. George's Respiratory Questionnaire did not differ between the placebo and the two study groups. However, statistically significant differences in the parameters observed during treatment were confirmed within each study group. Factor analysis and subsequent binary logistic regression revealed that the "symptom-related factor" was the most powerful predictor of exacerbation for the supplemented groups ($p < 0.01$).<br>It is evident that a six-month course of treatment involving high doses of NACp is both safe and beneficial for individuals suffering from cough and expectoration symptoms, thereby improving their quality of life. NACp has been shown to have a significant impact on the frequency of acute exacerbations in COPD patients, thanks to its ability to effectively control symptoms related to the condition.<br>Conclusions: Treatment involving high doses of NAC for a period of six months has been shown to be both safe and beneficial in terms of reducing cough and expectoration symptoms, as well as improving quality of life. NACp has been shown to have a significant impact on the frequency of acute exacerbations in COPD patients, with a proven track record of managing the symptoms that contribute to the condition.	
QUALITY OF THE ARTICLE	A) ARE THE TRIAL RESULTS VALID? (elimination questions; only if the first two questions are answered “yes” is it worth continuing to answer)	
	6. Is the trial focused on a clearly defined question?	YES: Yes<br>I DON'T KNOW:<br>NO:
	2. Was the allocation of patients to treatments random?	YES: Yes<br>I DON'T KNOW:

		NO:
	3. Were all patients who entered the study adequately considered until the end of the study?	YES: Yes<br>I DON'T KNOW:<br>NO:
	"DETAIL" QUESTIONS	
	4. Was blinding maintained for:<br>• Patients<br>• Clinicians<br>• Study staff?	YES: Yes<br>I DON'T KNOW:<br>NO:
	5. Were the groups similar at the start of the trial?	YES: Yes<br>I DON'T KNOW:<br>NO:
	6. Apart from the intervention under study, were the groups treated equally?	YES: Yes<br>I DON'T KNOW:<br>NO:
	B) WHAT ARE THE RESULTS?	
	7. Is the effect of the treatment significant?	YES: Yes<br>I DON'T KNOW:<br>NO:
	8. Was this effect accurate?	YES:<br>I DON'T KNOW: Not explianed<br>NO:
	C) ARE THE RESULTS APPLICABLE IN YOUR ENVIRONMENT?	
	9. Can these results be applied to your local environment or population?	YES: Yes<br>I DON'T KNOW:<br>NO:
	10. Were all clinically relevant results taken into account?	YES: Yes<br>I DON'T KNOW:<br>NO:
	11. Do the benefits outweigh the risks and costs?	YES: Yes<br>I DON'T KNOW:<br>NO:
CASP score	10/11	

STUDY NUMBER: 11		
TITLE: Inhaled nitric oxide improves ventilatory efficiency and exercise capacity in patients with mild COPD: A randomized-control cross-over trial.		
AUTHORS; YEAR OF PUBLICATION	Devin B Phillips, Andrew R Brotto, Bryan A Ross, Tracey L Bryan, Eric Y L Wong, Victoria L Meah, Desi P Fuhr, Sean van Diepen, Michael K Stickland.<br>2021.	
ARTICLE IDENTIFIERS	J Physiol. 2021 Mar;599(5):1665-1683. doi: 10.1113/JP280913. Epub 2021 Jan 25. PMID: 33428233.	
TYPE OF STUDY	Multigroup randomized-control cross-over study	
PARTICIPANTS (P)	15 patients with mild COPD (FEV1 = 89 ± 11% predicted) and 15 healthy controls completed symptom-limited cardiopulmonary exercise tests while breathing normoxic gas or 40 ppm iNO.	
INTERVENTION (I)		
COMPARISON (C)	Healthy individuals	
RESULTS (O)	In comparison with the placebo, iON demonstrated a substantial increase in peak oxygen uptake (1.80 ± 0.14 vs. 1.53 ± 0.10 L-min-1, $p < 0.001$) in patients diagnosed with COPD. No such effect was observed in the control group. At an equivalent work rate of 60 W, iNO reduced the ventilatory equivalent of CO2 production by 3.8 ± 4.2 units ($p = 0.002$) and dyspnea by 1.1 ± 1.2 Borg units ($p < 0.001$) in COPD patients. No effect was observed in the control group. The effects of iNO on lung volumes and oxygen saturation were consistent across both groups. In COPD patients, iNO enhanced maximum oxygen uptake by decreasing the ventilatory equivalent to CO2 production and dyspnea.	
QUALITY OF THE ARTICLE	A) ARE THE TRIAL RESULTS VALID? (elimination questions; only if the first two questions are answered “yes” is it worth continuing to answer)	
	1. Is the trial focused on a clearly defined question?	YES: Yes<br>I DON'T KNOW:<br>NO:
	2. Was the allocation of patients to treatments random?	YES: Yes<br>I DON'T KNOW:<br>NO:
	3. Were all patients who entered the study adequately considered until the end of the study?	YES: Yes<br>I DON'T KNOW:<br>NO:
	“DETAIL” QUESTIONS	
	4. Was blinding maintained for:<br>• Patients<br>• Clinicians<br>• Study staff?	YES: Yes<br>I DON'T KNOW:<br>NO:
	5. Were the groups similar at the start of the trial?	YES: Yes<br>I DON'T KNOW:<br>NO:

	6. Apart from the intervention under study, were the groups treated equally?	YES: Yes<br>I DON'T KNOW:<br>NO:
	B) WHAT ARE THE RESULTS?	
	7. Is the effect of the treatment significant?	YES: Yes<br>I DON'T KNOW:<br>NO:
	8. Was this effect accurate?	YES: Yes<br>I DON'T KNOW:<br>NO:
	C) ARE THE RESULTS APPLICABLE IN YOUR ENVIRONMENT?	
	9. Can these results be applied to your local environment or population?	YES: Yes<br>I DON'T KNOW:<br>NO:
	10. Were all clinically relevant results taken into account?	YES: Yes<br>I DON'T KNOW:<br>NO:
	11. Do the benefits outweigh the risks and costs?	YES: Yes<br>I DON'T KNOW:<br>NO:
CASP score	11/11	

STUDY NUMBER: 12		
TITLE: Iron Replacement and Redox Balance in Non-Anemic and Mildly Anemic Iron Deficiency COPD Patients: Insights from a Clinical Trial.		
AUTHORS; YEAR OF PUBLICATION	Maria Pérez-Peiró, Clara Martín-Ontiyuelo, Anna Rodó-Pi, Lucilla Piccari, Mireia Admetlló, Xavier Durán, Diego A. Rodríguez-Chiaradía and Esther Barreiro.<br>2021.	
ARTICLE IDENTIFIERS	Biomedicines. 2021 Sep 10;9(9):1191. doi: 10.3390/biomedicines9091191. PMID: 34572377; PMCID: PMC8470868.	
TYPE OF STUDY	Single-blind, single-center, parallel-group, placebo-controlled clinical trial.	
PARTICIPANTS (P)	Sixty-six patients were included (randomization 2:1): iron arm, n = 44, and placebo arm, n = 22, with similar clinical characteristics.	
INTERVENTION (I)	Patients treated with iron were administered ferric carboxymaltose solution (Ferinject®, Vifor, St. Gallen, Switzerland), 10 mL injections containing 500 mg of ferric carboxymaltose (diluted in 250 mL of 0.9% normal saline solution) or 20 mL, depending on the patient's body weight and hemoglobin levels, following the manufacturer's product label from the Spanish Medicines Agency, for 15 min while the patient was resting. In placebo patients, 250 mL of 0.9% normal saline solution was injected for 15 min, also at rest.	
COMPARISON (C)	COPD patients treated with placebo.	
RESULTS (O)	In patients treated with iron, serum levels of MDA and 3-nitrotyrosine protein adducts decreased significantly, while GSH levels increased and iron metabolism parameters improved significantly, compared to those treated with placebo. Hepcidin has been linked to iron status parameters.<br>Conclusion: The results of this randomised clinical trial demonstrated that iron replacement therapy led to a reduction in serum oxidative stress markers and an enhancement in GSH levels in patients diagnosed with stable severe COPD. Hepcidin may be a suitable surrogate biomarker of iron status and metabolism in patients with chronic respiratory diseases. These findings have the potential to inform the management of patients with severe COPD.	
QUALITY OF THE ARTICLE	A) ARE THE TRIAL RESULTS VALID? (elimination questions; only if the first two questions are answered “yes” is it worth continuing to answer)	
	1. Is the trial focused on a clearly defined question?	YES: Yes<br>I DON'T KNOW:<br>NO:
	2. Was the allocation of patients to treatments random?	YES: Yes<br>I DON'T KNOW:<br>NO:
	3. Were all patients who entered the study adequately considered until the end of the study?	YES: Yes<br>I DON'T KNOW:<br>NO:
	“DETAIL” QUESTIONS	
	4. Was blinding maintained for:<br>• Patients<br>• Clinicians	YES: Yes<br>I DON'T KNOW:<br>NO:

	• Study staff?	
	5. Were the groups similar at the start of the trial?	YES: Yes<br>I DON'T KNOW:<br>NO:
	6. Apart from the intervention under study, were the groups treated equally?	YES: Yes<br>I DON'T KNOW:<br>NO:
	B) WHAT ARE THE RESULTS?	
	7. Is the effect of the treatment significant?	YES: Yes<br>I DON'T KNOW:<br>NO:
	8. Was this effect accurate?	YES: Yes<br>I DON'T KNOW:<br>NO:
	C) ARE THE RESULTS APPLICABLE IN YOUR ENVIRONMENT?	
	9. Can these results be applied to your local environment or population?	YES: Yes<br>I DON'T KNOW:<br>NO:
	10. Were all clinically relevant results taken into account?	YES: Yes<br>I DON'T KNOW:<br>NO:
	11. Do the benefits outweigh the risks and costs?	YES: Yes<br>I DON'T KNOW:<br>NO:
CASP score	11/11	

STUDY NUMBER: 13		
TITLE: Melatonin supplementation enhances pulmonary rehabilitation outcomes in COPD: a randomized, double-blind, placebo-controlled study.		
AUTHORS; YEAR OF PUBLICATION	Soraya Maria do Nascimento Rebouças Viana, Veralice Meireles Sales de Bruin, Renata Santos Vasconcelos, Andréa Nóbrega Cirino Nogueira, Rafael Mesquita, Pedro Felipe Carvalhedo de Bruin.<br>2023.	
ARTICLE IDENTIFIERS	Respir Med. 2023 Dec;220:107441. doi: 10.1016/j.rmed.2023.107441. Epub 2023 Nov 7. PMID: 37944829.	
TYPE OF STUDY	Randomized, double-blind, placebo-controlled study	
PARTICIPANTS (P)	Thirty-nine individuals with COPD referred to a supervised pulmonary rehabilitation (PR) program at the Federal University of Ceará, Brazil, were randomized to receive melatonin (3 mg/day; n = 18) or placebo (n = 21).	
INTERVENTION (I)	The intervention group received melatonin (3 mg/day; n = 18) while the control group received a placebo (n = 21).	
COMPARISON (C)	COPD patients receiving placebo.	
RESULTS (O)	At the conclusion of the pulmonary rehabilitation programme, the melatonin group exhibited marked superiority in enhancing the distance traversed in the 6MWT (71 ± 26 vs. 25 ± 36 m; p < 0.01), health status (-11 ± 6 vs. -3 ± 5; p < 0.01), and quality of life (-6.9 ± 3.0 vs. -1.9 ± 2.4; p < 0.01) when compared to the placebo group.<br>Conclusion: 12 weeks of MLT supplementation in conjunction with PR has been shown to enhance functional capacity, health status and quality of life in patients suffering from COPD. These findings may have significant implications for the management of this condition.	
QUALITY OF THE ARTICLE	A) ARE THE TRIAL RESULTS VALID? (elimination questions; only if the first two questions are answered “yes” is it worth continuing to answer)	
	1. Is the trial focused on a clearly defined question?	YES: Yes<br>I DON'T KNOW:<br>NO:
	2. Was the allocation of patients to treatments random?	YES: Yes<br>I DON'T KNOW:<br>NO:
	3. Were all patients who entered the study adequately considered until the end of the study?	YES: Yes<br>I DON'T KNOW:<br>NO:
	“DETAIL” QUESTIONS	
	4. Was blinding maintained for:<br>• Patients<br>• Clinicians<br>• Study staff?	YES: Yes<br>I DON'T KNOW:<br>NO:
	5. Were the groups similar at the start of the trial?	YES: Yes<br>I DON'T KNOW:<br>NO:

	6. Apart from the intervention under study, were the groups treated equally?	YES: Yes<br>I DON'T KNOW:<br>NO:
	B) WHAT ARE THE RESULTS?	
	7. Is the effect of the treatment significant?	YES: Yes<br>I DON'T KNOW:<br>NO:
	8. Was this effect accurate?	YES:<br>I DON'T KNOW: Not explained<br>NO:
	C) ARE THE RESULTS APPLICABLE IN YOUR ENVIRONMENT?	
	9. Can these results be applied to your local environment or population?	YES: Yes<br>I DON'T KNOW:<br>NO:
	10. Were all clinically relevant results taken into account?	YES: Yes<br>I DON'T KNOW:<br>NO:
	11. Do the benefits outweigh the risks and costs?	YES: Yes<br>I DON'T KNOW:<br>NO:
CASP score	10/11	

STUDY NUMBER: 14		
TITLE: N-acetylcysteine Treatment in Chronic Obstructive Pulmonary Disease (COPD) and Chronic Bronchitis/Pre-COPD: Distinct Meta-analyses.		
AUTHORS; YEAR OF PUBLICATION	Alberto Papi, Franco Alfano, Tommaso Bigoni, Lorenzo Mancini, Amal Mawass, Federico Baraldi, Cristina Aljama, Marco Contoli, Marc Miravitlles.<br>2024.	
ARTICLE IDENTIFIERS	Arch Bronconeumol. 2024 May;60(5):269-278. English, Spanish. doi: 10.1016/j.arbres.2024.03.010. Epub 2024 Mar 18. PMID: 38555190.	
TYPE OF STUDY	Systematic review and meta-analysis.	
PARTICIPANTS (P)	Twenty studies were included, of which seven evaluated N-acetylcysteine (NAC) in patients with symptoms of chronic bronchitis/pre-COPD as an entry criterion.	
INTERVENTION (I)	Effects of NAC in symptomatic patients with chronic bronchitis without a diagnosis of COPD and patients with COPD. The outcomes of interest were exacerbations, respiratory symptoms, and quality of life.	
COMPARISON (C)	Placebo-treated COPD patients	
RESULTS (O)	Patients treated with NAC demonstrated a substantial decrease in the occurrence of exacerbations compared with the placebo, both in COPD (TIR = 0.76; 95% CI: 0.59-0.99) and in CB/pre-COPD (WHR = 0.81; 95% CI: 0.69-0.95). Sensitivity analyses in studies lasting longer than five months confirmed the overall results. Patients with BC/pre-COPD treated with NAC were significantly more likely to experience improvement in symptoms and/or quality of life compared with placebo (odds ratio [OR] = 3.47; 95% CI: 1.92-6.26). A similar trend was observed in the few evaluable studies on COPD. Sensitivity analyses demonstrated a significant association of NAC with improvement in symptoms and/or quality of life in both patients with CB/pre-COPD and COPD.<br>Conclusions: These findings provide novel data on NAC's efficacy in improving symptoms and quality of life, as well as its role in preventing exacerbations in COPD and CB/pre-COPD.	
QUALITY OF THE ARTICLE	A) ARE THE TRIAL RESULTS VALID? (elimination questions; only if the first two questions are answered “yes” is it worth continuing to answer)	
	7. Is the trial focused on a clearly defined question?	YES: Yes<br>I DON'T KNOW:<br>NO:
	2. Was the allocation of patients to treatments random?	YES: Yes<br>I DON'T KNOW:<br>NO:
	3. Were all patients who entered the study adequately considered until the end of the study?	YES: Yes<br>I DON'T KNOW:<br>NO:
	“DETAIL” QUESTIONS	
	4. Was blinding maintained for:<br>• Patients<br>• Clinicians<br>• Study staff?	YES: Yes<br>I DON'T KNOW:<br>NO:

	5. Were the groups similar at the start of the trial?	YES: Yes<br>I DON'T KNOW:<br>NO:
	6. Apart from the intervention under study, were the groups treated equally?	YES: Yes<br>I DON'T KNOW:<br>NO:
	B) WHAT ARE THE RESULTS?	
	7. Is the effect of the treatment significant?	YES: Yes<br>I DON'T KNOW:<br>NO:
	8. Was this effect accurate?	YES: Yes<br>I DON'T KNOW:<br>NO:
	C) ARE THE RESULTS APPLICABLE IN YOUR ENVIRONMENT?	
	9. Can these results be applied to your local environment or population?	YES: Yes<br>I DON'T KNOW:<br>NO:
	10. Were all clinically relevant results taken into account?	YES: Yes<br>I DON'T KNOW:<br>NO:
	11. Do the benefits outweigh the risks and costs?	YES: Yes<br>I DON'T KNOW:<br>NO:
CASP score	11/11	

<table>
<tr><td colspan="3">STUDY NUMBER: 15</td></tr>
<tr><td colspan="3">TITLE: Oxidative Stress and Total Phenolics Concentration in COPD Patients-The Effect of Exercises: A Randomized Controlled Trial.</td></tr>
<tr><td>AUTHORS; YEAR OF PUBLICATION</td><td colspan="2">Katarzyna Domaszewska, Sara Górna, Malwina Pietrzak and Tomasz Podgórski. 2022.</td></tr>
<tr><td>ARTICLE IDENTIFIERS</td><td colspan="2">Nutrients. 2022 May 6;14(9):1947. doi: 10.3390/nu14091947. PMID: 35565914; PMCID: PMC9105366.</td></tr>
<tr><td>TYPE OF STUDY</td><td colspan="2">Randomized Clinical Trial</td></tr>
<tr><td>PARTICIPANTS (P)</td><td colspan="2">32 patients (16 men and 16 women). 20 in the intervention group and 12 in the control group.</td></tr>
<tr><td>INTERVENTION (I)</td><td colspan="2">Twenty randomly selected individuals underwent a modified rehabilitation program during their stay in rehabilitation, and the results obtained were compared with those of 12 patients (control group) who received treatment without resistance training. Spirometry and cardiopulmonary exercise testing (CPET) were performed at the beginning and end of the study. Venous blood was tested for concentrations of oxidative stress parameters (allantoin (All) and substances that react with thiobarbituric acid) and antioxidants (plasma ferric reducing capacity and total phenols).</td></tr>
<tr><td>COMPARISON (C)</td><td colspan="2">Control group of COPD patients receiving treatment without resistance training</td></tr>
<tr><td>RESULTS (O)</td><td colspan="2">In the study group, there was a significant increase in VO2max ($p = 0.0702$) and FEV1/FVC ($p < 0.05$; ES: 0.436) after training. The CPET applied at each time point caused an increase in All concentration ($p < 0.05$) in both the study and control groups. Conclusions: It is evident that endurance training applied as part of the rehabilitation process did not cause any additional aggravation of oxidative stress or an increase in the total phenolics concentration of the blood.</td></tr>
<tr><td rowspan="7">QUALITY OF THE ARTICLE</td><td colspan="2">A) ARE THE TRIAL RESULTS VALID? (elimination questions; only if the first two questions are answered “yes” is it worth continuing to answer)</td></tr>
<tr><td>1. Is the trial focused on a clearly defined question?</td><td>YES: Yes
I DON'T KNOW:
NO:</td></tr>
<tr><td>2. Was the allocation of patients to treatments random?</td><td>YES: Yes
I DON'T KNOW:
NO:</td></tr>
<tr><td>3. Were all patients who entered the study adequately considered until the end of the study?</td><td>YES: Yes.
I DON'T KNOW:
NO:</td></tr>
<tr><td colspan="2">“DETAIL” QUESTIONS</td></tr>
<tr><td>4. Was blinding maintained for:
• Patients
• Clinicians
• Study staff?</td><td>YES: Yes
I DON'T KNOW:
NO:</td></tr>
<tr><td>5. Were the groups similar at the start of the trial?</td><td>YES: Yes
I DON'T KNOW:</td></tr>
</table>

		NO:
	6. Apart from the intervention under study, were the groups treated equally?	YES: Yes<br>I DON'T KNOW:<br>NO:
	B) WHAT ARE THE RESULTS?	
	7. Is the effect of the treatment significant?	YES: Yes<br>I DON'T KNOW:<br>NO:
	8. Was this effect accurate?	YES:<br>I DON'T KNOW: Not explained<br>NO:
	C) ARE THE RESULTS APPLICABLE IN YOUR ENVIRONMENT?	
	9. Can these results be applied to your local environment or population?	YES: Yes<br>I DON'T KNOW:<br>NO:
	10. Were all clinically relevant results taken into account?	YES: Yes<br>I DON'T KNOW:<br>NO:
	11. Do the benefits outweigh the risks and costs?	YES: Yes<br>I DON'T KNOW:<br>NO:
CASP score	11/11	

<table>
<tr><td colspan="3">STUDY NUMBER: 16</td></tr>
<tr><td colspan="3">TITLE: Residual effects of 12 weeks of power-oriented resistance training plus high-intensity interval training on muscle dysfunction, systemic oxidative damage, and antioxidant capacity after 10 months of training cessation in older people with COPD.</td></tr>
<tr><td>AUTHORS; YEAR OF PUBLICATION</td><td colspan="2">Ivan Baltasar-Fernandez, Jose Losa-Reyna, Aitor Carretero, Carlos Rodriguez-Lopez, Ana Alfaro-Acha, Amelia Guadalupe-Grau, Ignacio Ara, Luis M Alegre, Mari Carmen Gomez-Cabrera, Francisco J García-García, Julian Alcazar.
2023.</td></tr>
<tr><td>ARTICLE IDENTIFIERS</td><td colspan="2">Scand J Med Sci Sports. 2023 Sep;33(9):1661-1676. doi: 10.1111/sms.14428. Epub 2023 Jun 15. PMID: 37322570.</td></tr>
<tr><td>TYPE OF STUDY</td><td colspan="2">Randomized controlled trial with two parallel groups.</td></tr>
<tr><td>PARTICIPANTS (P)</td><td colspan="2">A total of 21 older adults with COPD.
Intervention group, n = 8
Control group, n = 13
Participants of the intervention group participated in a 12-week concurrent training program while the control group received no intervention (i.e., usual care) during the same time period.</td></tr>
<tr><td>INTERVENTION (I)</td><td colspan="2">COPD patients from both intervention and contro groups were assessed at baseline and 10 months after the end of the intervention using the Short Physical Performance Battery, health-related quality of life, vastus lateralis muscle thickness, maximum pulmonary oxygen consumption and maximum work rate, early and late isometric rate of force development, maximum muscle power in leg press and chest press , and systemic oxidative damage and antioxidant capacity.</td></tr>
<tr><td>COMPARISON (C)</td><td colspan="2">COPD patients that didn´t do the concurrent training program.</td></tr>
<tr><td>RESULTS (O)</td><td colspan="2">Following a 10-month period of detraining, the INT group demonstrated an enhancement in key performance metrics, as evidenced by an increase in the Short Physical Performance Battery (Δ = 1.0 point), health-related quality of life (Δ = 0.07 points), early RFD (Δ = 834 $N \cdot s^{-1}$), LPmax (Δ = 62.2 W), and CPmax (Δ = 16.0 W). These statistically significant findings were observed at a significance level of $p < 0.05$. In addition, a positive effect was observed in the intervention group compared to the control group with regard to MT and Wpeak (both $p < 0.05$). No differences between groups were recorded in VO2 max, late RFD, systemic oxidative damage, and antioxidant capacity from baseline to 10 months after the end of the intervention (all $p > 0.05$).
Conclusions: Twelve weeks of concurrent training was sufficient to ensure improved physical function, health-related quality of life, early RFD and maximum muscle power. Furthermore, the programme was able to preserve MT and Wpeak, but not peak VO2, late RFD, systemic oxidative damage and antioxidant capacity in the subsequent 10 months of detraining in older adults with COPD.</td></tr>
<tr><td rowspan="3">QUALITY OF THE ARTICLE</td><td colspan="2">A) ARE THE TRIAL RESULTS VALID? (elimination questions; only if the first two questions are answered “yes” is it worth continuing to answer)</td></tr>
<tr><td>8. Is the trial focused on a clearly defined question?</td><td>YES: Yes
I DON'T KNOW:
NO:</td></tr>
<tr><td>2. Was the allocation of patients to treatments random?</td><td>YES: Yes
I DON'T KNOW:
NO:</td></tr>
</table>

	3. Were all patients who entered the study adequately considered until the end of the study?	YES: Yes<br>I DON'T KNOW:<br>NO:
	"DETAIL" QUESTIONS	
	4. Was blinding maintained for:<br>• Patients<br>• Clinicians<br>• Study staff?	YES: Yes<br>I DON'T KNOW:<br>NO:
	5. Were the groups similar at the start of the trial?	YES: Yes<br>I DON'T KNOW:<br>NO:
	6. Apart from the intervention under study, were the groups treated equally?	YES: Yes<br>I DON'T KNOW:<br>NO:
	B) WHAT ARE THE RESULTS?	
	7. Is the effect of the treatment significant?	YES: Yes<br>I DON'T KNOW:<br>NO:
	8. Was this effect accurate?	YES:<br>I DON'T KNOW: Not explained<br>NO:
	C) ARE THE RESULTS APPLICABLE IN YOUR ENVIRONMENT?	
	9. Can these results be applied to your local environment or population?	YES: Yes<br>I DON'T KNOW:<br>NO:
	10. Were all clinically relevant results taken into account?	YES: Yes<br>I DON'T KNOW:<br>NO:
	11. Do the benefits outweigh the risks and costs?	YES: Yes<br>I DON'T KNOW:<br>NO:
CASP score	10/11	

STUDY NUMBER: 17	
TITLE: Resveratrol and metabolic health in COPD: A proof-of-concept randomized controlled trial.	
AUTHORS; YEAR OF PUBLICATION	Rosanne Jhcg Beijers, Harry R Gosker, Karin Jc Sanders, Chiel de Theije, Marco Kelders, Gerard Clarke, John F Cryan, Bram van den Borst, Annemie Mwj Schols. 2020.
ARTICLE IDENTIFIERS	Clin Nutr. 2020 Oct;39(10):2989-2997. doi: 10.1016/j.clnu.2020.01.002. Epub 2020 Jan 13. PMID: 31996311.
TYPE OF STUDY	Double-blind, randomized, placebo-controlled proof-of-concept study.
PARTICIPANTS (P)	21 COPD patients receiving resveratrol (n = 11) or placebo (n = 10).
INTERVENTION (I)	Twenty-one patients with COPD received resveratrol (150 mg/day) or placebo for four weeks. Before and after the intervention, blood samples, quadriceps muscle samples, and subcutaneous abdominal fat biopsies were obtained to perform metabolic and inflammatory profiling. Body composition was assessed using dual-energy X-ray absorptiometry.
COMPARISON (C)	COPD patients treated with placebo.
RESULTS (O)	The regulators of muscle mitochondrial biogenesis (AMPK, SIRT1, and PGC-1α), as well as mitochondrial respiration (Oxphos complexes), oxidative enzyme activities, and kynurenine aminotransferases did not improve with resveratrol. High-sensitivity C-reactive protein in plasma and kynurenine levels remained unchanged following resveratrol supplementation. Inflammatory markers in adipose tissue were not affected by resveratrol, while markers of glycolysis and lipolysis increased significantly compared to the placebo. Following resveratrol supplementation, a decrease in body weight was observed (resveratrol: -0.95 +/-1.01 kg vs. placebo: -0.16 +/- 0.66 kg, p = 0.049). This was attributed to a reduction in lean mass (resveratrol: -1.79 ± 1.67 kg vs. 0.37 ± 0.86 kg, p = 0.026).<br>Conclusion: We do not confirm previously reported positive effects of resveratrol on skeletal muscle mitochondrial function in patients with COPD, but show an unexpected decline in lean mass.
QUALITY OF THE ARTICLE	A) ARE THE TRIAL RESULTS VALID? (elimination questions; only if the first two questions are answered “yes” is it worth continuing to answer)

1. Is the trial focused on a clearly defined question?	YES: Yes<br>I DON'T KNOW:<br>NO:
2. Was the allocation of patients to treatments random?	YES: Yes<br>I DON'T KNOW:<br>NO:
3. Were all patients who entered the study adequately considered until the end of the study?	YES: Yes<br>I DON'T KNOW:<br>NO:
“DETAIL” QUESTIONS	
4. Was blinding maintained for:<br>• Patients	YES: Yes<br>I DON'T KNOW:

	• Clinicians<br>• Study staff?	NO:
	5. Were the groups similar at the start of the trial?	YES: Yes<br>I DON'T KNOW:<br>NO:
	6. Apart from the intervention under study, were the groups treated equally?	YES: Yes<br>I DON'T KNOW:<br>NO:
	B) WHAT ARE THE RESULTS?	
	7. Is the effect of the treatment significant?	YES: Very weak statistical significance<br>I DON'T KNOW:<br>NO:
	8. Was this effect accurate?	YES:<br>I DON'T KNOW: Not explained.<br>NO:
	C) ARE THE RESULTS APPLICABLE IN YOUR ENVIRONMENT?	
	9. Can these results be applied to your local environment or population?	YES: Yes<br>I DON'T KNOW:<br>NO:
	10. Were all clinically relevant results taken into account?	YES: Yes<br>I DON'T KNOW:<br>NO:
	11. Do the benefits outweigh the risks and costs?	YES: Yes<br>I DON'T KNOW:<br>NO:
CASP score	9/11	

STUDY NUMBER: 18		
TITLE: Sequential inspiratory muscle exercise-noninvasive positive pressure ventilation alleviates oxidative stress in COPD by mediating SOCS5/JAK2/STAT3 pathway		
AUTHORS; YEAR OF PUBLICATION	Yirou Lei, Jiaying He, Fang Hu, Hao Zhu, Jing Gu, Lijuan Tang, Man Luo.<br>2023..	
ARTICLE IDENTIFIERS	BMC Pulm Med. 2023 Oct 12;23(1):385. doi: 10.1186/s12890-023-02656-5. PMID: 37828534; PMCID: PMC10568888.	
TYPE OF STUDY	Randomized Clinical Trial	
PARTICIPANTS (P)	100 COPD patients	
INTERVENTION (I)	A total of 100 COPD patients were enrolled and randomly divided into oxygen therapy (OT), noninvasive positive pressure ventilation (NIPPV), inspiratory muscle training (IMT), and sequential (NIPPV+IMT) groups. Lung function, exercise tolerance, quality of life, and dyspnea symptoms were examined and recorded. Next, levels of reactive oxygen species (ROS), malondialdehyde (MDA), superoxide dismutase (SOD), and glutathione (GSH) were detected using an immunoenzymatic assay, and changes in the expression of cytokine signaling suppressor 5 (SOCS5)/Janus kinase 2 (JAK2)/signal transducer and activator of transcription 3 (STAT3) signaling pathway were detected by quantitative real-time polymerase chain reaction (qRT-PCR) and Western blot. A mouse model of COPD was then established to further verify the effects of the SOCS5/JAK2/STAT3 pathways on lung function and oxidative stress.	
COMPARISON (C)	Comparison between groups	
RESULTS (O)	Following eight weeks of treatment, NIPPV, IMT, or sequential (NIPPV+IMT) significantly improved exercise endurance, quality of life, and dyspnea, reduced oxidative stress, promoted SOCS5 expression, and inhibited JAK2/STAT3 pathway activation, with no significant effect on lung function in COPD patients. It is worth noting that sequential treatment (NIPPV+IMT) demonstrated superior therapeutic outcomes in comparison to IMT or NIPPV administered individually. Furthermore, the results of experiments on animals demonstrated that SOCS5 overexpression led to a significant reduction in pulmonary inflammatory infiltration, pathological changes, and oxidative stress levels in mice with COPD. In addition, the study showed improvements in lung function and inhibition of JAK2/STAT3 pathway activation.<br>It is concluded that sequential NIPPV + IMT significantly reduced the development of COPD by regulating the SOCS5/JAK2/STAT3 signalling-mediated oxidative stress pathway.<br>Conclusion: Our results demonstrate that sequential (NIPPV + IMT) therapy significantly reduces the development of COPD by regulating the SOCS5/JAK2/STAT3 signalling pathway, which is associated with oxidative stress.	
QUALITY OF THE ARTICLE	A) ARE THE TRIAL RESULTS VALID? (elimination questions; only if the first two questions are answered “yes” is it worth continuing to answer)	
	9. Is the trial focused on a clearly defined question?	YES: Yes<br>I DON'T KNOW:<br>NO:
	2. Was the allocation of patients to treatments random?	YES: Yes<br>I DON'T KNOW:<br>NO:

	3. Were all patients who entered the study adequately considered until the end of the study?	YES: Yes<br>I DON'T KNOW:<br>NO:
	"DETAIL" QUESTIONS	
	4. Was blinding maintained for:<br>• Patients<br>• Clinicians<br>• Study staff?	YES:<br>I DON'T KNOW: No blinding<br>NO:
	5. Were the groups similar at the start of the trial?	YES: Yes<br>I DON'T KNOW:<br>NO:
	6. Apart from the intervention under study, were the groups treated equally?	YES: Yes<br>I DON'T KNOW:<br>NO:
	B) WHAT ARE THE RESULTS?	
	7. Is the effect of the treatment significant?	YES: Yes<br>I DON'T KNOW:<br>NO:
	8. Was this effect accurate?	YES: Yes<br>I DON'T KNOW:<br>NO:
	C) ARE THE RESULTS APPLICABLE IN YOUR ENVIRONMENT?	
	9. Can these results be applied to your local environment or population?	YES: Yes<br>I DON'T KNOW:<br>NO:
	10. Were all clinically relevant results taken into account?	YES: Yes<br>I DON'T KNOW:<br>NO:
	11. Do the benefits outweigh the risks and costs?	YES: Yes<br>I DON'T KNOW:<br>NO:
CASP score	9/11	

STUDY NUMBER: 19		
TITLE: Therapeutic effects of black seed oil supplementation on chronic obstructive pulmonary disease patients: A randomized controlled double blind clinical trial.		
AUTHORS; YEAR OF PUBLICATION	Mahmood A. Al-Azzawi, Mohamed M.N. AboZaid, Reda Abdel Latif Ibrahem, Moustafa A. Sakr<br>2020.	
ARTICLE IDENTIFIERS	Heliyon. 2020 Aug 13;6(8):e04711. doi: 10.1016/j.heliyon.2020.e04711. PMID: 32904114; PMCID: PMC7452452.	
TYPE OF STUDY	Prospective, randomized, controlled, double-blind clinical trial.	
PARTICIPANTS (P)	47 patients in the black seed oil (BSO) group and 44 patients in the control group.	
INTERVENTION (I)	Control group: Received standard COPD medication only (inhaled corticosteroids with a long-acting beta-2 agonist [LABA], specifically 50 µg of salmeterol and 500 µg of fluticasone propionate, via one inhaler twice daily).<br>BSO group: Treated with standard COPD medication plus an oral dose of 1 g twice daily of 100% pure, cold-pressed black cumin oil in soft gel capsules containing a minimum of 0.95% natural thymoquinone as a complementary treatment.	
COMPARISON (C)	Control group (indicated above)	
RESULTS (O)	The baseline level of thiobarbituric acid reactive substances (TBARS) in the control group was 6.74 ± 1.28 nmol MDA/mL. This decreased slightly to 6.24 ± 1.29 nmol MDA/mL ($p = 0.06$) after three months of standard treatment. In the BSO group, the initial level was 7.20 ± 1.19 nmol MDA/mL. This decreased significantly to 3.76 ± 0.70 nmol MDA/mL ($p < 0.001$) after three months of standard treatment plus intervention. The results showed a significant decrease in TBARS values, with the BSO group exhibiting a 46.44% reduction compared to a 5.71% decrease in the control group ($p < 0.001$).<br>Conclusion: Administering black seed oil as a supplement may be an effective adjunct therapy for improving pulmonary function, reducing inflammation and balancing oxidant and antioxidant levels in patients with COPD.	
QUALITY OF THE ARTICLE	A) ARE THE TRIAL RESULTS VALID? (elimination questions; only if the first two questions are answered “yes” is it worth continuing to answer)	
	10. Is the trial focused on a clearly defined question?	YES: Yes<br>I DON'T KNOW:<br>NO:
	2. Was the allocation of patients to treatments random?	YES: Yes<br>I DON'T KNOW:<br>NO:
	3. Were all patients who entered the study adequately considered until the end of the study?	YES: Yes<br>I DON'T KNOW:<br>NO:
	“DETAIL” QUESTIONS	
	4. Was blinding maintained for:<br>• Patients<br>• Clinicians	YES: Yes<br>I DON'T KNOW:<br>NO:

	• Study staff?	
	5. Were the groups similar at the start of the trial?	YES: Yes<br>I DON'T KNOW:<br>NO:
	6. Apart from the intervention under study, were the groups treated equally?	YES: Yes<br>I DON'T KNOW:<br>NO:
	B) WHAT ARE THE RESULTS?	
	7. Is the effect of the treatment significant?	YES: Yes<br>I DON'T KNOW:<br>NO:
	8. Was this effect accurate?	YES:<br>I DON'T KNOW: Not explained<br>NO:
	C) ARE THE RESULTS APPLICABLE IN YOUR ENVIRONMENT?	
	9. Can these results be applied to your local environment or population?	YES: Yes<br>I DON'T KNOW:<br>NO:
	10. Were all clinically relevant results taken into account?	YES: Yes<br>I DON'T KNOW:<br>NO:
	11. Do the benefits outweigh the risks and costs?	YES: Yes<br>I DON'T KNOW:<br>NO:
CASP score	10/11	

<table>
<tr><td colspan="3">STUDY NUMBER: 20</td></tr>
<tr><td colspan="3">TITLE: Withania somnifera (L.) Dunal as Add-On Therapy for COPD Patients: A Randomized, Placebo-Controlled, Double-Blind Study</td></tr>
<tr><td>AUTHORS; YEAR OF PUBLICATION</td><td colspan="2">Priyam Singh, Khushtar Anwar Salman, Mohammad Shameem, Mohd Sharib Warsi. 2022.</td></tr>
<tr><td>ARTICLE IDENTIFIERS</td><td colspan="2">Front Pharmacol. 2022 Jun 16;13:901710. doi: 10.3389/fphar.2022.901710. PMID: 35784687; PMCID: PMC9243480.</td></tr>
<tr><td>TYPE OF STUDY</td><td colspan="2">Randomized, placebo-controlled, double-blind clinical trial</td></tr>
<tr><td>PARTICIPANTS (P)</td><td colspan="2">A total of 150 patients were randomly assigned to one of three groups: a control group, a placebo group, and a group taking Withania somnifera (L.) Dunal (WS).</td></tr>
<tr><td>INTERVENTION (I)</td><td colspan="2">The control group received conventional medication only, i.e. fluticasone propionate, oral prednisolone, and a long-acting bronchodilator (Tiate or doxiflo) as required. The placebo group received starch capsules alongside conventional medications. The WS group took WS root capsules alongside conventional medications for 12 weeks.</td></tr>
<tr><td>COMPARISON (C)</td><td colspan="2">Control group receiving conventional COPD medication</td></tr>
<tr><td>RESULTS (O)</td><td colspan="2">The WS group demonstrated enhancements in lung function, quality of life, and exercise tolerance, accompanied by a substantial reduction in inflammation. The present study found that systemic oxidative stress decreased significantly only in this group. Although a minor placebo effect was observed in the SGRQ test, this was not seen in other tests. Research has demonstrated that the withanolides present in WS roots exhibit significant inhibitory activity against ACE-2, MPO and IL-6 proteins. This activity has been shown to exceed that of a standard drug or a known inhibitor. Furthermore, predicted FEV1% demonstrated a significant correlation with systemic antioxidant status (positive correlation) and malondialdehyde (MDA, negative correlation), suggesting that the antioxidant potential of WS contributes significantly to improved lung function.
Conclusion: The present study has demonstrated, through clinical experimentation, that the administration of WS root in conjunction with conventional pharmaceuticals results in a significant amelioration of COPD symptoms, particularly in patients classified within GOLD categories 2 and 3. In silico analysis has revealed the compound to be a potent inhibitor of the SARS-CoV-2 receptor, ACE-2, as well as the enzymes MPO and IL-6.</td></tr>
<tr><td rowspan="5">QUALITY OF THE ARTICLE</td><td colspan="2">A) ARE THE TRIAL RESULTS VALID? (elimination questions; only if the first two questions are answered “yes” is it worth continuing to answer)</td></tr>
<tr><td>11. Is the trial focused on a clearly defined question?</td><td>YES: Yes
I DON'T KNOW:
NO:</td></tr>
<tr><td>2. Was the allocation of patients to treatments random?</td><td>YES: Yes
I DON'T KNOW:
NO:</td></tr>
<tr><td>3. Were all patients who entered the study adequately considered until the end of the study?</td><td>YES: Yes
I DON'T KNOW:
NO:</td></tr>
<tr><td colspan="2">“DETAIL” QUESTIONS</td></tr>
</table>

	4. Was blinding maintained for:<br>• Patients<br>• Clinicians<br>• Study staff?	YES: Yes<br>I DON'T KNOW:<br>NO:
	5. Were the groups similar at the start of the trial?	YES: Yes<br>I DON'T KNOW:<br>NO:
	6. Apart from the intervention under study, were the groups treated equally?	YES: Yes<br>I DON'T KNOW:<br>NO:
	B) WHAT ARE THE RESULTS?	
	7. Is the effect of the treatment significant?	YES: Yes<br>I DON'T KNOW:<br>NO:
	8. Was this effect accurate?	YES:<br>I DON'T KNOW: Not explained<br>NO:
	C) ARE THE RESULTS APPLICABLE IN YOUR ENVIRONMENT?	
	9. Can these results be applied to your local environment or population?	YES: Yes<br>I DON'T KNOW:<br>NO:
	10. Were all clinically relevant results taken into account?	YES: Yes<br>I DON'T KNOW:<br>NO:
	11. Do the benefits outweigh the risks and costs?	YES: Yes<br>I DON'T KNOW:<br>NO:
CASP score	10/11	

<table>
<tr><td colspan="3">STUDY NUMBER: 21</td></tr>
<tr><td colspan="3">TITLE: Zataria multiflora affects pulmonary function tests, respiratory symptoms, bronchodilator drugs use and hematological parameters in chronic obstructive pulmonary disease patients: A randomized doubled-blind clinical trial</td></tr>
<tr><td>AUTHORS; YEAR OF PUBLICATION</td><td colspan="2">Vahideh Ghorani, Omid Rajabi, Majid Mirsadraee, Mahnaz Amini, Shadi Ghaffari, Mohammad Hossein Boskabady. 2024.</td></tr>
<tr><td>ARTICLE IDENTIFIERS</td><td colspan="2">J Ethnopharmacol. 2024 May 23;326:117928. doi: 10.1016/j.jep.2024.117928. Epub 2024 Feb 17. PMID: 38373666.</td></tr>
<tr><td>TYPE OF STUDY</td><td colspan="2">Randomized, double-blind clinical trial.</td></tr>
<tr><td>PARTICIPANTS (P)</td><td colspan="2">Forty-five patients were randomly assigned to one of three groups: a placebo group (P), and groups receiving 3 mg/kg/day or 6 mg/kg/day of Z. multiflora extract (Z3 and Z6, respectively).</td></tr>
<tr><td>INTERVENTION (I)</td><td colspan="2">The present study investigates the administration of adjuvant treatment to the usual treatment for COPD with placebo, 3mg/kg or 6mg/kg of Z. multiflora. FEV1 and MEF25-75, respiratory symptoms, inhaled bronchodilator drugs use and hematological factors were evaluated before and 1-2 months after treatment.</td></tr>
<tr><td>COMPARISON (C)</td><td colspan="2">Placebo group</td></tr>
<tr><td>RESULTS (O)</td><td colspan="2">The administration of Z. multiflora resulted in a significant improvement in FEV1 ($p < 0.05$ to $p < 0.01$). Furthermore, a significant amelioration of respiratory symptoms was observed following the administration of extracts over a period of one to two months, as evidenced by a p-value ranging from $p < 0.05$ to $p < 0.001$ when compared to the initial baseline values. In the groups receiving extract, there was a significant decrease in the use of inhaled bronchodilator medications at the conclusion of the study (both, $p < 0.05$). A reduction in total leukocytes was observed 1–2 months after treatment in the groups treated with extract compared with baseline values ($p < 0.05$ to $p < 0.001$). A significant decrease in neutrophil levels was observed in the Z3 and Z6 groups after a 2-month period of treatment, in comparison to the levels recorded after 1 month of treatment ($p < 0.05$ to $p < 0.01$).
Conclusion: It can be concluded from the evidence presented that this herbal remedy exerts a therapeutic effect on patients suffering from chronic obstructive pulmonary disease. This effect can be attributed to the properties of the plant which are effective in reducing inflammation.</td></tr>
<tr><td rowspan="5">QUALITY OF THE ARTICLE</td><td colspan="2">A) ARE THE TRIAL RESULTS VALID? (elimination questions; only if the first two questions are answered “yes” is it worth continuing to answer)</td></tr>
<tr><td>12. Is the trial focused on a clearly defined question?</td><td>YES: Yes
I DON'T KNOW:
NO:</td></tr>
<tr><td>2. Was the allocation of patients to treatments random?</td><td>YES: Yes
I DON'T KNOW:
NO:</td></tr>
<tr><td>3. Were all patients who entered the study adequately considered until the end of the study?</td><td>YES: Yes
I DON'T KNOW:
NO:</td></tr>
<tr><td colspan="2">“DETAIL” QUESTIONS</td></tr>
</table>

	4. Was blinding maintained for:<br>• Patients<br>• Clinicians<br>• Study staff?	YES: Yes<br>I DON'T KNOW:<br>NO:
	5. Were the groups similar at the start of the trial?	YES: Yes<br>I DON'T KNOW:<br>NO:
	6. Apart from the intervention under study, were the groups treated equally?	YES: Yes<br>I DON'T KNOW:<br>NO:
	B) WHAT ARE THE RESULTS?	
	7. Is the effect of the treatment significant?	YES: Yes<br>I DON'T KNOW:<br>NO:
	8. Was this effect accurate?	YES:<br>I DON'T KNOW: Not explained<br>NO:
	C) ARE THE RESULTS APPLICABLE IN YOUR ENVIRONMENT?	
	9. Can these results be applied to your local environment or population?	YES: Yes<br>I DON'T KNOW:<br>NO:
	10. Were all clinically relevant results taken into account?	YES: Yes<br>I DON'T KNOW:<br>NO:
	11. Do the benefits outweigh the risks and costs?	YES: Yes<br>I DON'T KNOW:<br>NO:
CASP score	10/11	

STUDY NUMBER: 22		
TITLE: Efficacy and safety of dietary polyphenol supplements for COPD: a systematic review and meta-analysis.		
AUTHORS; YEAR OF PUBLICATION	Wu D, Dong Y, Zhang D,<br>2022	
ARTICLE IDENTIFIERS	Front Immunol. 2025 Jul 23;16:1617694. doi: 10.3389/fimmu.2025.1617694. PMID: 40771814; PMCID: PMC12325041.	
TYPE OF STUDY	Systematic review and meta-analysis	
PARTICIPANTS (P)	COPD patients (N=894). Intervention group (N=452), Comparison group (N=442).	
INTERVENTION (I)	Dietary polyphenols: The RCTs (N=15) focused on this study involved dietary supplementation with 8 polyphenols: curcumin, resveratrol, anthocyanins, quercetin, salidroside, dietary beetroot juice, pomegranate juice and adjunctive treatment with oral AKL1.	
COMPARISON (C)	Placebo group (N=442)	
RESULTS (O)	The results show that most of the dietary polyphenol components included can be used safely and have significant efficacy in relieving clinical symptoms, restoring respiratory function, and inhibiting inflammatory responses.	
QUALITY OF THE ARTICLE	A) ARE THE TRIAL RESULTS VALID? (elimination questions; only if the first two questions are answered “yes” is it worth continuing to answer)	
	1. Is the trial focused on a clearly defined question?	YES: Yes<br>I DON'T KNOW:<br>NO:
	2. Was the allocation of patients to treatments random?	YES: Yes<br>I DON'T KNOW:<br>NO:
	3. Were all patients who entered the study adequately considered until the end of the study?	YES: Yes<br>I DON'T KNOW:<br>NO:
	“DETAIL” QUESTIONS	
	4. Was blinding maintained for:<br>• Patients<br>• Clinicians<br>• Study staff?	YES: Yes<br>I DON'T KNOW:<br>NO:
	5. Were the groups similar at the start of the trial?	YES: Yes<br>I DON'T KNOW:<br>NO:
	6. Apart from the intervention under study, were the groups treated equally?	YES: Yes<br>I DON'T KNOW:<br>NO:

	B) WHAT ARE THE RESULTS?	
	7. Is the effect of the treatment significant?	YES: Yes<br>I DON'T KNOW:<br>NO:
	8. Was this effect accurate?	YES: Yes<br>I DON'T KNOW:<br>NO:
	C) ARE THE RESULTS APPLICABLE IN YOUR ENVIRONMENT?	
	9. Can these results be applied to your local environment or population?	YES: Yes<br>I DON'T KNOW:<br>NO:
	10. Were all clinically relevant results taken into account?	YES: Yes<br>I DON'T KNOW:<br>NO:
	11. Do the benefits outweigh the risks and costs?	YES: Yes<br>I DON'T KNOW:<br>NO:
CASP score	11/11	
