# Supplementary material for: Antioxidants as Therapeutic Tools in the Management of COPD: A Systematic Review with Meta-Analysis
Source: Antioxidants (Basel). 2026 Apr 2;15(4):446. doi: 10.3390/antiox15040446 (PMC13113252; doi:10.3390/antiox15040446)
Supplement: Supplementary file 1 [file antioxidants-15-00446-s001.zip › Supplementary Table S6.pdf]

Supplementary Table S6. Meta-analysis of exacerbations in COPD

Study (ID)	Intervention	Relative Risk	95% CI*
Buha 2022 (ID 1)	NAC + propolis	0.65	0.42–1.01
Zhou 2024 (ID 7)	NAC	0.90	0.80–1.02
Kolarov 2022 (ID 9)	NAC + propolis	0.72	0.54–0.96
Pooled (Random Effects)	-	0.80	0.66–0.98

* 95% CI: 95% Confidence Interval
